# Operational feasibility of *Plasmodium vivax* radical cure with tafenoquine or primaquine following point-of-care, quantitative glucose-6-phosphate dehydrogenase testing in the Brazilian Amazon: a real-life retrospective analysis

**DOI:** 10.1016/S2214-109X(23)00542-9

**Published:** 2024-02-14

**Authors:** Marcelo Brito, Rosilene Rufatto, Felipe Murta, Vanderson Sampaio, Patrícia Balieiro, Djane Baía-Silva, Vanessa Castro, Brenda Alves, Aline Alencar, Stephan Duparc, Penny Grewal Daumerie, Isabelle Borghini-Fuhrer, Elodie Jambert, Cássio Peterka, Francisco Edilson Lima, Leonardo Carvalho Maia, Catherine Lucena Cruz, Bruna Maciele, Mariana Vasconcelos, Myrna Machado, Elder Augusto Figueira, Antônio Alcirley Balieiro, Alexandre Menezes, Roberta Ataídes, Dhelio Batista Pereira, Marcus Lacerda

**Affiliations:** a Fundação de Medicina Tropical Doutor Heitor Vieira Dourado, Manaus, Brazil; b Centro de Pesquisa em Medicina Tropical de Rondônia, Porto Velho, Brazil; c Universidade do Estado do Amazonas, Manaus, Brazil; d Instituto Leônidas & Maria Deane, Fiocruz, Manaus, Brazil; e Universidade Nilton Lins, Manaus, Brazil; f Medicines for Malaria Venture, Geneva, Switzerland; g Brazilian Ministry of Health, Brasília, Brazil; h Fundação de Vigilância em Saúde, Manaus, Brazil; i Global Health Strategies, Rio de Janeiro, Brazil; j University of Texas Medical Branch, Galveston, TX, USA

## Abstract

**Background:**

To achieve malaria elimination, Brazil must implement *Plasmodium vivax* radical cure. We aimed to investigate the operational feasibility of point-of-care, quantitative, glucose-6-phosphate dehydrogenase (G6PD) testing followed by chloroquine plus tafenoquine or primaquine.

**Methods:**

This non-interventional, observational study was done at 43 health facilities in Manaus (Amazonas State) and Porto Velho (Rondônia State), Brazil, implementing a new *P vivax* treatment algorithm incorporating point-of-care quantitative G6PD testing to identify G6PD status and single-dose tafenoquine (G6PD normal, aged ≥16 years, and not pregnant or breastfeeding) or primaquine (intermediate or normal G6PD, aged ≥6 months, not pregnant, or breastfeeding >1 month). Following training of health-care providers, we collated routine patient records from the malaria epidemiological surveillance system (SIVEP-Malaria) retrospectively for all consenting patients aged at least 6 months with parasitologically confirmed *P vivax* malaria mono-infection or *P vivax* plus *P falciparum* mixed infection, presenting between Sept 9, 2021, and Aug 31, 2022. The primary endpoint was the proportion of patients aged at least 16 years with *P vivax* mono-infection treated or not treated appropriately with tafenoquine in accordance with their G6PD status. The trial is registered with ClinicalTrials.gov, NCT05096702, and is completed.

**Findings:**

Of 6075 patients enrolled, 6026 (99·2%) had *P vivax* mono-infection, 2685 (44·6%) of whom were administered tafenoquine. G6PD status was identified in 2685 (100%) of 2685 patients treated with tafenoquine. The proportion of patients aged at least 16 years with *P vivax* mono-infection who were treated or not treated appropriately with tafenoquine in accordance with their G6PD status was 99·7% (95% CI 99·4–99·8; 4664/4680).

**Interpretation:**

Quantitative G6PD testing before tafenoquine administration was operationally feasible, with high adherence to the treatment algorithm, supporting deployment throughout the Brazilian health system.

**Funding:**

Brazilian Ministry of Health, Municipal and State Health Secretariats; Fiocruz; Medicines for Malaria Venture; Bill & Melinda Gates Foundation; Newcrest Mining; and the UK Government.

**Translation:**

For the Portuguese translation of the abstract see Supplementary Materials section.

## Introduction

Across the WHO region of the Americas, 71·5% of the 427 000 estimated malaria cases in 2021 were caused by *Plasmodium vivax*.^1^ *P vivax* infection is characterised by latent parasites in the liver (hypnozoites), which can reactivate, causing recurrent infection (relapses) weeks or months after the initial infection. This adaptation allows the parasite to evade control efforts and increases the malaria burden. Brazil is targeting malaria elimination by 2035, and *P vivax* relapse prevention is an essential element in achieving this goal.^2^

Complete treatment of *P vivax* malaria (radical cure) requires sterilisation of blood-stage parasites and hypnozoites using a schizonticide plus an 8-aminoquinoline, either primaquine or tafenoquine. Both 8-aminoquinolines can cause haemolysis in individuals with glucose-6-phosphate dehydrogenase (G6PD) deficiency. This X-linked enzymopathy is common in populations historically exposed to malaria, with an overall prevalence of 5·6% in the Brazilian Amazon.^3^ G6PD-deficient erythrocytes are sensitive to oxidative stress. The severity of triggered haemolysis depends on G6PD enzyme activity and the 8-aminoquinoline dose.^4^, ^5^, ^6^ Severe haemolysis clinically manifests as acute haemolytic anaemia, leading to hospitalisations and deaths.^7^

In Brazil, chloroquine plus 7-day primaquine (0·5 mg/kg per day) is standard for patients with *P vivax* malaria who are G6PD normal or intermediate, with artemisinin-based combination therapy plus 14-day primaquine (0·5 mg/kg per day) for recurrent cases.^8^, ^9^, ^10^ Patients who are G6PD deficient receive chloroquine plus once-weekly primaquine (0·75 mg/kg per week) for 8 weeks. G6PD testing before primaquine administration is recommended by WHO but is not implemented within the routine of the Brazilian health system.

**Research in context**

**Evidence before this study**

We searched PubMed for studies published from database inception until Nov 1, 2023, using the terms [tafenoquine] AND [vivax] FILTER [clinical trial], and found 29 results. Eight studies concerned the indication of *Plasmodium vivax* relapse prevention. Two major phase 3 clinical studies reported a significantly lower risk of recurrence after 6 months with chloroquine and tafenoquine (0·30 [95% CI 0·22–0·40]) compared with chloroquine and placebo (p<0·001), similar to that observed with chloroquine and primaquine versus chloroquine and placebo (0·26 [0·18–0·39]; p<0·001). Among patients with normal glucose-6-phosphate dehydrogenase (G6PD) enzyme activity, the decline in haemoglobin concentration following tafenoquine did not differ significantly from that following primaquine. The percentage of recurrence-free patients at 6 months was 67·0% (61·0–72·3) with tafenoquine (n=426) and 72·8% (65·6–78·8) with primaquine (n=214). An additional study done in Indonesian soldiers with *P vivax* malaria found that tafenoquine combined with dihydroartemisinin–piperaquine (n=50) had a 6-month relapse-free efficacy of 21% (11–34) compared with 11% (4–22) with dihydroartemisinin–piperaquine alone (n=50) and 52% (37–65) for primaquine plus dihydroartemisinin–piperaquine (n=50). Consequently, tafenoquine is approved for use with chloroquine and not dihydroartemisinin–piperaquine or other artemisinin-based combination therapies. Single-dose tafenoquine offers the potential for increased treatment adherence versus 7-day or 14-day primaquine. However, tafenoquine operational feasibility, including the need to establish G6PD status before treatment using a point-of-care quantitative G6PD test, has not been investigated under real-life conditions.

**Added value of this study**

This study examined the operational feasibility of the deployment of quantitative G6PD testing before 3-day chloroquine and either tafenoquine or primaquine treatment, using a revised *P vivax* treatment algorithm based on G6PD status, age, and pregnancy and breastfeeding status in two malaria-endemic regions in Brazil. The capacity to link malaria patient records to hospitalisation records allowed assessment of the risk of acute haemolytic anaemia.

**Implications of all the available evidence**

The implementation of the revised treatment algorithm for *P vivax* radical cure including quantitative G6PD testing and operational deployment of single-dose tafenoquine was feasible in the Brazilian context, with no concerning risk of drug-related acute haemolytic anaemia. This study supports the national use of this treatment algorithm for radical cure of *P vivax* malaria.

Tafenoquine is administered as a single oral 300 mg dose. In multicentre clinical trials in G6PD-normal patients, chloroquine plus tafenoquine reduced the 6-month incidence of *P vivax* malaria recurrence by 70% (95% CI 60–78) compared with chloroquine plus placebo,^11^ with no occurrences of drug-induced acute haemolytic anaemia.^12^ Tafenoquine must only be administered to patients with G6PD enzymatic activity at least 70% that of G6PD-normal patients and a quantitative G6PD test is required before treatment, as per label. Tafenoquine was registered by the Brazilian health regulatory authority (ANVISA) in October, 2019, and a quantitative point-of-care G6PD diagnostic has been operationally assessed in Brazil before primaquine administration, with good acceptability.^13^, ^14^

The Brazilian Ministry of Health approved preliminary implementation of tafenoquine in the Manaus and Porto Velho municipalities in February, 2021. The implementation evaluated real-life experience with a revised treatment algorithm for *P vivax* radical cure, incorporating quantitative G6PD testing and single-dose tafenoquine for patients with normal G6PD, aged at least 16 years (not pregnant or breastfeeding), before considering national roll-out. In Brazil, malaria patient data are routinely recorded in the malaria epidemiological surveillance system (SIVEP-Malaria). Because malaria is a mandatory notifiable disease with treatment provided only by the public health system and free of charge, SIVEP-Malaria captures data for all individuals who receive a malaria diagnostic test, including the test outcome and any treatment. The database thus includes all suspected and confirmed malaria cases in Brazil.^10^ Importantly, the data in SIVEP-Malaria can be linked to hospital records to assess adverse outcomes.^10^ Such analysis provides a timely mechanism for investigating the feasibility and effect of new tools.

This operational study used retrospective patient data from SIVEP-Malaria to investigate whether *P vivax* patients aged at least 16 years were treated or not treated appropriately with tafenoquine following quantitative G6PD testing. By linkage to hospitalisation and mortality data, the analysis also considered the safety of *P vivax* radical cure.

## Methods

### Study design

In this non-interventional, observational study, we used secondary data from the real-life implementation of tafenoquine in Manaus (Amazonas State) and Porto Velho (Rondônia State), Brazil. Implementation was supported by the federal, state, and municipality levels of the National Malaria Control Program (NMCP).

**Figure 1:**
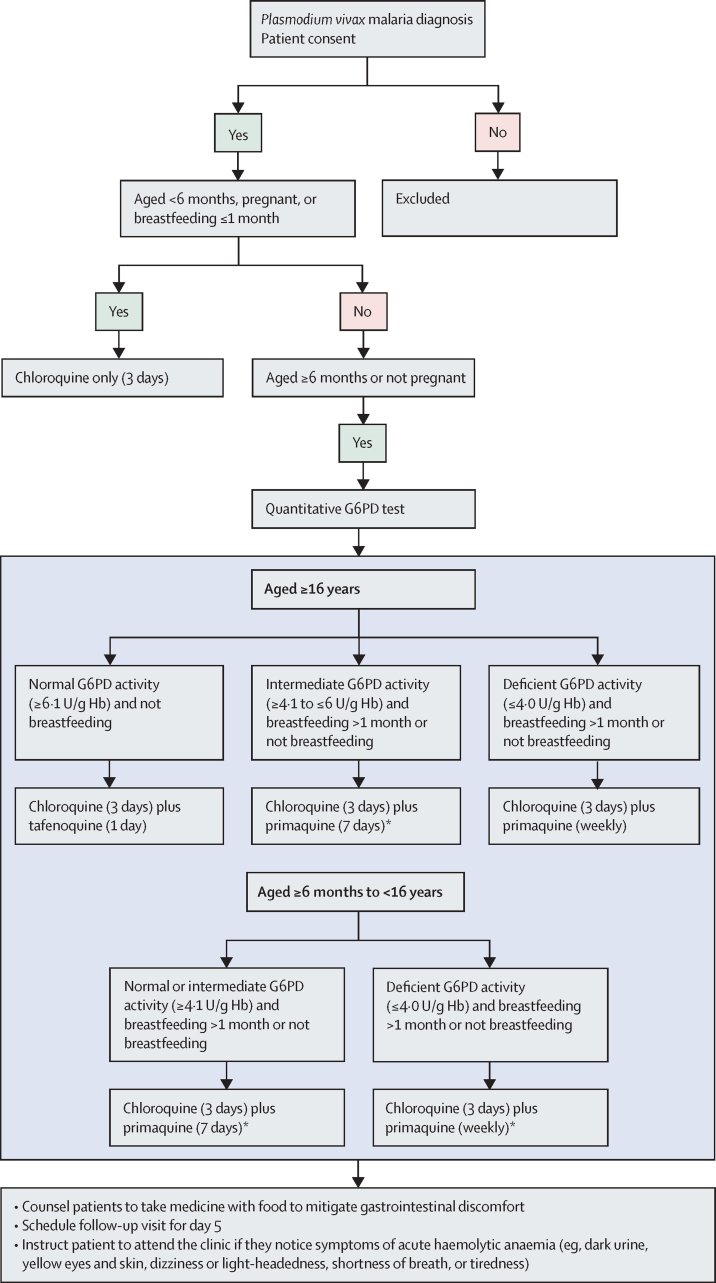
Treatment algorithm for *Plasmodium vivax* radical cure G6PD=glucose-6-phosphate dehydrogenase. *7-day primaquine is recommended for endemic regions but 14-day primaquine is also included in the Brazilian malaria treatment guidelines for recurrent *P vivax* and is available within the health system.

**Figure 2:**
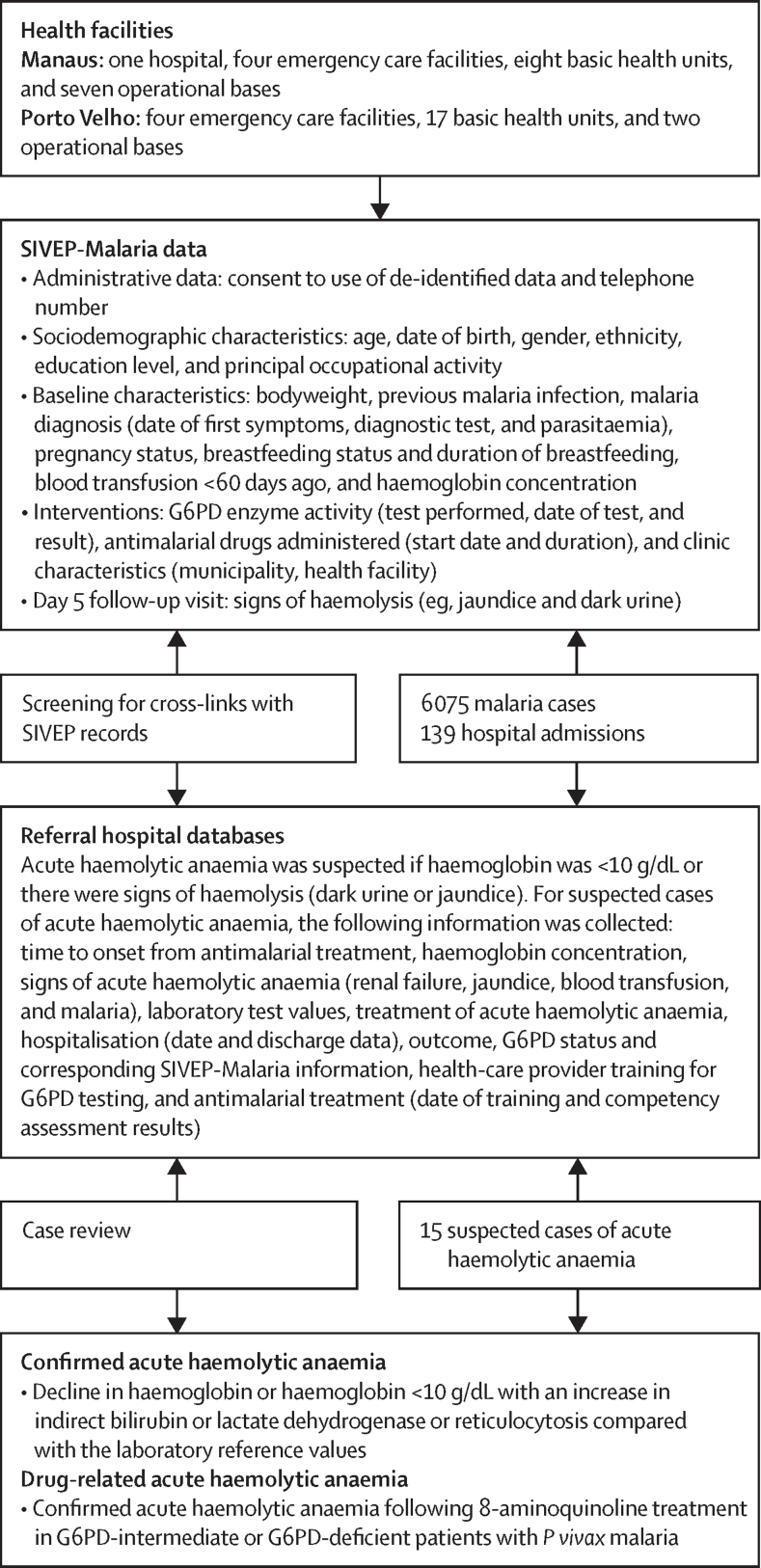
Data acquisition from SIVEP-Malaria and cross-linkage to hospital records to identify cases of acute haemolytic anaemia following malaria G6PD=glucose-6-phosphate dehydrogenase. SIVEP-Malaria=malaria epidemiological surveillance system.

Initially, staff at nine higher-level or medium-level health facilities (hospitals or emergency care) were trained on quantitative G6PD test procedures, tafenoquine administration, and the revised *P vivax* treatment algorithm (figure 1) using educational materials validated by the NMCP (to be published separately). Patient data were routinely collected by municipal health-care providers in SIVEP-Malaria, which had been updated to reflect the revised treatment algorithm and to prospectively obtain patient consent (figure 2). We did an interim analysis of 600 patient records to assess appropriate use at high-level facilities. Following review by an independent study oversight committee, training was extended to 34 lower-level health facilities (basic health units or operational bases). Six additional sites were trained but either had no malaria cases or no consenting patients. Municipal health authorities supplied G6PD test devices and strips and tafenoquine tablets to health-care facilities through the usual supply route for drugs and diagnostics. Primaquine and chloroquine were already available in the Brazilian public health system, provided free of charge following a malaria diagnostic test.

The study was done in compliance with Good Pharmacoepidemiology Practice guidelines, the Declaration of Helsinki (2013), and all existing local regulations. The protocol was reviewed by the Brazilian National Ethics in Research Committee (CAAE 16867319.6.0000.0008). For this retrospective analysis, patients were informed about the study and were explicitly asked for consent to use de-identified data, which were recorded in the SIVEP-Malaria form. The protocol and statistical analysis plan are provided in appendix 2 (pp 22–98).

### Participants

We reviewed records for all patients aged at least 6 months old (no maximum age limit) presenting to the 43 participating health facilities between Sept 9, 2021, and Aug 31, 2022 with *P vivax* malaria mono-infection or *P vivax* and *P falciparum* malaria mixed infection. Owing to an issue with the international supply of G6PD testing kits, implementation was suspended at some higher-level facilities in Porto Velho between mid-October and mid-November, 2021, and records for these patients were excluded. Malaria was confirmed through microscopy or rapid diagnostic test. To assess adherence to the treatment algorithm, we also included any patients younger than 6 months who were incorrectly treated with 8-aminoquinolines. We excluded non-consenting patient records. Follow-up records were analysed until Oct 30, 2022.

### Data management

The study used retrospective data collection. Municipal staff entered data into SIVEP-Malaria. Study staff had access to SIVEP-Malaria and hospital databases with due authorisation. Data were de-identified before analysis.

### Procedures

G6PD status was assessed using a point-of-care quantitative G6PD test (STANDARD G6PD test, SD Biosensor, Suwon-si, South Korea). Blood samples for analysis were obtained by fingerprick. The colorimetric test provides G6PD activity as a ratio to haemoglobin (U/g Hb) and haemoglobin concentration, with results available in approximately 2 min. Sensitivity and specificity are both 92–100%,^15^ with G6PD status defined as deficient (≤4·0 U/g Hb), intermediate (4·1–6·0 U/g Hb), or normal (≥6·1 U/g Hb), as per manufacturer's instructions.

Tests and treatments were prescribed by health-care staff in the higher-level units using their clinical judgement and treatment guidelines. In medium-level and lower-level units, health workers followed the treatment algorithm (figure 1). Drugs were chloroquine 150 mg tablets (Farmanguinhos, Fiocruz, Rio de Janeiro, Brazil), tafenoquine 150 mg tablets (GSK, Ware, UK), and primaquine 5 mg and 15 mg tablets (Farmanguinhos). All drugs were administered orally. Chloroquine 25 mg/kg was administered on days 1, 2, and 3; single-dose tafenoquine 300 mg on day 1; and primaquine 0·5 mg/kg per day for 7 days from day 1, or 0·75 mg/kg per week for 8 weeks from day 1. Patients were requested to return on day 5 for microscopic confirmation of blood-stage parasite clearance and to assess signs and symptoms of acute haemolytic anaemia (eg, jaundice and dark urine). The treatment algorithm did not include 14-day primaquine (0·5 mg/kg per day), because this regimen is recommended only for recurrent *P vivax* infection before 60 days after treatment (operational definition by the Ministry of Health), and not for new cases, which were the scope of our analyses in this study.

### Data acquisition and outcomes

We extracted de-identified individual patient data from SIVEP-Malaria (figure 2). The primary population included patients with *P vivax* mono-infection, excluding recurrent infection within 60 days (considered relapse according to NMCP guidelines). The primary endpoint was the percentage of patients with *P vivax* in the primary population aged 16 years or older and treated or not treated with tafenoquine in accordance with the appropriate G6PD activity calculated as:

$$\frac{\text{Number of patients with appropriate tafenoquine use or non}-\text{use}}{\left(\text{total number of patients receiving tafenoquine}+\text{appropriate non}-\text{use of tafenoquine}\right)}$$

Similarly, as a secondary endpoint, we calculated the percentage of patients with *P vivax* aged at least 6 months treated or not treated with primaquine in accordance with the appropriate level of G6PD activity. As an additional secondary endpoint, we described the characteristics of all patients treated with tafenoquine and primaquine.

Safety outcomes were the frequency of confirmed drug-induced acute haemolytic anaemia and hospitalisation due to drug-induced acute haemolytic anaemia in the safety population, including all patients who received tafenoquine or primaquine. Data were acquired by matching malaria-related hospital admissions to SIVEP-Malaria records using probabilistic linking using patient's name, date of birth, mother's name, and federation unit of malaria notification, with automatic verification using a probability threshold (>0·7) and individual linkage of discrepancies.^16^ Data matching was performed monthly between Sept 9, 2021, and Dec 30, 2022, using RStudio RecordLinkage (figure 2). Hospital records with potential acute haemolytic anaemia (ie, haemoglobin <10 g/dL or signs of haemolysis) prompted assessment of patient medical records and SIVEP-Malaria records. Investigators confirmed acute haemolytic anaemia post hoc on the basis of published criteria for G6PD-deficient haemolysis (figure 2).^17^ Relatedness to drug was assumed if the patient was G6PD deficient or intermediate, without a known cause of acute haemolytic anaemia, with onset consistent with drug administration.^17^ As a post-hoc analysis, we similarly assessed mortality following malaria through probabilistic linking of SIVEP-Malaria records with the mortality information system (SIM), which provides microdata for all deaths by age, sex, cause, and residence of the deceased, between Sept 9, 2021, and Feb 9, 2023.

### Statistical analysis

The statistical analysis plan was finalised before database lock. Based on historical *P vivax* and G6PD deficiency prevalence data, assuming the percentage of patients with *P vivax* aged at least 16 years who were treated or not treated appropriately with tafenoquine was 80%, the precision of a study including at least 4000 patients analysed for the primary outcome would be 1·2% (α=0·05). All outcomes were presented using descriptive statistics, with two-sided Clopper-Pearson 95% CIs where appropriate. Additional analysis populations were patients with mixed *P vivax* and *P falciparum* infection and those with recurrent *P vivax* mono-infection within 60 days of the initial infection (to be reported separately). Analyses were performed overall and by subgroups—ie, treatment, municipality, higher-level or medium-level versus lower-level health facility, sex, age group, ethnic group, G6PD status, and bodyweight group. All analyses were done using SAS (version 9.4).

### Role of the funding source

Medicines for Malaria Venture and the Brazilian Ministry of Health, Municipal and State Health Secretariats were involved in the protocol design, data analysis, and reporting. Other donors were not involved in the study design or conduct.

## Results

**Figure 3:**
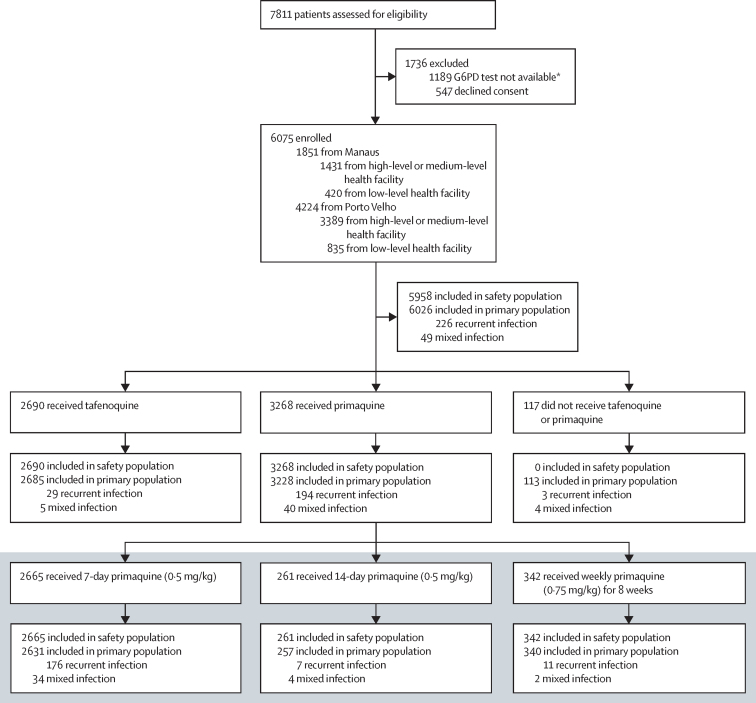
Study profile The primary analysis was done in the primary population who had *Plasmodium vivax* monotherapy and with the exclusion of malaria recurrences. Mixed infection was *P vivax* and *P falciparum*. G6PD=glucose-6-phosphate dehydrogenase. *An unanticipated temporary stockout of G6PD tests caused by international supply issues required the suspension of treatment algorithm implementation in some facilities in Porto Velho and withdrawal of tafenoquine. Patients treated during this time were excluded to maintain the integrity of the analysis.

Of 7811 patients assessed for eligibility, we enrolled 6075 (77·8%; figure 3). Most patients were from Porto Velho (4224 [69·5%]) and higher-level or medium-level health facilities (4820 [79·3%]; figure 3). The primary population included 6026 patients with *P vivax* mono-infection. The mean age of participants was 36·2 years (SD 16·3), 3914 (65·0%) were male, 2112 (35**·**0%) were female, and most were of mixed ethnicity (5081 [84**·**3%]; table 1; appendix 2 p 2; figure 3). We enrolled 49 (0·8%) pregnant women; none received 8-aminoquinoline treatment (table 1). There were no differences in patients presenting to higher-level or medium-level versus lower-level facilities (appendix 2 p 3), or by sex (appendix 2 p 4). Most patients (5903 [98**·**0%] of 6026) were diagnosed using microscopy and 5533 (93**·**7%) of 5905 evaluated patients had fewer than 10 000 parasites per mm^3^ based on semi-quantitative parasite counts (appendix 2 pp 5, 6).

**Table 1:** Key patient baseline demographic and clinical characteristics of the primary population

		**Tafenoquine (n=2685)**	**Primaquine**				**No tafenoquine or primaquine (n=113)**
			7-day (n=2631)	14-day (n=257)	Weekly (n=340)	All (n=3228)	
Municipality							
	Manaus	921 (34·3%)	707 (26·9%)	53 (20·6%)	113 (33·2%)	873 (27·0%)	46 (40·7%)
	Porto Velho	1764 (65·7%)	1924 (73·1%)	204 (79·4%)	227 (66·8%)	2355 (73·0%)	67 (59·3%)
Age, years		39·8 (14·0)	33·2 (17·7)	35·4 (16·4)	34·8 (16·6)	33·5 (17·5)	28·1 (12·6)
Sex							
	Male	1783 (66·4%)	1705 (64·8%)	170 (66·1%)	220 (64·7%)	2095 (64·9%)	36 (31·9%)
	Female	902 (33·6%)	926 (35·2%)	87 (33·9%)	120 (35·3%)	1133 (35·1%)	77 (68·1%)
Childbearing potential*		658 (72·9%)	506 (54·6%)	53 (60·9%)	78 (65·0%)	637 (56·2%)	67 (87·0%)
Breastfeeding†							
	Yes	6 (0·9%)	54 (10·7%)	2 (3·8%)	7 (9·0%)	63 (9·9%)	7 (10·4%)
	No	617 (93·8%)	416 (82·2%)	44 (83·0%)	67 (85·9%)	527 (82·7%)	54 (80·6%)
	Missing	35 (5·3%)	36 (7·1%)	7 (13·2%)	4 (5·1%)	47 (7·4%)	6 (9·0%)
Pregnant†							
	Yes	0	0	0	0	0	49 (73·1%)‡
	No	658 (100·0%)	506 (100·0%)	53 (100·0%)	78 (100·0%)	637 (100·0%)	20 (29·9%)
Ethnicity							
	Mixed	2275 (84·7%)	2208 (83·9%)	211 (82·1%)	292 (85·9%)	2711 (84·0%)	95 (84·1%)
	White	275 (10·2%)	293 (11·1%)	29 (11·3%)	35 (10·3%)	357 (11·1%)	13 (11·5%)
	Black	93 (3·5%)	88 (3·3%)	11 (4·3%)	8 (2·4%)	107 (3·3%)	2 (1·8%)
	Indigenous	25 (0·9%)	28 (1·1%)	5 (1·9%)	3 (0·9%)	36 (1·1%)	1 (0·9%)
	Asian	17 (0·6%)	14 (0·5%)	1 (0·4%)	2 (0·6%)	17 (0·5%)	2 (1·8%)

Data are n (%) or mean (SD). Additional baseline data are included in the appendix (p 2).

* Denominator for percentage is the number of female participants.

† Denominator for percentage is the number of female participants of childbearing potential (defined as age ≥16 to <50 years).

‡ Includes two patients younger than 16 years.

G6PD status was identified for 5712 (94·8%) of 6026 patients; 2685 (100%) of 2685 were treated with tafenoquine and 2954 (91·5%) of 3228 with primaquine (appendix 2 p 8). A greater proportion of patients aged at least 16 years (5141 [95·4%] of 5387) were tested than patients younger than 16 years (571 [89·4%] of 639; appendix 2 p 9). G6PD deficiency prevalence was 7·0% (263/3743) in males and 7·7% (151/1969) in females (appendix 2 p 10). We found a continuous distribution of G6PD activity levels (range 0·1–19·5 U/g Hb; appendix 2 p 10). For patients completing G6PD screening, 2669 (99·4%) of 2685 who were administered tafenoquine were G6PD normal, and 2520 (96·3%) of 2617 who were administered daily primaquine were either G6PD normal or intermediate (appendix 2 p 11).

For the primary outcome, 99·7% (95% CI 99·4–99·8; 4664/4680) of patients with *P vivax* mono-infection aged at least 16 years were treated or not treated with tafenoquine in accordance with the appropriate G6PD activity (table 2). Outcomes were consistent across health facility levels and between municipalities (appendix 2 p 12). Among all patients who received tafenoquine, 2623 (97·7% [97·0–98·2]) of 2685 were treated according to the treatment algorithm (table 3). Of 62 patients who were incorrectly treated, 41 (66·1%) were breastfeeding or with unknown breastfeeding status, 11 (17·7%) were G6PD intermediate, five (8·1%) were G6PD deficient, and five (8·1%) were younger than 16 years (table 3). Most women with unknown breastfeeding status were treated at the basic health-unit level, and all children younger than 16 years received tafenoquine at emergency care centres (table 3).

**Table 2:** Primary outcome: tafenoquine appropriate use and appropriate non-use in patients aged at least 16 years according to G6PD activity by facility level and type of facility and overall

		**Higher-level and medium-level facilities**			**Lower-level facilities**			**Overall (n=6026)**
		Hospitals (n=753)	Emergency care (n=4035)	All (n=4788)	Basic health unit (n=892)	Operational base (n=346)	All (n=1238)	
Patients aged ≥16 years		710	3630	4340	752	295	1047	5387
Total tafenoquine use		520 (73·2%)	1778 (49·0%)	2298 (52·9%)	270 (35·9%)	112 (38·0%)	382 (36·5%)	2680 (49·7%)
	Tafenoquine appropriate use	519 (73·1%)	1766 (48·7%)	2285 (52·6%)	267 (35·5%)	112 (38·0%)	379 (36·2%)	2664 (49·5%)
	Tafenoquine inappropriate use	1 (0·1%)	12 (0·3%)	13 (0·3%)	3 (0·4%)	0	3 (0·3%)	16 (0·3%)
Tafenoquine appropriate non-use		110 (15·5%)	1312 (36·1%)	1422 (32·8%)	404 (53·7%)	174 (59·0%)	578 (55·2%)	2000 (37·1%)
Tafenoquine appropriate use and appropriate non-use*		629/630	3078/3090	3707/3720	671/674	286/286	957/960	4664/4680
Tafenoquine appropriate use and appropriate non-use (95% CI)*		99·8% (99·1–100)	99·6% (99·3–99·8)	99·7% (99·4–99·8)	99·6% (98·7–99·9)	100% (98·7–100)	99·7% (99·1–99·9)	99·7% (99·4–99·8)

Data are n, n/N, or n (%), unless otherwise indicated.

* Calculated as: (tafenoquine appropriate use + tafenoquine appropriate non-use)/(total tafenoquine use + tafenoquine appropriate non-use).

**Table 3:** Characteristics of all patients receiving tafenoquine by facility level and type of facility and overall

		**Higher-level and medium-level facilities**			**Lower-level facilities**			**Overall (n=2685)**
		Hospitals (n=520)	Emergency care (n=1783)	All (n=2303)	Basic health unit (n=270)	Operational base (n=112)	All (n=382)	
Treatment algorithm*								
	Correct	508 (97·7% [96·0–98·8])	1750 (98·1% [97·4–98·7])	2258 (98·0% [97·4–98·6])	254 (94·1% [90·6–96·6])	111 (99·1% [95·1–100])	365 (95·5% [93·0–97·4])	2623 (97·7% [97·0–98·2])
	Incorrect	12 (2·3%)	33 (1·9%)	45 (2·0%)	16 (5·9%)	1 (0·9%)	17 (4·5%)	62 (2·3%)
G6PD activity†								
	Correct	519 (99·8% [98·9–100])	1771 (99·3% [98·8–99·7])	2290 (99·4% [99·0–99·7])	267 (98·9% [96·8–99·8])	112 (100% [96·8–100])	379 (99·2% [97·7–99·8])	2669 (99·4% [99·0–99·7])
	Incorrect	1 (0·2%)	12 (0·7%)	13 (0·6%)	3 (1·1%)	0	3 (0·8%)	16 (0·6%)
Age‡								
	Correct	520 (100% [99·3–100])	1778 (99·7% [99·3–99·9])	2298 (99·8% [99·5–99·9])	270 (100% [98·6–100])	112 (100% [96·8–100])	382 (100% [99·0–100])	2680 (99·8% [99·6–99·9])
	Incorrect	0	5 (0·3%)	5 (0·2%)	0	0	0	5 (0·2%)
Female participants of childbearing potential§		137	421	558	70	30	100	658
	Non-breastfeeding	126 (92·0% [86·1–95·9])	405 (96·2% [93·9–97·8])	531 (95·2% [93·0–96·8])	57 (81·4% [70·3–89·4])	29 (96·7% [82·8–99·9])	86 (86·0% [77·6–92·1])	617 (93·8% [91·6–95·5])
	Breastfeeding	1 (0·7%)	3 (0·7%)	4 (0·7%)	2 (2·9%)	0	2 (2·0%)	6 (0·9%)
	Breastfeeding unknown	10 (7·3%)	13 (3·1%)	23 (4·1%)	11 (15·7%)	1 (3·3%)	12 (12·0%)	35 (5·3%)
	Non-pregnant	137 (100% [97·3–100])	421 (100% [99·1–100])	558 (100% [99·3–100])	70 (100% [94·9–100])	30 (100% [88·4–100])	100 (100% [96·4–100])	658 (100% [99·4–100])
	Pregnant	0	0	0	0	0	0	0

Data are n, n (%), or n (% [95% CI]). G6PD=glucose-6-phosphate dehydrogenase.

* Based on normal G6PD activity (≥6·1 U/g Hb), in participants aged at least 16 years, non-breastfeeding, and non-pregnant.

† Based on normal G6PD activity (≥6·1 U/g Hb).

‡ Based on participants aged at least 16 years.

§ Based on female patients aged at least 16 years to younger than 50 years.

For daily primaquine (secondary outcome), 88·7% (95% CI 87·6–89·8; 2878/3244) of patients with *P vivax* aged at least 6 months were treated or not treated in accordance with the appropriate G6PD activity (appendix 2 pp 13, 14). The treatment algorithm was correctly applied for 2484 (86·0% [84·7–87·3]) of 2888 patients who received daily primaquine and for 307 (90·3% [86·6–93·2]) of 340 who received weekly primaquine (appendix 2 p 14). The most common deviation was incorrect G6PD (primaquine prescription not in agreement with G6PD status) for both daily (368 [91·1%] of 404) and weekly primaquine (29 [87·9%] of 33; appendix 2 p 14). Overall, of the 437 patents who were incorrectly administered primaquine, three (0·7%) were younger than 6 months and 47 (10·8%) had unknown breastfeeding status received primaquine (appendix 2 p 15).

Chloroquine was administered to 2682 (99·9%) of 2685 patients with tafenoquine and 2424 (92·1%) of 2631 with 7-day primaquine. Artemisinin-based combination therapy was given as rescue treatment for recurrences within 60 days of the primary infection to 133 (51·8%) of 257 patients treated with 14-day primaquine.

There were 49 (0·8%) of 6075 patients presenting with mixed infection (mean age 34·9 years [SD 16·4], 36 [73·5%] male, 13 [26·5%] female, and two [4·8%] with G6PD deficiency; appendix 2 pp 16, 17). All five patients who received tafenoquine were G6PD normal, and one (2·9%) of 34 patients who received 7-day primaquine was G6PD deficient (appendix 2 p 17). Eight (16·3%) of 49 patients received treatment for *P falciparum* within the previous 40 days (appendix 2 p 18).

Pre-treatment haemoglobin concentrations were similar across groups receiving tafenoquine or primaquine (appendix 2 p 19), with no differences by health-care facility level and municipality (appendix 2 p 20). Pre-treatment severe anaemia (<7 g/dL) was reported for 42 (0·7%) of 5712 patients: 32 (0·8%) of 3776 were G6PD normal, four (0·3%) of 1522 were G6PD intermediate, and six (1·4%) of 414 were G6PD deficient.

Day-5 follow-up was completed for 622 (23·2%) of 2685 patients following tafenoquine, with 56 (9·0%) of 622 self-reporting symptoms of acute haemolytic anaemia (appendix 2 p 21). Of 16 G6PD-deficient or G6PD-intermediate patients who were administered tafenoquine, three (18·8%) completed day-5 follow-up, and one G6PD-deficient patient had signs of acute haemolytic anaemia but was already hospitalised as a precaution. Of the nine patients with pre-treatment haemoglobin concentrations below 7 g/dL who were treated with tafenoquine, one returned on day 5 with acute haemolytic anaemia symptoms (G6PD normal). Day-5 follow-up was completed for 191 (6·6%) of 2888 patients receiving daily primaquine, with nine (4·7%) self-reporting symptoms of acute haemolytic anaemia (appendix 2 p 21). For 97 patients with G6PD deficiency treated with daily primaquine, one returned on day 5 but the acute haemolytic anaemia assessment was unknown. Of six patients with pre-treatment haemoglobin concentrations <7 g/dL treated with daily primaquine, one returned on day 5 with symptoms of acute haemolytic anaemia (G6PD normal). For patients who were administered weekly primaquine, 24 (7·1%) of 340 returned on day 5, with no symptoms of acute haemolytic anaemia (appendix 2 p 21).

Database linking of SIVEP-Malaria with the regional referral hospitals identified 139 malaria-related hospital admissions (39 [28·1%] treated with tafenoquine and 100 [71·9%] with primaquine). All were discharged following recovery. Assessment of the clinical and laboratory characteristics of these patients identified 15 suspected cases of acute haemolytic anaemia (three with tafenoquine and 12 with primaquine). Biochemical examinations and temporal information on drug administration allowed drug-related and malaria-related acute haemolytic anaemia to be distinguished. All three tafenoquine cases were G6PD normal, with acute haemolytic anaemia not considered drug related: one was associated with trauma from an accident exacerbated by malaria, one had malaria-associated haemolysis plus tafenoquine-related methaemoglobinaemia (16·5% methaemoglobin), and one had autoimmune haemolytic anaemia (positive Coombs test). Additionally, a G6PD-deficient female patient who was treated with tafenoquine had rhabdomyolysis and was discharged after full recovery. This patient also experienced a decline in haemoglobin (nadir 6·7 g/dL on day 5), without weakness, dizziness, myalgia, or dyspnoea, and did not require blood transfusion. The investigators considered rhabdomyolysis related to tafenoquine, and severe anaemia not drug related on consideration of biochemical findings. The details of the case were reported to ANVISA as a precaution.

Of the 12 patients with acute haemolytic anaemia treated with primaquine, drug-related acute haemolytic anaemia was suspected for seven (58·3%), including one G6PD-intermediate patient (onset day 3, haemoglobin 6·8 g/dL) and six G6PD-deficient patients (onset days 3–6, haemoglobin 4·3–8·1 g/dL). The remaining five (41·7%) cases were not considered primaquine related, with onset on day 0 or 1 (one G6PD normal and three G6PD deficient), or chronic bleeding (G6PD intermediate, onset day 9).

Database linking of SIVEP-Malaria and the SIM identified 13 deaths following a malaria diagnosis. None was considered drug related. Seven (53·8%) patients received tafenoquine, and causes of death were electric shock, cancer, COVID-19, hypertension, external cause (injury), and two with undetermined cause: one died at 329 days after treatment (G6PD status unknown) and one at 40 days after treatment (G6PD normal). Of the five (38·5%) patients prescribed primaquine, causes of death were severe malaria plus hypertension, sepsis, two cases of external cause, and one patient died before primaquine administration.

## Discussion

To exclude patients most at risk of acute haemolytic anaemia, G6PD testing is necessary before 8-aminoquinoline administration for *P vivax* radical cure. This study showed that deployment of a quantitative G6PD test within the Brazilian public health system promoted appropriate treatment or non-treatment for *P vivax* radical cure with tafenoquine in 99·7% (95% CI 99·4–99·8) of patients.

Adherence to G6PD testing before tafenoquine administration was 100% (2685/2685). Although most patients were treated at higher-level or medium-level facilities, 1238 (46·1%) patients were treated at lower-level facilities. This high level of adherence, maintained across the health system, might be because of the novelty of tafenoquine and the concurrent deployment with point-of-care quantitative G6PD testing, training, and educational materials. Overall, five G6PD-deficient and 11 G6PD-intermediate patients received tafenoquine, with one reporting symptoms of acute haemolytic anaemia at day 5, which was not considered to be drug related. Baseline haemoglobin concentration did not appear to affect risk of acute haemolytic anaemia. Thus, with high adherence to G6PD quantitative testing and the treatment algorithm across the health system, tafenoquine deployment was not associated with a concerning risk of acute haemolytic anaemia.

The lowest level of G6PD testing adherence was with 7-day primaquine (2377 [90·3%] of 2631 patients), perhaps because primaquine has been used for many years without G6PD testing in Brazil. In the current study, seven patients had primaquine-related acute haemolytic anaemia, six of whom were G6PD deficient. The consequences of inappropriate primaquine use in G6PD-deficient patients has been well described in Brazil, including life-threatening anaemia, acute renal failure, and substantial economic costs.^18^, ^19^ Previous studies indicate that follow-up on day 5 would discover most cases of primaquine-induced acute haemolytic anaemia.^18^ However, overall, there was poor adherence to day-5 follow-up, with only 837 (14·2%) of 5913 patients returning. Improving this rate is challenging, as patients and health-care workers might not associate signs and symptoms of acute haemolytic anaemia with drug administration and patients might be reluctant to return unless they have concerning symptoms.^13^

Although the overall operational performance of the treatment algorithm was high, some additional areas are flagged for improvement. Although not included in the treatment algorithm, 14-day primaquine was prescribed for 257 (4·3%) of 6026 patients, possibly because the regimen is familiar to prescribers, and recommended for recurrent *P vivax*. Weekly primaquine was prescribed incorrectly to just 29 (0·5%) of 5298 patients with normal or intermediate G6PD activity, whereas daily primaquine was administered incorrectly to 97 (23·4%) of 414 G6PD-deficient patients, so further work is needed to support appropriate primaquine administration. Although no pregnant women were administered an 8-aminoquinoline, breastfeeding status was not known for 88 (6·5%) of 1362 women of childbearing potential, requiring further emphasis in the routine of the NMCP. Logistically, the deployment of quantitative G6PD tests, tafenoquine, and primaquine through the normal channels of the Brazilian health service operated smoothly. There was a temporary stockout of G6PD tests for 1 month caused by a global supply issue. Although quickly resolved, stockout might be a consideration for health systems as more countries deploy these new tools and greater levels of advance stock might need to be purchased.

SIVEP-Malaria allows almost real-time observations of malaria management and any concerning deviations from treatment recommendations can be quickly identified and remedied. Thus, continuous assessment and improvement to support the high adherence levels observed in the initial deployment of the treatment algorithm is feasible in the wider health-care system.

The field performance of the G6PD quantitative test has been previously shown in Brazil.^13^, ^14^ Consistent with previous findings, a continuous range of G6PD activity was observed with no clear breakpoints for deficient, intermediate, or normal G6PD status, underlining the need for quantitative G6PD testing to correctly identify those at risk of acute haemolytic anaemia following tafenoquine or primaquine. The prevalence of G6PD deficiency (414 [7·2%] of 5712) was similar to that reported across the Amazon.^3^ Although this prevalence appears to be a small percentage, such individuals are at risk of serious adverse events at considerable cost to the health system.^7^, ^18^

Tafenoquine effectiveness in this study is being investigated in a companion exploratory analysis (to be published separately). Given the presence of chloroquine-resistant *P vivax* in the Brazilian Amazon,^16^ tafenoquine has significant, although slow, schizonticidal activity, and its use with chloroquine represents incidental combination therapy for uncomplicated *P vivax* malaria.^20^, ^21^

Despite the potential adherence benefits of single-dose tafenoquine,^22^ the challenges to deploying tafenoquine with quantitative G6PD testing have been emphasised.^23^ The findings of this study are not only positive for malaria elimination efforts in Brazil, but also show that implementation of these new tools is feasible within a public health system. However, there are some caveats in directly applying these findings elsewhere. First, the population at risk might be different; in Brazil malaria cases occur mainly in males aged at least 16 years, related to occupational risk.^24^, ^25^ Also, the A(−)^202A/376G^ and A(+)^376G^ G6PD variants are predominant in Brazil, both classified as mild.^3^, ^26^ Risk of acute haemolytic anaemia with more severe variants, such as Mahidol^487A^, requires verification. In Brazil, implementation was supported by the SIVEP-Malaria database, and similar information systems might not be available.

The major limitations of this study stem from its observational design, which was targeted at understanding the real-life scenario. We did not collect tolerability and adherence data, although it is usual to observe the first dose of medication with subsequent doses unsupervised. Most patients did not return for assessment of signs of acute haemolytic anaemia on day 5. Patients were made aware of symptoms of acute haemolytic anaemia and would presumably be more likely to return if they were concerned. We systematically assessed hospital records for cases of acute haemolytic anaemia, but data were analysed retrospectively and, although robust, probabilistic linking is imperfect. Linkage with mortality records indicated that no drug-related deaths occurred. Notably, the study populations for each treatment are different and no direct comparisons can be made between outcomes following tafenoquine and primaquine. However, the burden of primaquine-triggered acute haemolytic anaemia among patients with G6PD deficiency should not be neglected.

In conclusion, use of a revised treatment algorithm for *P vivax* radical cure with deployment of G6PD quantitative point-of-care testing before tafenoquine or primaquine administration was shown to be operationally feasible in Brazil. These findings supported implementation of the revised treatment algorithm as a national policy for *P vivax* malaria.

## Data sharing

De-identified participant data are available on reasonable request and with completion of a signed data access agreement from https://www.mmv.org/about-us/contact-us, referencing this publication. Data will be available for at least 5 years from publication of this study.

## Declaration of interests

SD, PGD, IB-F, and EJ are employed by Medicines for Malaria Venture. All other authors declare no competing interests.

## Supplementary Materials

Portuguese translation of the abstract
