# Supplementary material for: Operational feasibility of Plasmodium vivax radical cure with tafenoquine or primaquine following point-of-care, quantitative glucose-6-phosphate dehydrogenase testing in the Brazilian Amazon: a real-life retrospective analysis
Source: Lancet Glob Health. 2024 Feb 14;12(3):e467–77. doi: 10.1016/S2214-109X(23)00542-9 (PMC10882209; doi:10.1016/S2214-109X(23)00542-9)
Supplement: Portuguese translation of the abstract [file mmc1.pdf]

THE LANCET
Global Health

Supplementary appendix 1

This translation in Portuguese was submitted by the authors and we reproduce it as supplied. It has not been peer reviewed. *The Lancet*'s editorial processes have only been applied to the original in English, which should serve as reference for this manuscript.

Esta tradução em português foi submetida pelos autores e nós não fizemos quaisquer alterações. Esta versão não foi revista por pares. O processo editorial do The Lancet só foi aplicado à versão original em inglês, que deve servir como referência para este artigo.

Supplement to: Brito M, Rufatto R, Murta F, et al. Operational feasibility of *Plasmodium vivax* radical cure with tafenoquine or primaquine following point-of-care, quantitative glucose-6-phosphate dehydrogenase testing in the Brazilian Amazon: a real-life retrospective analysis. *Lancet Glob Health* 2024; **12:** e467–77.

RESUMO

Background Para alcançar a eliminação da malária, o Brasil deve implementar a cura radical de *Plasmodium vivax*. Nosso objetivo foi investigar a viabilidade operacional do teste quantitativo de glicose-6-fosfato desidrogenase (G6PD), seguido de tratamento com cloroquina mais tafenoquina ou primaquina.

Métodos Este estudo observacional não intervencionista foi realizado em 43 unidades de saúde de Manaus (Estado do Amazonas) e Porto Velho (Estado de Rondônia), implementando um novo algoritmo de tratamento de *P vivax,* incorporando um teste quantitativo de G6PD no local do diagnóstico, e uma dose única de tafenoquina (pacientes com G6PD-normal, com 16 anos ou mais, não grávida ou lactante) ou primaquina (pacientes com G6PD-intermediário/normal, com 6 meses ou mais, não grávida ou lactante com mais de 1 mês). Após o treinamento da implementação dos profissionais de saúde, compilamos retrospectivamente os registros de rotina de pacientes do sistema de vigilância epidemiológica da malária (SIVEP-Malária) para todos os pacientes com consentimento, igual ou superior a 6 meses de idade, com monoinfecção por malária por *P vivax* ou infecção mista por *P vivax*/*P falciparum,* confirmadas parasitologicamente, entre 9 de setembro de 2021 e 31 de agosto de 2022. O desfecho primário foi a proporção de pacientes com 16 anos de idade ou mais, com monoinfecção por *P vivax,* tratados ou não tratados adequadamente com tafenoquina de acordo com seu status de G6PD. O ensaio está registrado em ClinicalTrials.gov (NCT05096702).

Achados Dos 6075 pacientes incluídos, 6026 (99·2%) tinham monoinfecção por *P vivax*, sendo que 2685 (44·6%) destes receberam tafenoquina. O status de G6PD foi determinado para 2685 (100%) dos 2685 pacientes tratados com tafenoquina. A proporção de pacientes com pelo menos 16 anos de idade com monoinfecção por *P vivax* tratados ou não tratados adequadamente com tafenoquina, de acordo com seu status de G6PD, foi de 99·7% (IC 95% 99·4–99·8; 4664/4680).

Interpretação A realização de testes quantitativos de G6PD antes da administração de tafenoquina foi operacionalmente viável, com alta adesão ao algoritmo de tratamento, apoiando a implantação em todo o sistema de saúde brasileiro.

Financiamento Ministério da Saúde, Secretarias Municipais e Estaduais de Saúde, Fiocruz, Medicines for Malaria Venture, Bill & Melinda Gates Foundation, Newcrest Mining e Governo do Reino Unido.
