# Supplementary material for: Operational feasibility of Plasmodium vivax radical cure with tafenoquine or primaquine following point-of-care, quantitative glucose-6-phosphate dehydrogenase testing in the Brazilian Amazon: a real-life retrospective analysis
Source: Lancet Glob Health. 2024 Feb 14;12(3):e467–77. doi: 10.1016/S2214-109X(23)00542-9 (PMC10882209; doi:10.1016/S2214-109X(23)00542-9)
Supplement: Supplementary appendix 2 [file mmc2.pdf]

THE LANCET
Global Health

Supplementary appendix 2

This appendix formed part of the original submission and has been peer reviewed. We post it as supplied by the authors.

Supplement to: Brito M, Rufatto R, Murta F, et al. Operational feasibility of *Plasmodium vivax* radical cure with tafenoquine or primaquine following point-of-care, quantitative glucose-6-phosphate dehydrogenase testing in the Brazilian Amazon: a real-life retrospective analysis. *Lancet Glob Health* 2024; **12:** e467–77.

Supplementary materials

Operational feasibility of *Plasmodium vivax* radical cure with tafenoquine or primaquine following point-of-care quantitative G6PD testing in the Brazilian Amazon – a real-life retrospective analysis

Marcelo Brito, et al.

Table S1 Additional socio-demographic and baseline characteristics (primary population). 2

Table S2 Baseline socio-demographic and clinical characteristics of patients, by health facility level (primary population). 3

Table S3 Baseline socio-demographic and clinical characteristics of patients, by sex (primary population). 4

Table S4 Malaria history by facility level (primary population). 5

Table S5 Malaria history by treatment group overall and by municipality (primary population). 6

Table S6 Quantitative G6PD test results by treatment, overall and by municipality (primary population). 8

Table S7 Quantitative G6PD testing by age group. 9

Figure S1 Proportion of patients who were G6PD-deficient, G6PD-intermediate, or G6PD-normal, by sex (A). G6PD activity of patients by G6PD status (deficient, intermediate, normal), by sex (B). 10

Figure S2 Proportion of patients who were G6PD-deficient, G6PD-intermediate, or G6PD-normal, by treatment. 11

Table S8 Primary outcome by municipality: tafenoquine appropriate use and appropriate non-use in patients ≥16 years old according to G6PD activity by facility level and type of facility and overall (primary population). 12

Table S9 Daily primaquine (7-day or 14-day) appropriate use and appropriate non-use in patients ≥6 months old according to G6PD activity by facility level and type of facility and overall. 13

Table S10 Primaquine appropriate use and appropriate non-use according to G6PD activity in patients ≥6 months old by facility level and type of facility, and overall (primary population). 14

Table S11 Characteristics of all patients receiving primaquine and by facility level and type of facility and overall (primary population) 15

Table S12 Baseline socio-demographic and clinical characteristics of patients with mixed *P. vivax*/*P. falciparum* infections, by treatment group. 16

Table S13 G6PD testing and G6PD status of patients with mixed infections, by treatment group. 17

Table S14 Malaria history of patients with mixed infections, by facility level. 18

Table S15 Pre-treatment haemoglobin levels by treatment group (primary population). 19

Table S16 Haemoglobin levels, overall and by facility level and municipality (primary population). 20

Table S17 Day 5 follow-up outcomes (safety population). 21

Study protocol 22

Statistical analysis plan 63

Table S1 Additional socio-demographic and baseline characteristics (primary population).

Characteristic	TQ (n=2685)	PQ				No TQ or PQ (n=113)
		7-day (n=2631)	14-day (n=257)	Weekly (n=340)	All (n=3228)	
Age class, years						
Birth to <5	0	98 (3·7)	7 (2·7)	8 (2·4)	113 (3·5)	7 (6·2)
≥5 to <16	5 (0·2)	447 (17·0)	26 (10·1)	36 (10·6)	509 (15·8)	5 (4·4)
≥16 to <30	718 (26·7)	588 (22·3)	62 (24·1)	86 (25·3)	736 (22·8)	52 (46·0)
≥30 to <50	1283 (47·8)	967 (36·8)	110 (42·8)	144 (42·4)	1221 (37·8)	43 (38·1)
≥50 to <70	621 (23·1)	483 (18·4)	50 (19·5)	58 (17·1)	591 (18·3)	6 (5·3)
≥70	58 (2·2)	48 (1·8)	2 (0·8)	8 (2·4)	58 (1·8)	0
Weight class, kg						
≥5 to <50	56 (2·1)	403 (15·3)	18 (7·0)	34 (10·0)	455 (14·1)	14 (12·4)
≥50 to <70	870 (32·4)	833 (31·7)	79 (30·7)	103 (30·3)	1015 (31·4)	41 (36·3)
≥70 to <90	972 (36·2)	821 (31·2)	79 (30·7)	122 (35·9)	1022 (31·7)	30 (26·5)
≥90 to <120	380 (14·2)	222 (8·4)	32 (12·5)	38 (11·2)	292 (9·0)	7 (6·2)
≥120	37 (1·4)	23 (0·9)	4 (1·6)	2 (0·6)	29 (0·9)	0
Missing	370 (13·8)	329 (12·5)	45 (17·5)	41 (12·1)	415 (12·9)	21 (18·6)
Education						
Illiterate	44 (1·6)	59 (2·2)	2 (0·8)	11 (3·2)	72 (2·2)	2 (1·8)
1st to 5th year	340 (12·7)	420 (16·0)	31 (12·1)	52 (15·3)	503 (15·6)	6 (5·3)
5th year	91 (3·4)	122 (4·6)	14 (5·4)	18 (5·3)	154 (4·8)	5 (4·4)
6th-9th year	402 (15·0)	506 (19·2)	44 (17·1)	59 (17·4)	609 (18·9)	19 (16·8)
Complete basic education	384 (14·3)	210 (8·0)	30 (11·7)	41 (12·1)	281 (8·7)	11 (9·7)
Incomplete high school	432 (16·1)	335 (12·7)	42 (16·3)	48 (14·1)	425 (13·2)	24 (21·2)
Complete high school	759 (28·3)	690 (26·2)	64 (24·9)	83 (24·4)	837 (25·9)	32 (28·3)
Incomplete college	66 (2·5)	45 (1·7)	10 (3·9)	4 (1·2)	59 (1·8)	1 (0·9)
Complete college	151 (5·6)	109 (4·1)	12 (4·7)	11 (3·2)	132 (4·1)	6 (5·3)
Not applicable	0	111 (4·2)	7 (2·7)	9 (2·6)	127 (3·9)	6 (5·3)
Missing	16 (0·6)	24 (0·9)	1 (0·4)	4 (1·2)	29 (0·9)	1 (0·9)
Occupation						
Agriculture	154 (5·7)	207 (7·9)	21 (8·2)	25 (7·4)	253 (7·8)	7 (6·2)
Livestock	2 (0·1)	8 (0·3)	1 (0·4)	3 (0·9)	12 (0·4)	0
Household	400 (14·9)	618 (23·5)	77 (30·0)	75 (22·1)	770 (23·9)	32 (28·3)
Tourism	116 (4·3)	143 (5·4)	13 (5·1)	8 (2·4)	164 (5·1)	4 (3·5)
Gold digging	32 (1·2)	22 (0·8)	1 (0·4)	4 (1·2)	27 (0·8)	2 (1·8)
Farming	36 (1·3)	54 (2·1)	2 (0·8)	8 (2·4)	64 (2·0)	1 (0·9)
Hunting/fishing	16 (0·6)	25 (1·0)	2 (0·8)	6 (1·8)	33 (1·0)	1 (0·9)
Construction	4 (0·1)	3 (0·1)	1 (0·4)	1 (0·3)	5 (0·2)	0
Mining	3 (0·1)	3 (0·1)	1 (0·4)	0	4 (0·1)	1 (0·9)
Traveller	39 (1·5)	36 (1·4)	2 (0·8)	3 (0·9)	41 (1·3)	0
Other	1789 (66·6)	1456 (55·3)	128 (49·8)	192 (56·5)	1776 (55·0)	61 (54·0)
Missing	94 (3·5)	56 (2·1)	8 (3·1)	15 (4·4)	79 (2·4)	4 (3·5)

Data are n (%). Key socio-democratic and baseline data are included in table 1 in the main paper.
TQ, tafenoquine; PQ, primaquine.

Table S2 Baseline socio-demographic and clinical characteristics of patients, by health facility level (primary population).

Characteristic	Higher/medium-level facilities			Lower-level facilities			Overall (n=6026)
	Hospital (n=753)	Emergency care (n=4035)	All (n=4788)	Basic health unit (n=892)	Operational base (n=346)	All (n=1238)	
Municipality							
Manaus	753 (100)	667 (16·5)	1420 (29·7)	117 (13·1)	303 (87·6)	420 (33·9)	1840 (30·5)
Porto Velho	0	3368 (83·5)	3368 (70·3)	775 (86·9)	43 (12·4)	818 (66·1)	4186 (69·5)
Age, years	38·0 (14·9)	37·0 (16·3)	37·2 (16·1)	32·5 (16·7)	32·8 (16·1)	32·6 (16·5)	36·2 (16·3)
Age class, years							
Birth to <5	14 (1·9)	65 (1·6)	79 (1·6)	30 (3·4)	11 (3·2)	41 (3·3)	120 (2·0)
≥5 to <16	29 (3·9)	340 (8·4)	369 (7·7)	110 (12·3)	40 (11·6)	150 (12·1)	519 (8·6)
≥16 to <30	187 (24·8)	953 (23·6)	1140 (23·8)	273 (30·6)	93 (26·9)	366 (29·6)	1506 (25·0)
≥30 to <50	350 (46·5)	1730 (42·9)	2080 (43·4)	318 (35·7)	149 (43·1)	467 (37·7)	2547 (42·3)
≥50 to <70	164 (21·8)	859 (21·3)	1023 (21·4)	145 (16·3)	50 (14·5)	195 (15·8)	1218 (20·2)
≥70	9 (1·2)	88 (2·2)	97 (2·0)	16 (1·8)	3 (0·9)	19 (1·5)	116 (1·9)
Sex							
Male	472 (62·7)	2638 (65·4)	3110 (65·0)	571 (64·0)	233 (67·3)	804 (64·9)	3914 (65·0)
Female	281 (37·3)	1397 (34·6)	1678 (35·0)	321 (36·0)	113 (32·7)	434 (35·1)	2112 (35·0)
Childbearing potential[a]	192 (68·3)	888 (63·6)	1080 (64·4)	204 (63·6)	78 (69·0)	282 (65·0)	1362 (64·5)
Breastfeeding[b]							
Yes	10 (5·2)	45 (5·1)	55 (5·1)	14 (6·9)	7 (9·0)	21 (7·4)	76 (5·6)
No	167 (87·0)	806 (90·8)	973 (90·1)	155 (76·0)	70 (89·7)	225 (79·8)	1198 (88·0)
Missing	15 (7·8)	37 (4·2)	52 (4·8)	35 (17·2)	1 (1·3)	36 (12·8)	88 (6·5)
Pregnant[b]							
Yes	11 (5·7)	25 (2·8)	36 (3·3)	7 (3·4)	4 (5·1)	11 (3·9)	47 (3·5)
No	181 (94·3)	863 (97·2)	1044 (96·7)	197 (96·6)	74 (94·9)	271 (96·1)	1315 (96·5)
Weight class, kg							
≥5 to <50	31 (4·1)	317 (7·9)	348 (7·3)	119 (13·3)	58 (16·8)	177 (14·3)	525 (8·7)
≥50 to <70	248 (32·9)	1196 (29·6)	1444 (30·2)	362 (40·6)	120 (34·7)	482 (38·9)	1926 (32·0)
≥70 to <90	255 (33·9)	1354 (33·6)	1609 (33·6)	279 (31·3)	136 (39·3)	415 (33·5)	2024 (33·6)
≥90 to <120	116 (15·4)	475 (11·8)	591 (12·3)	63 (7·1)	25 (7·2)	88 (7·1)	679 (11·3)
≥120	11 (1·5)	45 (1·1)	56 (1·2)	6 (0·7)	4 (1·2)	10 (0·8)	66 (1·1)
Missing	92 (12·2)	648 (16·1)	740 (15·5)	63 (7·1)	3 (0·9)	66 (5·3)	806 (13·4)
Ethnicity							
Mixed	713 (94·7)	3310 (82·0)	4023 (84·0)	759 (85·1)	299 (86·4)	1058 (85·5)	5081 (84·3)
White	13 (1·7)	545 (13·5)	558 (11·7)	67 (7·5)	20 (5·8)	87 (7·0)	645 (10·7)
Black	10 (1·3)	143 (3·5)	153 (3·2)	44 (4·9)	5 (1·4)	49 (4·0)	202 (3·4)
Indigenous	5 (0·7)	30 (0·7)	35 (0·7)	21 (2·4)	6 (1·7)	27 (2·2)	62 (1·0)
Asian	12 (1·6)	7 (0·2)	19 (0·4)	1 (0·1)	16 (4·6)	17 (1·4)	36 (0·6)
Education							
Illiterate	0	78 (1·9)	78 (1·6)	35 (3·9)	5 (1·4)	40 (3·2)	118 (2·0)
1st to 5th year	24 (3·2)	542 (13·4)	566 (11·8)	229 (25·7)	54 (15·6)	283 (22·9)	849 (14·1)
5th year	26 (3·5)	135 (3·3)	161 (3·4)	63 (7·1)	26 (7·5)	89 (7·2)	250 (4·1)
6th-9th year	72 (9·6)	689 (17·1)	761 (15·9)	193 (21·6)	76 (22·0)	269 (21·7)	1030 (17·1)
Complete basic education	305 (40·5)	268 (6·6)	573 (12·0)	67 (7·5)	36 (10·4)	103 (8·3)	676 (11·2)
Incomplete high school	147 (19·5)	591 (14·6)	738 (15·4)	95 (10·7)	48 (13·9)	143 (11·6)	881 (14·6)
Complete high school	129 (17·1)	1271 (31·5)	1400 (29·2)	149 (16·7)	79 (22·8)	228 (18·4)	1628 (27·0)
Incomplete college	25 (3·3)	92 (2·3)	117 (2·4)	6 (0·7)	3 (0·9)	9 (0·7)	126 (2·1)
Complete college	6 (0·8)	269 (6·7)	275 (5·7)	11 (1·2)	3 (0·9)	14 (1·1)	289 (4·8)
Not applicable	15 (2·0)	73 (1·8)	88 (1·8)	29 (3·3)	16 (4·6)	45 (3·6)	133 (2·2)
Missing	4 (0·5)	27 (0·7)	31 (0·6)	15 (1·7)	0	15 (1·2)	46 (0·8)
Occupation							
Agriculture	10 (1·3)	240 (5·9)	250 (5·2)	120 (13·5)	44 (12·7)	164 (13·2)	414 (6·9)
Livestock	2 (0·3)	7 (0·2)	9 (0·2)	3 (0·3)	2 (0·6)	5 (0·4)	14 (0·2)
Household	60 (8·0)	641 (15·9)	701 (14·6)	392 (43·9)	109 (31·5)	501 (40·5)	1202 (19·9)
Tourism	2 (0·3)	271 (6·7)	273 (5·7)	6 (0·7)	5 (1·4)	11 (0·9)	284 (4·7)
Gold digging	14 (1·9)	36 (0·9)	50 (1·0)	6 (0·7)	5 (1·4)	11 (0·9)	61 (1·0)
Farming	0	12 (0·3)	12 (0·3)	87 (9·8)	2 (0·6)	89 (7·2)	101 (1·7)
Hunting/fishing	0	30 (0·7)	30 (0·6)	19 (2·1)	1 (0·3)	20 (1·6)	50 (0·8)
Construction	0	5 (0·1)	5 (0·1)	4 (0·4)	0	4 (0·3)	9 (0·1)
Mining	0	6 (0·1)	6 (0·1)	2 (0·2)	0	2 (0·2)	8 (0·1)
Traveller	6 (0·8)	54 (1·3)	60 (1·3)	8 (0·9)	12 (3·5)	20 (1·6)	80 (1·3)
Other	575 (76·4)	2660 (65·9)	3235 (67·6)	225 (25·2)	166 (48·0)	391 (31·6)	3626 (60·2)
Missing	84 (11·2)	73 (1·8)	157 (3·3)	20 (2·2)	0	20 (1·6)	177 (2·9)

Data are n (%) and mean (standard deviation).
[a] Denominator for % is the number of females.
[b] Denominator for % is the number of females of childbearing potential (defined as age ≥16 to <50 years).

Table S3 Baseline socio-demographic and clinical characteristics of patients, by sex (primary population).

Characteristic	Male (n=3914)	Female (n=2112)
Age, years	36·1 (15·8)	36·4 (17·1)
Age class, years		
Birth to <5	77 (2·0)	43 (2·0)
≥5 to <16	309 (7·9)	210 (9·9)
≥16 to <30	978 (25·0)	528 (25·0)
≥30 to <50	1713 (43·8)	834 (39·5)
≥50 to <70	770 (19·7)	448 (21·2)
≥70	67 (1·7)	49 (2·3)
Weight class, kg		
≥5 to <50	280 (7·2)	245 (11·6)
≥50 to <70	1094 (28·0)	832 (39·4)
≥70 to <90	1462 (37·4)	562 (26·6)
≥90 to <120	518 (13·2)	161 (7·6)
≥120	58 (1·5)	8 (0·4)
Missing	502 (12·8)	304 (14·4)
Ethnicity		
Mixed	3306 (84·5)	1775 (84·0)
White	411 (10·5)	234 (11·1)
Black	146 (3·7)	56 (2·7)
Indigenous	30 (0·8)	32 (1·5)
Asian	21 (0·5)	15 (0·7)
Education		
Illiterate	80 (2·0)	38 (1·8)
1st to 5th year	595 (15·2)	254 (12·0)
5th year	171 (4·4)	79 (3·7)
6th-9th year	700 (17·9)	330 (15·6)
Complete basic education	438 (11·2)	238 (11·3)
Incomplete high school	558 (14·3)	323 (15·3)
Complete high school	1016 (26·0)	612 (29·0)
Incomplete college	84 (2·1)	42 (2·0)
Complete college	155 (4·0)	134 (6·3)
Not applicable	89 (2·3)	44 (2·1)
Missing	28 (0·7)	18 (0·9)
Occupation		
Agriculture	288 (7·4)	126 (6·0)
Livestock	12 (0·3)	2 (0·1)
Household	616 (15·7)	586 (27·7)
Tourism	174 (4·4)	110 (5·2)
Gold digging	39 (1·0)	22 (1·0)
Farming	72 (1·8)	29 (1·4)
Hunting/fishing	38 (1·0)	12 (0·6)
Construction	9 (0·2)	0
Mining	7 (0·2)	1 (0·0)
Traveller	58 (1·5)	22 (1·0)
Other	2490 (63·6)	1136 (53·8)
Missing	111 (2·8)	66 (3·1)

Data are n (%) and mean (standard deviation).

Table S4 Malaria history by facility level (primary population).

Characteristic	Higher/medium-level facilities			Lower-level facilities			Overall (n=6026)
	Hospital (n=753)	Emergency care (n=4035)	All (n=4788)	Basic health unit (n=892)	Operational base (n=346)	All (n=1238)	
Diagnostic test							
Thick blood smear	731 (97·1)	3979 (98·6)	4710 (98·4)	847 (95·0)	346 (100)	1193 (96·4)	5903 (98·0)
Rapid diagnostic test	22 (2·9)	21 (0·5)	43 (0·9)	44 (4·9)	0	44 (3·6)	87 (1·4)
Polymerase chain reaction	0	35 (0·9)	35 (0·7)	1 (0·1)	0	1 (0·1)	36 (0·6)
Parasitaemia							
n (%)	0	182 (4·5)	182 (3·8)	2 (0·2)	44 (12·7)	46 (3·7)	228 (3·8)
Mean (SD), per mm^3	–	12·5 (8·0)	12·5 (8·0)	30·0 (0·0)	14·1 (9·2)	14·8 (9·6)	13·0 (8·4)
Semi-quantitative parasitaemia[a]							
< half cross	72 (9·6)	410 (10·2)	482 (10·1)	168 (18·8)	78 (22·5)	246 (19·9)	728 (12·1)
Half cross	65 (8·6)	243 (6·0)	308 (6·4)	90 (10·1)	46 (13·3)	136 (11·0)	444 (7·4)
1 cross	92 (12·2)	665 (16·5)	757 (15·8)	200 (22·4)	82 (23·7)	282 (22·8)	1039 (17·2)
2 crosses	490 (65·1)	2358 (58·4)	2848 (59·5)	341 (38·2)	133 (38·4)	474 (38·3)	3322 (55·1)
3 crosses	12 (1·6)	299 (7·4)	311 (6·5)	48 (5·4)	7 (2·0)	55 (4·4)	366 (6·1)
4 crosses	0	5 (0·1)	5 (0·1)	1 (0·1)	0	1 (0·1)	6 (0·1)
Missing	22 (2·9)	55 (1·4)	77 (1·6)	44 (4·9)	0	44 (3·6)	121 (2·0)
Blood transfusion in last 60 days							
Yes	3 (0·4)	15 (0·4)	18 (0·4)	4 (0·4)	0	4 (0·3)	22 (0·4)
No	749 (99·5)	3683 (91·3)	4432 (92·6)	870 (97·5)	331 (95·7)	1201 (97·0)	5633 (93·5)
Missing	1 (0·1)	337 (8·4)	338 (7·1)	18 (2·0)	15 (4·3)	33 (2·7)	371 (6·2)
P· falciparum in the last 40 days							
Yes	21 (2·8)	32 (0·8)	53 (1·1)	7 (0·8)	2 (0·6)	9 (0·7)	62 (1·0)
No	732 (97·2)	4003 (99·2)	4735 (98·9)	885 (99·2)	344 (99·4)	1229 (99·3)	5964 (99·0)

Data are n (%) or mean (standard deviation).
[a] Half-cross (200–300 parasites/mm^3); 1 cross (301–500 parasites/mm^3); 2 crosses (501–10,000 parasites/mm^3); 3crosses (10,001–100,000 parasites/mm^3); and 4 crosses (>100,001 parasites/mm^3).

Table S5 Malaria history by treatment group overall and by municipality (primary population).

Overall (n=6026)	TQ (n=2685)	PQ				No TQ or PQ (n=113)
		7-day (n=2631)	14-day (n=257)	Weekly (n=340)	All (n=3228)	
Diagnostic test						
Thick blood smear	2641 (98·4)	2566 (97·5)	254 (98·8)	331 (97·4)	3151 (97·6)	111 (98·2)
Rapid diagnostic test	24 (0·9)	51 (1·9)	3 (1·2)	7 (2·1)	61 (1·9)	2 (1·8)
Polymerase chain reaction	20 (0·7)	14 (0·5)	0	2 (0·6)	16 (0·5)	0
Parasitaemia						
N	91 (3·4)	104 (4·0)	1 (0·4)	25 (7·4)	130 (4·0)	7 (6·2)
Mean (SD), per mm^3	12·5 (8·7)	13·3 (8·3)	17·0	12·4 (8·4)	13·2 (8·2)	14·7 (8·0)
Semi-quantitative parasitaemia[a]						
< half cross	285 (10·6)	344 (13·1)	20 (7·8)	60 (17·6)	424 (13·1)	19 (16·8)
Half cross	205 (7·6)	190 (7·2)	12 (4·7)	25 (7·4)	227 (7·0)	12 (10·6)
1 cross	441 (16·4)	472 (17·9)	49 (19·1)	62 (18·2)	583 (18·1)	15 (13·3)
2 crosses	1531 (57·0)	1406 (53·4)	155 (60·3)	171 (50·3)	1732 (53·7)	59 (52·2)
3 crosses	176 (6·6)	156 (5·9)	17 (6·6)	13 (3·8)	186 (5·8)	4 (3·5)
4 crosses	3 (0·1)	0	1 (0·4)	0	1 (0·0)	2 (1·8)
Missing	44 (1·6)	63 (2·4)	3 (1·2)	9 (2·6)	75 (2·3)	2 (1·8)
Blood transfusion in last 60 days						
Yes	7 (0·3)	13 (0·5)	1 (0·4)	1 (0·3)	15 (0·5)	0
No	2569 (95·7)	2410 (91·6)	233 (90·7)	318 (93·5)	2961 (91·7)	103 (91·2)
Missing	109 (4·1)	208 (7·9)	23 (8·9)	21 (6·2)	252 (7·8)	10 (8·8)
P. falciparum in the last 40 days						
Yes	23 (0·9)	26 (1·0)	9 (3·5)	3 (0·9)	38 (1·2)	1 (0·9)
No	2662 (99·1)	2605 (99·0)	248 (96·5)	337 (99·1)	3190 (98·8)	112 (99·1)
**Manaus (n=1840)**	**TQ (n=921)**	**PQ**				**No TQ or PQ (n=46)**
		**7-day (n=707)**	**14-day (n=53)**	**Weekly (n=113)**	**All (n=873)**	
Diagnostic test						
Thick blood smear	912 (99·0)	696 (98·4)	53 (100)	113 (100)	862 (98·7)	44 (95·7)
Rapid diagnostic test	9 (1·0)	11 (1·6)	0	0	11 (1·3)	2 (4·3)
Polymerase chain reaction	0	0	0	0	0	0
Parasitaemia						
N	14 (1·5)	14 (2·0)	0	16 (14·2)	30 (3·4)	2 (4·3)
Mean (SD), per mm^3	15·1 (10·1)	14·1 (9·2)	–	12·2 (9·1)	13·1 (9·0)	12·5 (10·6)
Semi-quantitative parasitaemia[a]						
< half cross	97 (10·5)	89 (12·6)	5 (9·4)	28 (24·8)	122 (14·0)	8 (17·4)
Half cross	80 (8·7)	59 (8·3)	2 (3·8)	12 (10·6)	73 (8·4)	7 (15·2)
1 cross	126 (13·7)	103 (14·6)	8 (15·1)	12 (10·6)	123 (14·1)	3 (6·5)
2 crosses	575 (62·4)	402 (56·9)	36 (67·9)	58 (51·3)	496 (56·8)	25 (54·3)
3 crosses	33 (3·6)	43 (6·1)	2 (3·8)	3 (2·7)	48 (5·5)	1 (2·2)
4 crosses	1 (0·1)	0	0	0	0	0
Missing	9 (1·0)	11 (1·6)	0	0	11 (1·3)	2 (4·3)
Blood transfusion in last 60 days						
Yes	1 (0·1)	3 (0·4)	0	0	3 (0·3)	0
No	854 (92·7)	560 (79·2)	46 (86·8)	97 (85·8)	703 (80·5)	40 (87·0)
Missing	66 (7·2)	144 (20·4)	7 (13·2)	16 (14·2)	167 (19·1)	6 (13·0)
P. falciparum in the last 40 days						
Yes	15 (1·6)	9 (1·3)	1 (1·9)	1 (0·9)	11 (1·3)	0
No	906 (98·4)	698 (98·7)	52 (98·1)	112 (99·1)	862 (98·7)	46 (100)
**Porto Velho (n=4186)**	**TQ (n=1764)**	**PQ**				**No TQ or PQ (n=67)**
		**7-day (n=1924)**	**14-day (n=204)**	**Weekly (n=227)**	**All (n=2355)**	
Diagnostic test						
Thick blood smear	1729 (98·0)	1870 (97·2)	201 (98·5)	218 (96·0)	2289 (97·2)	67 (100)
Rapid diagnostic test	15 (0·9)	40 (2·1)	3 (1·5)	7 (3·1)	50 (2·1)	0
Polymerase chain reaction	20 (1·1)	14 (0·7)	0	2 (0·9)	16 (0·7)	0
Parasitaemia						
N	77 (4·4)	90 (4·7)	1 (0·5)	9 (4·0)	100 (4·2)	5 (7·5)
Mean (SD), per mm^3	12·0 (8·4)	13·2 (8·2)	17·0	12·7 (7·3)	13·2 (8·0)	15·6 (8·0)
Semi-quantitative parasitaemia[a]						
< half cross	188 (10·7)	255 (13·3)	15 (7·4)	32 (14·1)	302 (12·8)	11 (16·4)
Half cross	125 (7·1)	131 (6·8)	10 (4·9)	13 (5·7)	154 (6·5)	5 (7·5)
1 cross	315 (17·9)	369 (19·2)	41 (20·1)	50 (22·0)	460 (19·5)	12 (17·9)
2 crosses	956 (54·2)	1004 (52·2)	119 (58·3)	113 (49·8)	1236 (52·5)	34 (50·7)
3 crosses	143 (8·1)	113 (5·9)	15 (7·4)	10 (4·4)	138 (5·9)	3 (4·5)
4 crosses	2 (0·1)	0	1 (0·5)	0	1 (0·0)	2 (3·0)
Missing	35 (2·0)	52 (2·7)	3 (1·5)	9 (4·0)	64 (2·7)	0
Blood transfusion in last 60 days						
Yes	6 (0·3)	10 (0·5)	1 (0·5)	1 (0·4)	12 (0·5)	0
No	1715 (97·2)	1850 (96·2)	187 (91·7)	221 (97·4)	2258 (95·9)	63 (94·0)
Missing	43 (2·4)	64 (3·3)	16 (7·8)	5 (2·2)	85 (3·6)	4 (6·0)
P. falciparum in the last 40 days						
Yes	8 (0·5)	17 (0·9)	8 (3·9)	2 (0·9)	27 (1·1)	1 (1·5)
No	1756 (99·5)	1907 (99·1)	196 (96·1)	225 (99·1)	2328 (98·9)	66 (98·5)

Data are n (%) or mean (standard deviation). TQ, tafenoquine, PQ, primaquine.
[a] Half-cross (200–300 parasites/mm^3); 1 cross (301–500 parasites/mm^3); 2 crosses (501–10,000 parasites/mm^3); 3crosses (10,001–100,000 parasites/mm^3); and 4 crosses (>100,001 parasites/mm^3).

Table S6 Quantitative G6PD test results by treatment, overall and by municipality (primary population).

Overall (n=6026)	TQ (n=2685)	PQ 7-day (n=2631)	PQ 14-day (n=257)	PQ Weekly (n=340)	PQ All (n=3228)	No TQ or PQ (n=113)
No G6PD test result	0	254 (9·7)	17 (6·6)	3 (0·9)	274 (8·5)	40 (35·4)
G6PD test result	2685 (100)	2377 (90·3)	240 (93·4)	337 (99·1)	2954 (91·5)	73 (64·6)
G6PD test	2646 (98·5)	2332 (98·1)	232 (96·7)	328 (97·3)	2892 (97·9)	72 (98·6)
G6PD patient card	39 (1·5)	45 (1·9)	8 (3·3)	9 (2·7)	62 (2·1)	1 (1·4)
G6PD status[a]						
Deficient	5 (0·2)	83 (3·5)	14 (5·8)	308 (91·4)	405 (13·7)	4 (5·5)
Intermediate	11 (0·4)	1424 (59·9)	56 (23·3)	13 (3·9)	1493 (50·5)	18 (24·7)
Normal	2669 (99·4)	870 (36·6)	170 (70·8)	16 (4·7)	1056 (35·7)	51 (69·9)

Manaus (n=1840)	TQ (n=921)	PQ 7-day (n=707)	PQ 14-day (n=53)	PQ Weekly (n=113)	PQ All (n=873)	No TQ or PQ (n=46)
No G6PD test result	0	92 (13·0)	5 (9·4)	3 (2·7)	100 (11·5)	15 (32·6)
G6PD test result	921 (100)	615 (87·0)	48 (90·6)	110 (97·3)	773 (88·5)	31 (67·4)
G6PD test	897 (97·4)	598 (97·2)	47 (97·9)	105 (95·5)	750 (97·0)	31 (100)
G6PD patient card	24 (2·6)	17 (2·8)	1 (2·1)	5 (4·5)	23 (3·0)	0
G6PD status[a]						
Deficient	2 (0·2)	37 (6·0)	2 (4·2)	106 (96·4)	145 (18·8)	2 (6·5)
Intermediate	7 (0·8)	400 (65·0)	12 (25·0)	4 (3·6)	416 (53·8)	10 (32·3)
Normal	912 (99·0)	178 (28·9)	34 (70·8)	0	212 (27·4)	19 (61·3)

Porto Velho (n=4186)	TQ (n=1764)	PQ 7-day (n=1924)	PQ 14-day (n=204)	PQ Weekly (n=227)	PQ All (n=2355)	No TQ or PQ (n=67)
No G6PD test result	0	162 (8·4)	12 (5·9)	0	174 (7·4)	25 (37·3)
G6PD test result	1764 (100)	1762 (91·6)	192 (94·1)	227 (100)	2181 (92·6)	42 (62·7)
G6PD test	1749 (99·1)	1734 (98·4)	185 (96·4)	223 (98·2)	2142 (98·2)	41 (97·6)
G6PD patient card	15 (0·9)	28 (1·6)	7 (3·6)	4 (1·8)	39 (1·8)	1 (2·4)
G6PD status[a]						
Deficient	3 (0·2)	46 (2·6)	12 (6·3)	202 (89·0)	260 (11·9)	2 (4·8)
Intermediate	4 (0·2)	1024 (58·1)	44 (22·9)	9 (4·0)	1077 (49·4)	8 (19·0)
Normal	1757 (99·6)	692 (39·3)	136 (70·8)	16 (7·0)	844 (38·7)	32 (76·2)

Values are n (%). TQ, tafenoquine; PQ, primaquine.
[a] G6PD status was determined as a proportion of those patients who had a G6PD test result and defined as deficient (≤4·0 U/g Hb), intermediate (4·1–6·0 U/g Hb), or normal (≥6·1 U/g Hb).

Table S7 Quantitative G6PD testing by age group.

Outcome	≥16 years old (n=5387)	<16 years old (n=639)
No G6PD test result	246 (4·6)	68 (10·6)
G6PD test result	5141 (95·4)	571 (89·4)
G6PD status[a]		
Deficient	363 (7·1)	51 (8·9)
Intermediate	1407 (27·4)	115 (20·1)
Normal	3371 (65·6)	405 (70·9)

Values are n (%).
[a] G6PD status was determined as a proportion of those patients who had a G6PD test result and defined as deficient (≤4·0 U/g Hb), intermediate (4·1–6·0 U/g Hb), or normal (≥6·1 U/g Hb).

Figure S1 Proportion of patients who were G6PD-deficient, G6PD-intermediate, or G6PD-normal, by sex (A). G6PD activity of patients by G6PD status (deficient, intermediate, normal), by sex (B).

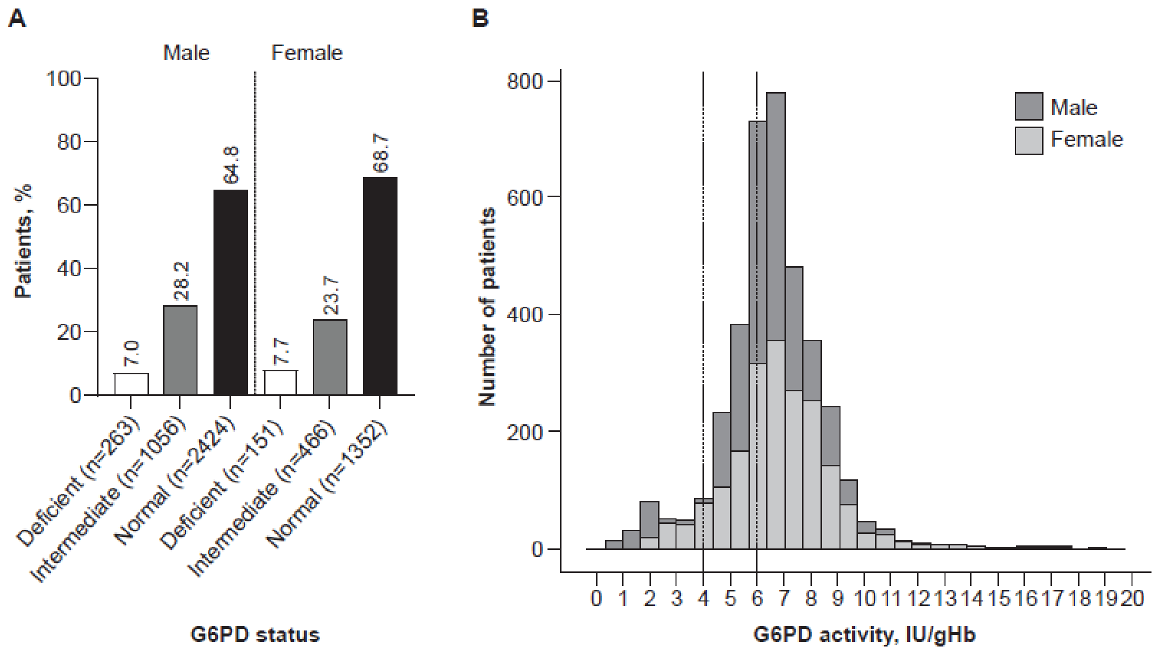

G6PD status was defined as deficient (≤4·0 U/g Hb), intermediate (4·1–6·0 U/g Hb), or normal (≥6·1 U/g Hb).
G6PD, glucose-6-phosphate dehydrogenase.

Figure S2 Proportion of patients who were G6PD-deficient, G6PD-intermediate, or G6PD-normal, by treatment.

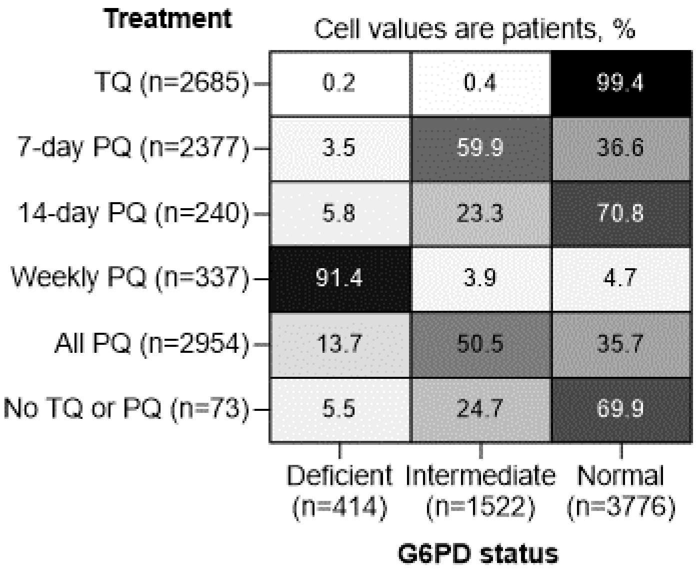

G6PD status was defined as deficient (≤4·0 U/g Hb), intermediate (4·1–6·0 U/g Hb), or normal (≥6·1 U/g Hb). Patients with missing G6PD activity values are not included.
G6PD, glucose-6-phosphate dehydrogenase; TQ, tafenoquine; PQ, primaquine.

Table S8 Primary outcome by municipality: tafenoquine appropriate use and appropriate non-use in patients ≥16 years old according to G6PD activity by facility level and type of facility and overall (primary population).

Outcome in Manaus	Higher/medium-level facilities			Lower-level facilities			Overall (n=1840)
	Hospitals (n=753)	Emergency care (n=667)	All (n=1420)	Basic health units (n=117)	Operational base (n=303)	All (n=420)	
Number of patients ≥16 years	710	584	1294	100	259	359	1653
Total TQ use, n (%)	520 (73·2)	270 (46·2)	790 (61·1)	28 (28·0)	101 (39·0)	129 (35·9)	919 (55·6)
TQ appropriate	519 (73·1)	262 (44·9)	781 (60·4)	28 (28·0)	101 (39·0)	129 (35·9)	910 (55·1)
TQ inappropriate	1 (0·1)	8 (1·4)	9 (0·7)	0	0	0	9 (0·5)
TQ appropriate non-use, n (%)	110 (15·5)	264 (45·2)	374 (28·9)	69 (69·0)	152 (58·7)	221 (61·6)	595 (36·0)
TQ appropriate use and appropriate non-use[a], n/N	629/630	526/534	1155/1164	97/97	253/253	350/350	1505/1514
TQ appropriate use and appropriate non-use[a], % (95% CI)	99·8 (99·1, 100)	98·5 (97·1, 99·4)	99·2 (98·5, 99·6)	100 (96·3, 100)	100 (98·6, 100)	100 (99·0, 100)	99·4 (98·9, 99·7)

Outcome in Porto Velho	Higher/medium-level facilities			Lower-level facilities			Overall (n=4186)
	Hospitals (n=0)	Emergency care (n=3368)	All (n=3368)	Basic health units (n=775)	Operational base (n=43)	All (n=818)	
Number of patients ≥16 years	0	3046	3046	652	36	688	3734
Total TQ use, n (%)	0	1508 (49·5)	1508 (49·5)	242 (37·1)	11 (30·6)	253 (36·8)	1761 (47·2)
TQ appropriate	0	1504 (49·4)	1504 (49·4)	239 (36·7)	11 (30·6)	250 (36·3)	1754 (47·0)
TQ inappropriate	0	4 (0·1)	4 (0·1)	3 (0·5)	0	3 (0·4)	7 (0·2)
TQ appropriate non-use, n (%)	0	1048 (34·4)	1048 (34·4)	335 (51·4)	22 (61·1)	357 (51·9)	1405 (37·6)
TQ appropriate use and appropriate non-use[a], n/N	0/0	2552/2556	2552/2556	574/577	33/33	607/610	3159/3166
TQ appropriate use and appropriate non-use[a], % (95% CI)	–	99·8 (99·6, 100)	99·8 (99·6, 100)	99·5 (98·5, 99·9)	100 (89·4, 100)	99·5 (98·6, 99·9)	99·8 (99·5, 99·9)

[a] Calculated as: (TQ appropriate use + TQ appropriate non-use) / (Total TQ use + TQ appropriate non-use).
TQ, tafenoquine.

Table S9 Daily primaquine (7-day or 14-day) appropriate use and appropriate non-use in patients ≥6 months old according to G6PD activity by facility level and type of facility and overall.

Outcome	Higher/medium-level facilities			Lower-level facilities			Overall (n=6026)
	Hospitals (n=753)	Emergency care (n=4035)	All (n=4788)	Basic health unit (n=892)	Operational base (n=346)	All (n=1238)	
Number of patients aged ≥6 months	751	4033	4784	891	345	1236	6020
Total PQ use, n (%)	183 (24·3)	1995 (49·4)	2178 (45·5)	536 (60·1)	171 (49·4)	707 (57·1)	2885 (47·9)
PQ appropriate use	176 (23·4)	1715 (42·5)	1891 (39·5)	465 (52·2)	163 (47·2)	628 (50·8)	2519 (41·8)
PQ inappropriate use	7 (3·8)	280 (14·0)	287 (13·2)	71 (13·2)	8 (4·7)	79 (11·2)	366 (12·7)
PQ appropriate non-use, n (%)	30 (4·0)	189 (4·7)	219 (4·6)	78 (8·8)	62 (18·0)	140 (11·3)	359 (6·0)
PQ appropriate use and appropriate non-use[a], n/N	206/213	1904/2184	2110/2397	543/614	225/233	768/847	2878/3244
PQ appropriate use and appropriate non-use[a], % (95% CI)	96·7 (93·3, 98·7)	87·2 (85·7, 88·6)	88·0 (86·7, 89·3)	88·4 (85·6, 90·9)	96·6 (93·3, 98·5)	90·7 (88·5, 92·5)	88·7 (87·6, 89·8)

[a] Calculated as: (PQ appropriate use + PQ appropriate non-use) / (Total PQ use + PQ appropriate non-use).
PQ, primaquine.

Table S10 Primaquine appropriate use and appropriate non-use according to G6PD activity in patients ≥6 months old by facility level and type of facility, and overall (primary population).

Outcome in Manaus	Higher/medium-level facilities			Lower-level facilities			Overall (n=1840)
	Hospitals (n=753)	Emergency care (n=667)	All (n=1420)	Basic health units (n=117)	Operational base (n=303)	All (n=420)	
Number of patients ≥6 months	751	666	1417	117	302	419	1836
Total PQ use, n (%)	183 (24·4)	365 (54·8)	548 (38·7)	70 (59·8)	141 (46·7)	211 (50·4)	759 (41·3)
PQ appropriate	176 (23·4)	248 (37·2)	424 (29·9)	66 (56·4)	134 (44·4)	200 (47·7)	624 (34·0)
PQ inappropriate	7 (3·8)	117 (32·1)	124 (22·6)	4 (5·7)	7 (5·0)	11 (5·2)	135 (17·8)
PQ appropriate non-use, n (%)	30 (4·0)	20 (3·0)	50 (3·5)	17 (14·5)	60 (19·9)	77 (18·4)	127 (6·9)
PQ appropriate use and appropriate non-use[a], n/N	206/213	268/385	474/598	83/87	194/201	277/288	751/886
PQ appropriate use and appropriate non-use[a], % (95% CI)	96·7 (93·3, 98·7)	69·6 (64·7, 74·2)	79·3 (75·8, 82·4)	95·4 (88·6, 98·7)	96·5 (93·0, 98·6)	96·2 (93·3, 98·1)	84·8 (82·2, 87·1)

Outcome in Porto Velho	Higher/medium-level facilities			Lower-level facilities			Overall (n=4186)
	Hospitals (n=0)	Emergency care (n=3368)	All (n=3368)	Basic health units (n=775)	Operational base (n=43)	All (n=818)	
Number of patients ≥6 months	0	3367	3367	774	43	817	4184
Total PQ use, n (%)	0	1630 (48·4)	1630 (48·4)	466 (60·2)	30 (69·8)	496 (60·7)	2126 (50·8)
PQ appropriate	0	1467 (43·6)	1467 (43·6)	399 (51·6)	29 (67·4)	428 (52·4)	1895 (45·3)
PQ inappropriate	0	163 (10·0)	163 (10·0)	67 (14·4)	1 (3·3)	68 (13·7)	231 (10·9)
PQ appropriate non-use, n (%)	0	169 (5·0)	169 (5·0)	61 (7·9)	2 (4·7)	63 (7·7)	232 (5·5)
PQ appropriate use and appropriate non-use[a], n/N	–	1636/1799	1636/1799	460/527	31/32	491/559	2127/2358
PQ appropriate use and appropriate non-use[a], % (95% CI)	–	90·9 (89·5, 92·2)	90·9 (89·5, 92·2)	87·3 (84·1, 90·0)	96·9 (83·8, 99·9)	87·8 (84·8, 90·4)	90·2 (88·9, 91·4)

[a] Calculated as: (PQ appropriate use + PQ appropriate non-use) / (Total PQ use + PQ appropriate non-use).
PQ, primaquine.

Table S11 Characteristics of all patients receiving primaquine and by facility level and type of facility and overall (primary population)

Treatment algorithm criteria	Higher/medium-level facilities			Lower-level facilities			Overall (n=3228)
	Hospitals (n=208)	Emergency care (n=2178)	All (n=2386)	Basic health unit (n=613)	Operational base (n=229)	All (n=842)	
Treatment algorithm[a]							
Daily PQ, n	183	1997	2180	537	171	708	2888
Correct, n (%) [95% CI]	172 (94·0) [89·5, 97·0]	1700 (85·1) [83·5, 86·7]	1872 (85·9) [84·3, 87·3]	449 (83·6) [80·2, 86·6]	163 (95·3) [91·0, 98·0]	612 (86·4) [83·7, 88·9]	2484 (86·0) [84·7, 87·3]
Incorrect, n (%)	11 (6·0)	297 (14·9)	308 (14·1)	88 (16·4)	8 (4·7)	96 (13·6)	404 (14·0)
Weekly PQ, n	25	181	206	76	58	134	340
Correct, n (%) [95% CI]	23 (92·0) [74·0, 99·0]	157 (86·7) [80·9, 91·3]	180 (87·4) [82·1, 91·6]	69 (90·8) [81·9, 96·2]	58 (100) [93·8, 100]	127 (94·8) [89·5, 97·9]	307 (90·3) [86·6, 93·2]
Incorrect, n (%)	2 (8·0)	24 (13·3)	26 (12·6)	7 (9·2)	0	7 (5·2)	33 (9·7)
G6PD activity[b]							
Daily PQ, n	183	1997	2180	537	171	708	2888
Correct, n (%) [95% CI]	176 (96·2) [92·3, 98·4]	1716 (85·9) [84·3, 87·4]	1892 (86·8) [85·3, 88·2]	465 (86·6) [83·4, 89·4]	163 (95·3) [91·0, 98·0]	628 (88·7) [86·1, 90·9]	2520 (87·3) [86·0, 88·5]
Incorrect/unknown, n (%)	7 (3·8)	281 (14·1)	288 (13·2)	72 (13·4)	8 (4·7)	80 (11·3)	368 (12·7)
Weekly PQ, n	25	181	206	76	58	134	340
Correct, n (%) [95% CI]	24 (96·0) [79·6, 99·9]	159 (87·8) [82·2, 92·2]	183 (88·8) [83·7, 92·8]	70 (92·1) [83·6, 97·0]	58 (100) [93·8, 100]	128 (95·5) [90·5, 98·3]	311 (91·5) [88·0, 94·2]
Incorrect/unknown, n (%)	1 (4·0)	22 (12·2)	23 (11·2)	6 (7·9)	0	6 (4·5)	29 (8·5)
Age[c]							
Correct, n (%) [95% CI]	208 (100) [98·2, 100]	2176 (99·9) [99·7, 100]	2384 (99·9) [99·7, 100]	612 (99·8) [99·1, 100]	229 (100) [98·4, 100]	841 (99·9) [99·3, 100]	3225 (99·9) [99·7, 100]
Incorrect, n (%)	0	2 (0·1)	2 (0·1)	1 (0·2)	0	1 (0·1)	3 (0·1)
Women of childbearing potential[d], n	38	429	467	126	44	170	637
Non-breastfeeding (1st month) , n (%) [95% CI]	33 (86·8) [71·9, 95·6]	410 (95·6) [93·2, 97·3]	443 (94·9) [92·4, 96·7]	103 (81·7) [73·9, 88·1]	44 (100) [92·0, 100]	147 (86·5) [80·4, 91·2]	590 (92·6) [90·3, 94·5]
Breastfeeding, n (%)	0	0	0	0	0	0	0
Unknown breastfeeding, n (%)	5 (13·2)	19 (4·4)	24 (5·1)	23 (18·3)	0	23 (13·5)	47 (7·4)
Non-pregnant, n (%) [95% CI]	38 (100) [90·7, 100]	429 (100) [99·1, 100]	467 (100) [99·2, 100]	126 (100) [97·1, 100]	44 (100) [92·0, 100]	170 (100) [97·9, 100]	637 (100) [99·4, 100]
Pregnant	0	0	0	0	0	0	0

[a] Based on normal/intermediate G6PD activity (≥4·1 U/g Hb) for daily PQ and deficient G6PD activity (<4·1 U/g Hb) for weekly PQ, age ≥6 months, non-breastfeeding (1st month), non-pregnant.
[b] Based on normal/intermediate G6PD activity (≥4·1 U/g Hb) for daily PQ and deficient G6PD activity (<4·1 U/g Hb) for weekly PQ.
[c] Based on age ≥6 months receiving daily or weekly PQ.
[d] Based on female patients aged ≥16 years to <50 years.
PQ, primaquine.

Table S12 Baseline socio-demographic and clinical characteristics of patients with mixed *P. vivax*/*P. falciparum* infections, by treatment group.

Characteristic	TQ (n=5)	PQ				No TQ or PQ	Overall
		7-day (n=34)	14-day (n=4)	Weekly (n=2)	All PQ (n=40)	(n=4)	(n=49)
Sex							
Male	4 (80·0)	23 (67·6)	4 (100)	2 (100)	29 (72·5)	3 (75·0)	36 (73·5)
Female	1 (20·0)	11 (32·4)	0	0	11 (27·5)	1 (25·0)	13 (26·5)
Childbearing potential[a]	0	8 (72·7)	0	0	8 (72·7)	1 (100)	9 (69·2)
Breastfeeding[b]							
Yes	0	1 (12·5)	0	0	1 (12·5)	0	1 (11·1)
No	0	7 (87·5)	0	0	7 (87·5)	1 (100)	8 (88·9)
Pregnant[b]							
Yes	0	0	0	0	0	1 (100)	1 (11·1)
No	0	8 (100)	0	0	8 (100)	0	8 (88·9)
Age, years	41·2 (13·1)	32·9 (17·1)	33·8 (12·7)	47·5 (6·4)	33·7 (16·5)	39·5 (20·6)	34·9 (16·4)
Ethnicity							
Mixed	5 (100)	33 (97·1)	4 (100)	1 (50·0)	38 (95·0)	4 (100)	47 (95·9)
White	0	1 (2·9)	0	1 (50·0)	2 (5·0)	0	2 (4·1)
Black	0	0	0	0	0	0	0
Asian	0	0	0	0	0	0	0
Indigenous	0	0	0	0	0	0	0
Age class, years							
Birth to <5	0	1 (2·9)	0	0	1 (2·5)	0	1 (2·0)
≥5 to <16	0	3 (8·8)	0	0	3 (7·5)	0	3 (6·1)
≥16 to <30	1 (20·0)	12 (35·3)	1 (25·0)	0	13 (32·5)	2 (50·0)	16 (32·7)
≥30 to <50	2 (40·0)	11 (32·4)	3 (75·0)	1 (50·0)	15 (37·5)	0	17 (34·7)
≥50 to <70	2 (40·0)	7 (20·6)	0	1 (50·0)	8 (20·0)	2 (50·0)	12 (24·5)
≥70	0	0	0	0	0	0	0
Weight class, kg							
≥5 to <50	0	5 (14·7)	0	0	5 (12·5)	0	5 (10·2)
≥50 to <70	1 (20·0)	11 (32·4)	1 (25·0)	0	12 (30·0)	1 (25·0)	14 (28·6)
≥70 to <90	2 (40·0)	10 (29·4)	2 (50·0)	2 (100·0)	14 (35·0)	3 (75·0)	19 (38·8)
≥90 to <120	0	2 (5·9)	1 (25·0)	0	3 (7·5)	0	3 (6·1)
≥120	0	0	0	0	0	0	0
Missing	2 (40·0)	6 (17·6)	0	0	6 (15·0)	0	8 (16·3)
Haemoglobin level, g/dL							
n	5	31	3	1	35	2	42
Mean (SD)	14·1 (2·8)	13·4 (3·4)	10·5 (5·1)	8·9	13·0 (3·6)	12·2 (2·2)	13·1 (3.5)
<7·0 g/dL	0	1 (3·2)	0	0	1 (2·9)	0	1 (2·4)
Education							
Illiterate	0	2 (5·9)	0	0	2 (5·0)	0	2 (4·1)
1st to 5th year	1 (20·0)	3 (8·8)	1 (25·0)	1 (50·0)	5 (12·5)	0	6 (12·2)
5th year	0	5 (14·7)	0	0	5 (12·5)	1 (25·0)	6 (12·2)
6th-9th year	4 (80·0)	2 (5·9)	2 (50·0)	0	4 (10·0)	0	8 (16·3)
Complete basic education	0	5 (14·7)	0	0	5 (12·5)	1 (25·0)	6 (12·2)
Incomplete high school	0	8 (23·5)	0	0	8 (20·0)	1 (25·0)	9 (18·4)
Complete high school	0	5 (14·7)	1 (25·0)	1 (50·0)	7 (17·5)	0	7 (14·3)
Incomplete college	0	1 (2·9)	0	0	1 (2·5)	1 (25·0)	2 (4·1)
Complete college	0	1 (2·9)	0	0	1 (2·5)	0	1 (2·0)
Not applicable	0	2 (5·9)	0	0	2 (5·0)	0	2 (4·1)
Missing	0	0	0	0	0	0	0
Occupation							
Agriculture	0	2 (5·9)	0	0	2 (5·0)	0	2 (4·1)
Livestock	0	0	0	0	0	0	0
Household	1 (20·0)	7 (20·6)	2 (50·0)	0	9 (22·5)	0	10 (20·4)
Tourism	1 (20·0)	0	0	0	0	0	1 (2·0)
Gold digging	0	1 (2·9)	1 (25·0)	0	2 (5·0)	0	2 (4·1)
Farming	0	0	0	0	0	0	0
Hunting/fishing	0	0	0	0	0	0	0
Construction	0	0	0	0	0	0	0
Mining	0	0	0	0	0	0	0
Traveller	0	1 (2·9)	0	1 (50·0)	2 (5·0)	0	2 (4·1)
Other	3 (60·0)	22 (64·7)	1 (25·0)	1 (50·0)	24 (60·0)	4 (100)	31 (63·3)
Missing	0	1 (2·9)	0	0	1 (2·6)	0	1 (2·0)

Data are n (%) and mean (standard deviation).
[a] Denominator for % is the number of females.
[b] Denominator for % is the number of females of childbearing potential (defined as age ≥16 to <50 years).

Table S13 G6PD testing and G6PD status of patients with mixed infections, by treatment group.

Characteristic	TQ (n=5)	PQ				No TQ or PQ (n=4)	Overall (n=49)
		7-day (n=34)	14-day (n=4)	Weekly (n=2)	All (n=40)		
G6PD test							
No G6PD test result	0	3 (8·8)	1 (25·0)	1 (50·0)	5 (12·5)	2 (50·0)	7 (14·3)
G6PD test result	5 (100)	31 (91·2)	3 (75·0)	1 (50·0)	35 (87·5)	2 (50·0)	42 (85·7)
G6PD quantitative test	5 (100)	30 (96·8)	3 (100)	1 (100)	34 (97·1)	2 (100)	41 (97·6)
G6PD patient card	0	1 (3·2)	0	0	1 (2·9)	0	1 (2·4)
G6PD status							
Deficient	0	1 (3·2)	0	1 (100)	2 (5·7)	0	2 (4·8)
Intermediate	0	12 (38·7)	0	0	12 (34·3)	1 (50·0)	13 (31·0)
Normal	5 (100)	18 (58·1)	3 (100)	0	21 (60·0)	1 (50·0)	27 (64·3)

Data are n (%). TQ, tafenoquine, PQ, primaquine.
G6PD status was defined as deficient (≤4·0 U/g Hb), intermediate (4·1–6·0 U/g Hb), or normal (≥6·1 U/g Hb). G6PD normal represented ≥70% and G6PD deficient <30% of the local population median G6PD activity.

Table S14 Malaria history of patients with mixed infections, by facility level.

Characteristic	Higher/medium-level facilities			Lower-level facilities			Overall (n=49)
	Hospitals (n=8)	Emergency care (n=24)	All (n=32)	Basic health units (n=17)	Operational base (n=0)	All (n=17)	
Diagnostic test							
Thick blood smear	7 (87·5)	24 (100)	31 (96·9)	15 (88·2)	0	15 (88·2)	46 (93·9)
Rapid diagnostic test	1 (12·5)	0	1 (3·1)	1 (5·9)	0	1 (5·9)	2 (4·1)
Polymerase chain reaction	0	0	0	1 (5·9)	0	1 (5·9)	1 (2·0)
Parasitaemia, per mm^3							
n	0	4	4	0	0	0	4
Mean (SD)	–	11·0 (12·8)	11·0 (12·8)	–	–	–	11·0 (12·8)
Semi-quantitative parasitaemia[a]							
< half cross	1 (12·5)	3 (12·5)	4 (12·5)	5 (29·4)	0	5 (29·4)	9 (18·4)
Half cross	1 (12·5)	0	1 (3·1)	1 (5·9)	0	1 (5·9)	2 (4·1)
1 cross	1 (12·5)	1 (4·2)	2 (6·3)	1 (5·9)	0	1 (5·9)	3 (6·1)
2 crosses	1 (12·5)	9 (37·5)	10 (31·3)	7 (41·2)	0	7 (41·2)	17 (34·7)
3 crosses	3 (37·5)	10 (41·7)	13 (40·6)	1 (5·9)	0	1 (5·9)	14 (28·6)
4 crosses	0	1 (4·2)	1 (3·1)	0	0	0	1 (2·0)
Missing	1 (12·5)	0	1 (3·1)	2 (11·8)	0	2 (11·8)	3 (6·1)
Blood transfusion in last 60 days							
Yes	0	0	0	0	0	0	0
No	8 (100)	22 (91·7)	30 (93·8)	16 (94·1)	0	16 (94·1)	46 (93·9)
Missing	0	2 (8·3)	2 (6·3)	1 (5·9)	0	1 (5·9)	3 (6·1)
P. falciparum in the last 40 days							
Yes	3 (37·5)	4 (16·7)	7 (21·9)	1 (5·9)	0	1 (5·9)	8 (16·3)
No	5 (62·5)	20 (83·3)	25 (78·1)	16 (94·1)	0	16 (94·1)	41 (83·7)

Data are n (%) or mean (standard deviation). TQ, tafenoquine, PQ, primaquine.
[a] Half-cross (200–300 parasites/mm^3); 1 cross (301–500 parasites/mm^3); 2 crosses (501–10,000 parasites/mm^3); 3crosses (10,001–100,000 parasites/mm^3); and 4 crosses (>100,001 parasites/mm^3).

Table S15 Pre-treatment haemoglobin levels by treatment group (primary population).

Characteristic	TQ (n=2685)	PQ				No TQ or PQ (n=113)
		7-day (n=2631)	14-day (n=257)	Weekly (n=340)	All (n=3228)	
Missing test	0	254 (9·7)	17 (6·6)	3 (0·9)	274 (8·5)	40 (35·4)
Number with test	2685 (100)	2377 (90·3)	240 (93·4)	337 (99·1)	2954 (91·5)	73 (64·6)
Mean (SD), g/dL	14·0 (2·5)	14·5 (2·8)	14·1 (2·9)	14·9 (3·6)	14·5 (2·9)	12·8 (3·1)
Median (Q1, Q3), g/dL	14·1 (12·5, 15·5)	14·4 (12·7, 16·2)	14·2 (12·5, 15·4)	14·6 (12·5, 16·8)	14·4 (12·7, 16·2)	12·4 (10·8, 14·8)
Min, max, g/dL	4·0, 24·8	4·0, 24·7	5·0, 25·0	4·0, 24·9	4·0, 25·0	6·6, 20·9
<7·0 g/dL, n (%)	18 (0·7)	14 (0·6)	3 (1·3)	4 (1·2)	21 (0·7)	3 (4·1)

Table S16 Haemoglobin levels, overall and by facility level and municipality (primary population).

Overall	High/medium-level facilities			Lower-level facilities			Overall (n=6026)
	Hospital (n=753)	Emergency care (n=4035)	All (n=4788)	Basic health unit (n=892)	Operational base (n=346)	All (n=1238)	
Number with test	741	3811	4552	825	335	1160	5712
Mean (SD), g/dL	13·6 (2·5)	14·3 (2·7)	14·2 (2·7)	14·4 (2·9)	14·1 (3·3)	14·3 (3·0)	14·2 (2·7)
Median (Q1, Q3) , g/dL	13·8 (12·3, 15·3)	14·3 (12·6, 16·0)	14·2 (12·6, 15·8)	14·4 (12·7, 16·0)	13·8 (11·9, 15·5)	14·2 (12·5, 15·8)	14·2 (12·6, 15·8)
Min, max, g/dL	4·9, 21·3	4·0, 24·8	4·0, 24·8	4·0, 25·0	4·0, 24·9	4·0, 25·0	4·0, 25·0
<7·0 g/dL, n (%)	9 (1·2)	19 (0·5)	28 (0·6)	10 (1·2)	4 (1·2)	14 (1·2)	42 (0·7)

Manaus	High/medium-level facilities			Lower-level facilities			Overall (n=1840)
	Hospital (n=753)	Emergency care (n=667)	All (n=1420)	Basic health unit (n=117)	Operational base (n=303)	All (n=420)	
Number with test	741	581	1322	110	293	403	1725
Mean (SD), g/dL	13·6 (2·5)	14·4 (3·0)	14·0 (2·7)	14·2 (3·1)	14·1 (3·3)	14·1 (3·3)	14·0 (2·9)
Median (Q1, Q3) , g/dL	13·8 (12·3, 15·3)	14·2 (12·5, 16·1)	14·0 (12·4, 15·5)	14·3 (12·2, 15·8)	13·7 (12·0, 15·5)	13·9 (12·1, 15·6)	14·0 (12·3, 15·5)
Min, max, g/dL	4·9, 21·3	5·2, 24·8	4·9, 24·8	4·2, 23·5	4·0, 24·9	4·0, 24·9	4·0, 24·9
<7·0 g/dL, n (%)	9 (1·2)	2 (0·3)	11 (0·8)	2 (1·8)	4 (1·4)	6 (1·5)	17 (1·0)

Porto Velho	High/medium-level facilities			Lower-level facilities			Overall (n=4186)
	Hospital (n=0)	Emergency care (n=3368)	All (n=3368)	Basic health unit (n=775)	Operational base (n=43)	All (n=818)	
Number with test	0	3230	3230	715	42	757	3987
Mean (SD), g/dL	–	14·3 (2·6)	14·3 (2·6)	14·4 (2·9)	14·0 (2·9)	14·4 (2·9)	14·3 (2·7)
Median (Q1, Q3) , g/dL	–	14·3 (12·7, 15·9)	14·3 (12·7, 15·9)	14·4 (12·7, 16·0)	14·1 (11·6, 15·9)	14·4 (12·7, 16·0)	14·3 (12·7, 16·0)
Min, max, g/dL	–	4·0, 24·6	4·0, 24·6	4·0, 25·0	8·1, 20·4	4·0, 25·0	4·0, 25·0
<7·0 g/dL, n (%)	0	17 (0·5)	17 (0·5)	8 (1·1)	0	8 (1·1)	25 (0·6)

20

Table S17 Day 5 follow-up outcomes (safety population).

Outcome	TQ	PQ				Overall
		7-day	14-day	Weekly	All	
Overall, n	2685	2631	257	340	3228	5913
Follow-up completed	622 (23·2)	172 (6·5)	19 (7·4)	24 (7·1)	215 (6·7)	837 (14·2)
Signs of haemolysis[a]						
Missing	64 (10·3)	21 (12·2)	2 (10·5)	3 (12·5)	26 (12·1)	90 (10·8)
Yes	56 (9·0)	8 (4·7)	1 (5·3)	0	9 (4·2)	65 (7·8)
No	502 (80·7)	143 (83·1)	16 (84·2)	21 (87·5)	180 (83·7)	682 (81·5)
Manaus, n	921	707	53	113	873	1794
Follow-up completed	253 (27·5)	72 (10·2)	10 (18·9)	13 (11·5)	95 (10·9)	348 (19·4)
Signs of haemolysis[a]						
Missing	57 (22·5)	15 (20·8)	1 (10·0)	3 (23·1)	19 (20·0)	76 (21·8)
Yes	6 (2·4)	0	1 (10·0)	0	1 (1·1)	7 (2·0)
No	190 (75·1)	57 (79·2)	8 (80·0)	10 (76·9)	75 (78·9)	265 (76·1)
Porto Velho, n	1764	1,924	204	227	2355	4119
Follow-up completed	369 (20·9)	100 (5·2)	9 (4·4)	11 (4·8)	120 (5·1)	489 (11·9)
Signs of haemolysis[a]						
Missing	7 (1·9)	6 (6·0)	1 (11·1)	0	7 (5·8)	14 (2·9)
Yes	50 (13·6)	8 (8·0)	0	0	8 (6·7)	58 (11·9)
No	312 (84·6)	86 (86·0)	8 (88·9)	11 (100·0)	105 (87·5)	417 (85·3)

[a] Calculated for those patients who completed day 5 follow-up.

21

Study Protocol

**Protocol No**	MMV_TQ_18_02
**Products**	Tafenoquine<br>Primaquine
**Study Title**	**TRUST Study**<br>**Operational Feasibility of Appropriate *Plasmodium Vivax* Radical Cure with Tafenoquine or Primaquine after Quantitative G6PD Testing in Brazil**
**Study Sponsor**	Health Surveillance Secretariat/ Coordination of Zoonoses and Vector Transmission Diseases Surveillance/Ministry of Health, Brazil and Medicines for Malaria Venture
**Date of Protocol**	21June2019
**Amendment**	06Feb2020
**Amendment 2**	17Jul2020
**Amendment 3**	12May2021
**Amendment 4**	23Sep2021

**Responsible Parties**	
Principal Investigator	Marcus Lacerda<br>[redacted]

Study sponsor contact person	Stephan Duparc, MD<br>[redacted]<br><br>Secretaria de Vigilância em Saúde Coordenação-Geral de Vigilância de Zoonoses eDoenças de Transmissão Vetorial<br>[redacted]

Confidentiality: This protocol is the property of Medicines for Malaria Venture and may not – in full or in part – be transferred, reproduced, published, or otherwise used without the express permission of Medicines forMalaria Venture.

Document History

Document	Date	Summary of Change
Protocol V1.0	21/06/2019	Not applicable
Protocol V2.0	27/01/2020	Modification of wording to clarify the Observational and non-interventional characteristics of the study as CONEP requested.
Protocol V3.0 (Amendment 1)	06/02/2020	Addition of a pilot phase in view of delayed availability of tafenoquine
	06/02/2020	Update of information on approval of tafenoquine by ANVISA
Protocol V4.0 (Amendment 2)	17/07/2020	Removal of pilot phase due to delayed start with Covid-19
		Modification of primary endpoint calculation to reflect updated Malaria Treatment Guidelines of Ministry of Health<br>Revised timelines for the study due to the COVID-19 pandemic.
Protocol V5.0 (Amendment 3)	25/05/2021	Revised timelines for the study.
Protocol V6.0 (Amendment 4)	23/09/2021	Revised inclusion criteria

Approval

TRuST study

Operational Feasibility of Appropriate *Plasmodium vivax* Radical Cure with Tafenoquine or Primaquine After Quantitative G6PD Testing in Brazil

Protocol N° MMV_TQ_18_02 Amendment 4

Version 6

Dated 23 Sep 2021

The signatures of the representatives of Doctor Heitor Vieira Dourado Tropical Medicine Foundation (the principal investigator) and Medicines for Malaria Venture (MMV) below constitute their approval of this protocol and provide the necessary assurances that this study will be conducted in compliance with Good Pharmacoepidemiology Practice (GPP) guidelines, the ethical principles arising from the Declaration of Helsinki revised in 2013 and all current local regulations.

For Doctor Heitor Vieira Dourado Tropical Medicine Foundation

Dr Marcus Vinivius Guimarães de Lacerda
MD, Principal Investigator

Signature [redacted] Date 24/09/2021

For the Medicines for Malaria Venture (MMV)

Dr Stephan Duparc, MD
Chief Medical Officer

Signature: [redacted] Date: 24/09/2021

Table of contents

Document History 3
Approval 4
Table of contents 5
List of Tables 7
List of Figures 8
1 LIST OF ABBREVIATIONS 9
2 SYNOPSIS 10
3 AMENDMENTS AND UPDATES 14
5 BACKGROUND AND RATIONALE 16
5.1 Background 16
5.2 Study Rationale 18
6 OBJECTIVES AND ENDPOINTS 19
6.1 Objectives 19
6.1.1 Primary Objective 19
6.1.2 Secondary Objectives 19
6.2 Endpoints 19
6.2.1 Primary endpoint 19
6.2.2 Secondary endpoints 19
7 HYPOTHESIS 20
8 STUDY DESIGN 21
9 STUDY POPULATION 25
9.1 Health Care Facilities 25
9.2 Patients 25
9.2.1 Inclusion Criteria 25
9.2.2 Exclusion Criteria 25
10 MEDICINAL PRODUCTS AND G6PD DIAGNOSTIC TEST 26
11 STUDY ASSESSMENTS 27
11.1 Patient Data 27
11.2 Cost Data 28

12 SAFETY REPORTING 29

13 STATISTICAL METHODS 30

13.1 Study Size 30

13.2 Statistical Analyses 31

13.2.1 General Considerations........ 31

13.2.2 Analyzed Populations 31

13.2.3 Data Analysis 31

13.2.4 Interim Analyses 32

13.2.5 Final Analyses 32

13.3 Bias of the Study 33

13.3.1 Selection Bias 33

13.3.2 Information Bias........ 33

13.3.3 Confounding Bias........ 33

14 DATA HANDLING........ 34

15 ETHICAL CONDUCT OF THE STUDY 35

15.1 Ethical Considerations........ 35

15.2 Institutional Review Board/Independent Ethics Committee........ 35

15.3 Independent Study Oversight Committee........ 35

15.4 Benefits 35

15.5 Risks 36

15.6 Informed Consent 36

15.7 Data Protection 38

15.8 Adherence to the Protocol........ 38

15.9 Protocol Amendment........ 38

15.10 Retention of Patient Records........ 38

15.11 Confidentiality 38

16 PLANS FOR DISSEMINATING AND COMMUNICATING STUDYREPORT 39

17 REFERENCES 40

List of Tables

Table 1 Study Milestones and Timelines 15

Table 2 Precision and 95%CI obtained for percentages from 80% to 99% with study sizes of 400, 500, 1000, 2000 and 5000 30

List of Figures

Figure 1: Study Flow 24

1 LIST OF ABBREVIATIONS

Abbreviation	Definition
AHA	Acute hemolytic anemia
ANVISA	Brazilian health regulatory agency
CI	Confidence interval
CQ	Chloroquine
CRF	Case report form
CRO	Contract research organization
EC	Ethics committee
e-CRF	Electronic case report form
FDA	Food and Drug Administration
FMT-HVD	Tropical Medicine Foundation Doctor Heitor Vieira
G6PD	Glucose-6-phosphate dehydrogenase
GPP	Good pharmacoepidemiology practice
HCP	Health care provider
ICF	Informed consent form
ICH	International conference on harmonization
IEC	Independent ethics committee
IRB	Institutional review board
ISOC	Independent Study oversight committee
ISPE	International society for pharmacoepidemiology
MAH	Marketing authorization holder
NMCP	National malaria control programme
P. vivax	Plasmodium vivax
PI	Principal investigator
PQ	Primaquine
SAP	Statistical analysis plan
SIVEP-Malaria	Epidemiological surveillance system for malaria
TLS	Transport layer security
TQ	Tafenoquine
WHO	World Health Organization

2 SYNOPSIS

**Title**	**Operational Feasibility of Appropriate *Plasmodium Vivax* Radical Cure with Tafenoquine or Primaquine after Quantitative G6PD Testing in Brazil**
**Protocol number**	MMV_TQ_18_02
**Objectives**	<u>Primary objective</u><br>To investigate whether *Plasmodium vivax* (*P. vivax*) patients ≥16 years are treated with tafenoquine (TQ) in accordance with the appropriate level of glucose-6-phosphate dehydrogenase (G6PD) enzyme activity.<br><u>Secondary objectives</u><br>- To investigate whether *P. vivax* patients ≥6 months are treated with primaquine (PQ) in accordance with the appropriate level of G6PD enzyme activity.<br>- To describe the characteristics of patients treated with TQ or PQ.<br>- To measure frequency of drug-induced acute hemolytic anemia (AHA) and hospitalization due to drug-induced AHA.
**Study Endpoints**	<u>Primary endpoint</u><br>- Percentage of *P. vivax* patients^ aged ≥16 years treated or not with TQ in accordance with the appropriate level of G6PD enzyme activity:<br>Among patients aged ≥16 years^:<br>$\frac{\text{Appropriate use of TQ}^{*} + \text{Appropriate non-use of TQ}^{**}}{\text{Total use of TQ}^{***} + \text{Appropriate non-use of TQ}^{**}}$<br>^ excluding those who were treated for *P. vivax* malaria in the last 60 days<br>*Number of patients with ≥70% G6PD activity treated with TQ<br>** Number of patients with <70% G6PD activity not treated with TQ<br>*** Number of patients treated with TQ<br><br><u>Secondary endpoints</u><br>- Percentage of *P. vivax* patients aged ≥6 months treated or not with daily PQ in accordance with the appropriate level of G6PD enzyme activity:<br>Among patients aged ≥6 months:<br>$\frac{\text{Appropriate use of daily PQ}^{*} + \text{Appropriate non-use of daily PQ}^{**}}{\text{Total use of PQ}^{***} + \text{Appropriate non-use of daily PQ}^{**}}$<br>*Number of patients with ≥30% G6PD activity treated with daily PQ<br>** Number of patients with <30% G6PD activity not treated with daily PQ<br>*** Number of patients treated with daily PQ<br>- Description of the characteristics of patients treated with TQ or with PQ<br>- Frequency of confirmed drug-induced AHA and hospitalization due to drug-induced AHA

**Study Design**	This is a non-interventional, observational study conducted in Brazil in patients with *P. vivax* malaria. The study will be conducted in the municipalities of Manaus (Amazonas State) and Porto Velho (Rondônia State).<br>G6PD tests and TQ will be supplied to health care facilities by the Municipal Health Authorities using the usual supply route for drugs and diagnostics. PQ and other anti-malarial drugs are already available in Brazil.<br>Designated personnel at health care facilities will be trained on the quantitative G6PD test procedure and the radical cure treatment algorithm by the Principal Investigator (PI) staff and the municipality authorities using educational materials developed by the sponsor in collaboration with the Brazilian Ministry of Health (National Malaria Control Programme [NMCP]) (intended for roll-out during scale-up of TQ introduction).<br>The design is based on secondary use of data collected routinely for all patients with malaria in the Epidemiological Surveillance System for Malaria (SIVEP-Malaria) by the Brazilian NMCP. Data from all patients with malaria are routinely collected in the SIVEP form by health care providers (HCPs) and entered in the SIVEP database by the municipality staff.<br>In accordance with the NMCP, the SIVEP form has been adapted to collect information about G6PD testing, TQ treatment and signs of hemolysis. Within the study period, and on a regular basis, retrospective data from all patients will be entered into a new database, the study database, by the municipality staff. Relevant data will be automatically exported, on a weekly basis, to the SIVEP database. The project staff will only have access to de-identified data in line with the access rights that will be attributed to them in the system. Only municipal staff will have access to identified patient data.<br>In addition to the data collected from the SIVEP forms, PI staff will request the 2 referral hospitals likely to see all hospitalizations for AHA to regularly screen electronic admission records for patients presenting signs of AHA (renal failure, jaundice, blood transfusion, malaria). All identified cases will be investigated using hospital records and SIVEP forms. Information about confirmed drug-induced AHA will be linked with the patient record in the study database. PI staff will also contribute to pharmacovigilance training. Physicians at higher-level health facilities will report side effects through the official Brazilian health regulatory agency (ANVISA) VigiMed system.<br>Finally, the additional costs of implementing G6PD testing and TQ will be collected alongside the study in the health care facilities.<br>As the study is based on retrospective data collection, G6PD testing and treatment of patients with TQ or PQ will be conducted in line with the treatment policy i.e., independent from the study.<br>The study will be conducted in a phased manner:<br>- 1st phase (approximately 3 months): Training and provision of G6PD tests and TQ will be initially limited to the 10 high and medium-level health facilities (referralhospitals, hospitals, emergency care units, polyclinics) involved in the pilot phase. Data will be collected for *P. vivax* malaria patients managed at thesehealth care facilities.<br>An interim analysis will be conducted after data from 600 *P. vivax* patients ≥16 years who have not been treated for *P. vivax* malaria in the last 60 days are collected in the study database, in order to decide whether the study could be extended to lower-level health facilities. The decision will be made by a Independent Study Oversight Committee (ISOC).

	If the interim results of the 1st phase are deemed unsatisfactory, the ISOC may decide to not extend the study to lower-level health facilities until improvements are made in the educational programme and/or additional HCP support is implemented. Additional interim analyses may be conducted as appropriate.<br>- 2nd phase (approximately 9 months): if approved by the ISOC, the study willbe extended to lower-level health facilities (basic health units, basic family health units and primary care) and to additional high and medium-level health facilities.After training of the staff, G6PD tests and TQ will be supplied to these healthcare facilities by the municipal health authorities.<br>During this 2nd phase, data will continue to be collected for *P. vivax* patients managed by the higher-level health facilities of the 1st phase.<br>• An additional interim analysis will be conducted after data from 600 *P. vivax* patients ≥16 years who have not been treated for *P. vivax* malaria in the last 60 days from lower-level facilities are collected in the study database (approximately 3 months after the beginning of the 2nd phase).<br>The study will continue while interim analyses are being conducted.<br>Final results will be reviewed and validated by the ISOC.<br>It is anticipated that it will take approximately 15-months to carry out the study.
**Health care facilities**	The study will be conducted in the municipalities of Manaus and Porto Velho.<br>1st phase: data will be collected for *P. vivax* patients managed at approximately 10 high and medium-level health facilities (referral hospitals, hospitals, emergency care units, polyclinics).<br>2nd phase (if approved by the ISOC): data will be collected for *P. vivax* patients managed at approximately 100 lower-level health facilities (basic health units, basic family health units and primary care that are less than 24h away from emergency care) and approximately 10 additional high and medium-level health facilities.<br>During the 2nd phase, data will continue to be collected for patients managed in high and medium-level health facilities of the 1st phase.
**Number of Patients**	It is anticipated that data from approximately 16,000 patients will be included in the study database over the 1-year study duration (approximately 10,000 patients from high and medium-level health facilities and approximately 6,000 patients from lower-level health facilities). Due to the retrospective study design and de-identified nature of data reviewed by the study team, and thus the very low risk attributed to the study for patients, patient informed consent for secondary use of their data will be recorded by the HCP in the SIVEP form.
**Patient Selection Criteria**	**Inclusion criteria**<br>A patient who has consented/assented must meet the following criteria to be eligible for the study:<br>- Diagnosed with mono-species *P. vivax* malaria (parasitologically confirmed through microscopy or rapid diagnostic test) and older than 6 months of age.<br>- Diagnosed with *P. vivax / P. falciparum* malaria mixed infection (parasitologically confirmed through microscopy or rapid diagnostic test) and older than 6 months of age.<br>- Diagnosed negative for *P. vivax* mono-species malaria or *P. vivax / P. falciparum* malaria mixed infection but had a positive diagnosis in the past.<br>**Exclusion criteria**<br>None.

**Statistical Methods**	A detailed Statistical Analysis Plan will be developed and validated before database lock.<br>Statistical analyses will be carried out using SAS software version 9.4 or higher.<br>Analysis will be purely descriptive (no tests performed).<br>Continuous variables will be described by their mean, standard deviation, median, quartiles 1 and 3, extreme values (minimum and maximum) and the number of missing data.<br>Categorical variables will be described by the total and percentage of each response and the number of missing data. Missing data will not be included in the calculation of percentages.<br>95% confidence intervals will be provided when relevant.<br>Only de-identified data will be used for statistical analyses.
**Planned study period (estimates)**	Start of 1st Phase data collection (high and medium-level facilities only): July 2021;First interim analysis: September 2021<br>Start of data collection (lower-level facilities and additional high and medium-levelfacilities): October 2021<br>Second interim analysis: January 2022<br>End of data collection: July 2022<br>Final analysis: September 2022<br>Final study report: January 2023

3 AMENDMENTS AND UPDATES

Amendment 1:

Although the TQ has been approved by the National Health Regulatory Agency (ANVISA) on October 29, 2019, the company holding the marketing authorization needs to complete post-regulatory approval steps such as drug pricing and adapted packaging. It is estimated that TQ is only available in the cities of Manaus and Porto Velho by May 2020.

In view of this delay, the Ministry of Health and public managers of the municipalities plan to start the pilot with a phase that will involve the use of the G6PD test before prescribing radical cure with primaquine (PQ), as recommended by the World Health Organization (WHO). Moreover, in the revised malaria treatment guidelines of the Brazilian Ministry of Health issued in December 2019, G6PD testing before primaquine radical cure is mandatory at health facilities that have the equipmentto do so.

The pilot phase will allow the health services to refine the training approach, training materials, patient counselling etc. and learning from the experience with the introduction of G6PD testing for subsequent phases. The pilot phase will be limited to the 10 higher level health units selected for the first phase of the TRUST implementation. All health professionals at these units will be trained by professionals from the State Central Laboratory (LACEN) and State Health Department in both cities, Manaus and Porto Velho. After training, these 10 health units will conduct G6PD tests before prescribing the use of primaquine.

Update:

The Ministry of Health has updated the SIVEP system to include the additional variables (including G6PD activity, Hb level, TQ as a treatment option, Day 5 follow-up, weight) in the TRUST study. In view of this, the data entry will be done exclusively by the municipal staff into the SIVEP database. There is no need for an additional data entry system as originally envisaged.

Tafenoquine has been registered by ANVISA in October 2019.

The number of patients was increased as a new phase of data collection was included in the project. It is estimated that approximately 2,000 patients, treated in the 10 high and medium complexity units, will be included in this phase.

Amendment 2:

Removal of pilot phase: With the delayed start of TRuST due to Covid-19, the issue regarding the availability of TQ has been resolved. In view of this, the study will start with the 1st phase in line with the original protocol and there is no longer a need for a pilot phase.

Revised definition for primary endpoint: The revised malaria treatment guidelines issued by the Ministry of Health in 2020, specify that patients who have been treated for *P. vivax* malaria in the last 60 days should be treated with a different treatment regimen (Blood stage treatment: Artemether-Lumefantrine; Liver stage treatment: 14-days course of primaquine 0.5mg/kg). NB: In the past, all *P. vivax* patients were treated with 3-day chloroquine + 7 days primaquine (0.5 mg/kg). In view of this the primary endpoint will exclude all *P. vivax* malaria patients who have been treated for *P. vivax* malaria in the last 60 days.

Revised timeline: Study start date: October 2020.

Amendment 3: Timeline revised. Study start date: July 2021.

Amendment 4: Inclusion criteria revised.

4 STUDYMILESTONES AND TIMELINES

The planned study milestones and timelines are described in the table below.

Table 1 Study Milestones and Timelines

Milestone	Estimated Date
Ethics submission	July 2019
Ethics Approval	November 2019
Submission of notification	January 2020
Submission of amendment 2	July 2020
Ethics approval	August 2020
Submission of amended protocol amendment 3	June 2021
Ethics approval	June 2021
Start of data collection (higher and medium-level facilities only)	July 2021
First interim analysis	September 2021
Start of data collection (lower-level facilities and additional higher and medium-level facilities)	October 2021
Second interim analysis	January 2022
End of data collection	July 2022
Final analysis	September 2022
Final study report	February 2023

5 BACKGROUND AND RATIONALE

5.1 Background

Malaria is a major health problem in many countries of the world. Of the five parasite species that cause malaria in humans, *Plasmodium vivax* (*P. vivax*) is the second most common one, after *P. falciparum*, and has the largest geographic distribution (1). World Health Organization (WHO) estimates place the global *P. vivax* malaria burden at 7.5 million cases in 2017 (2).

Treatment strategies and malaria control are generally more effective against *P. falciparum* than *P. vivax.* Incidence of *P. falciparum* often declines faster, whereas *P. vivax* is generally slower to respond (3).

In Brazil, although the absolute incidence of malaria decreased in recent years, *P. vivax* has become the predominant malaria-causing species, with approximately 170,000 new cases in 2017 (2, 4).

P. vivax infection is characterized by the persistence of latent parasites (liver-stage hypnozoites), which cause recurrent malaria episodes months or even years after the initial infection (5). WHO's guidelines for the complete treatment of *P. vivax* malaria (radical cure) recommend treatment of the blood stage infection (causing the symptoms of the disease) and of the latent liver stage to achieve clinical cure, and to prevent relapses, onward transmission and progression to severe disease (6).

Chloroquine (CQ) is the most widely used treatment of the blood stage infection. Currently, primaquine (PQ) is the only therapy widely available for treating hepatic stages.

The main safety concern with PQ is the high risk of acute hemolytic anemia (AHA) in individuals with glucose-6-phosphate dehydrogenase (G6PD) deficiency (G6PD enzyme activity <30% of normal) (7).

G6PD deficiency is a common hereditary red blood cell disorder with an overall prevalence across malaria-endemic countries of 8.0% (interquartile range, 7.4–8.8), corresponding to approximately 350 million affected individuals. In Brazil, the prevalence of G6PD deficiency has been estimated at 4.8% (interquartile range, 3.6–6.5) (8).

More than 185 clinically relevant variants of G6PD deficiency have been reported causing a wide range of biochemical and clinical phenotypes (9). As the G6PD gene is located on the X-chromosome, males can be either deficient (hemizygotes) or normal, whereas females can be fully (homozygotes) or partially (heterozygotes) deficient, or normal. Heterozygous females have both G6PD normal and G6PD deficient red blood cells. However, because random X-inactivation (lyonisation) occurs early in embryogenesis, the ratio of deficient to normal red blood cells is highly variable between heterozygous females (10).

The risk of hemolysis after exposure to PQ depends on the dose of PQ and the degree of G6PD enzyme activity. As a result, hemolysis can be negligible and self-limited in individuals with milder deficiencies or result in potentially fatal AHA in individuals with less than 10% of normal enzyme activity (7, 11).

In order to reduce the risk of severe hemolysis, the WHO malaria treatment guidelines recommend that PQ is administered over 14 days (2). However, adherence to this prolonged regimen is frequently challenging. Since patients become rapidly asymptomatic after treatment with CQ, they often fail to complete PQ treatment (7, 12, 13). In Brazil, the recommended PQ dosing regimen for *P. vivax* is PQ 3.5 mg/kg for 7 or 14 days, in combination with CQ 25 mg/kg for 3 days. The 7-day regimen is recommended in endemic regions to ensure higher adherence with the radical cure treatment (4).

Tafenoquine (TQ) is a slowly eliminated primaquine analogue with a longer half-life (~2 weeks), allowing for a single dose regimen to provide radical cure (in combination with 3-day CQ treatment) (14, 15). This simplified dosing regimen may be a significant advance in improving the adherence issues associated with PQ and thereby improving regimen effectiveness in real-life settings.

Clinical efficacy of TQ for *P. vivax* radical cure was assessed in a seamless phase 2b and phase 3 study. Part 1 of the DETECTIVE study (Dose and Efficacy Trial Evaluating Chloroquine and Tafenoquine In Vivax Elimination) was a phase 2b, multicenter, double-blind, randomized, placebo-controlled, dose-selection study, which evaluated TQ efficacy in preventing relapse at 6 months. Patients were treated with CQ on Days 1 to 3, followed by either a single dose of TQ (50 mg, 100 mg, 300 mg, or 600 mg), or PQ 15 mg for 14 days, or placebo (54 subjects per treatment arm). Study results showed that the 300 mg dose of TQ was optimal in terms of safety and efficacy. The estimate of recurrence-free efficacy at 6 months was significantly greater in the 300 mg TQ+CQ group (89.2% [95% Confidence Interval (CI): 77%, 95%]) than in the CQ alone group (37.5% [95% CI: 23%, 52%]) (16).

Part 2 of the DETECTIVE study was a phase 3 study (NCT01376167) and aimed to investigate TQ efficacy of the TQ/CQ dose selected in the phase 2b study. Patients were treated with CQ on Days 1 to 3, followed by either a single dose of TQ 300 mg on Days 1 or 2, or PQ 15 mg for 14 days, or placebo. A total of 522 subjects were included in a 2:1:1 ratio. Treatment with TQ+CQ resulted in a significantly greater recurrence-free efficacy at 6 months (62.4% [95% CI: 55%, 69%]) compared with CQ alone (27.7% [95% CI: 20%, 36%]). In the TQ+CQ group, the risk of recurrence over 6 months was significantly reduced by 70.1% (95% CI: 59.6%, 77.8%) compared with CQ alone (17).

A second supportive phase 3 safety and efficacy study (NCT02216123) called GATHER (Global Assessment of Tafenoquine Hemolytic Risk) aimed to investigate the safety TQ/CQ versus PQ/CQ using a protocol-defined hemoglobin decrease (>3.0 g/dL or ≥30% from baseline, or to an absolute value <6.0 g/dL). Patients were treated with CQ on Days 1 to 3, followed by the randomized treatment (300 mg single dose TQ or 15 mg PQ once daily for 14 days) and matching placebo. A total of 251 patients were included in a 2:1 ratio. In this study, TQ+CQ and PQ+CQ treatments showed a comparable efficacy in all measures, including the recurrence-free efficacy at 6 months (18).

Like PQ, TQ can potentially cause drug-induced AHA in G6PD-deficient patients (19). To manage the hemolysis risk, G6PD testing must be performed before TQ initiation. TQ use must be restricted to individuals whose G6PD enzyme activity is ≥70% of normal. DETECTIVE studies, which included only patients with G6PD enzyme activity ≥70%, showed that the mean decrease in hemoglobin was 0.6% (95% CI: -8.9%, 4.4%) a week after TQ+CQ treatment, compared with 2.2% (95% CI: -6.4, 2.1) after CQ alone and 1.4% (95% CI: -5.9, 0.8) after CQ+PQ (16).

In the GATHER study the incidence of clinically relevant hemolysis was low and similar in both treatment groups in patients with G6PD enzyme activity ≥70% (2.41% for patients receiving TQ+CQ vs. 1.18% for patients receiving PQ+CQ, estimated mean difference: 1.23% [95% CI: -4.16%, 4.98%]). The majority of hemoglobin drops in both treatment groups were low (<2.0 g/dL) and of no clinical concern. No subjects required a blood transfusion. Only 1 subject in the TQ treatment group experienced a hemoglobin nadir <8.0 g/dL, corresponding to a hemoglobin drop of 20 g/dL from Baseline to Day 3 that normalized without specific medical intervention.

Additionally, aforementioned clinical trials showed that TQ was well tolerated with no evidence of increased adverse events compared with the other treatment groups and a favorable benefit: risk profile in adults and adolescents ≥16 years with G6PD levels ≥70% of normal (16).

5.2 Study Rationale

TQ has been approved by the US-Food and Drug Administration (FDA) and the Australian Therapeutics Goods Administration. TQ has been approved for use in Brazil, by the Brazilian Health Regulatory Agency (ANVISA). The municipalities of Manaus (Amazonas state) and Porto Velho (Rondônia state), with the recommendation of the Ministry of Health, through the Department of Immunization and Communicable Diseases (DEIDT), will officially adopt the use of quantitative G6PD tests before the radical cure of P. vivax (PQ or TQ) and TQ as the first choice treatment for radical cure of P. vivax in patients ≥16 years after the G6PD test. The study will only begin when the new protocol is adopted by the municipalities mentioned and the tafenoquine is approved by ANVISA.

Like PQ, TQ presents a risk of hemolysis when given to G6PD deficient patients. Current labeling mandates that TQ must only be administered to patients with ≥70% G6PD enzymatic activity. A robust, portable, quantitative point-of-care G6PD diagnostic has been developed by SD Biosensor to support the use of TQ and needs to be deployed with TQ. The SDB quantitative G6PD test has been approved for use in Brazil by ANVISA. However, the appropriate and systematic use of G6PD testing within the health system in order to identify patients at risk and the complexity of the treatment algorithm (based on G6PD enzymatic activity and patient age) will be important barriers to well-tolerated and effective radical treatment of *P. vivax* malaria and a significant challenge for malaria control programs.

The present study is designed to investigate the ability of health care facilities in these municipalities to systematically conduct G6PD testing and provide *P. vivax* patients with appropriate radical cure treatment. The key challenge for this operational feasibility study is to ensure that the study will not influence health care providers (HCPs)' drug prescription and overall therapeutic management of patients. Following training on the municipality treatment guidelines, antimalarial treatments and G6PD tests should be prescribed by HCPs in accordance with their clinical judgment and usual standard of care. The introduction of quantitative G6PD testing and TQ could be considered an intervention at the municipality level, however it will be carried out by the MoH. Therefore, the study design itself will be non-interventional and observational, and will be based on retrospective data collection using the routine Epidemiological Surveillance System for Malaria (SIVEP-Malaria), in order to not unduly influence patients' management and as such, to minimize the risk of bias.

This study will provide the with evidence on the feasibility of providing appropriate *P. vivax* radical cure following G6PD testing, at various levels of the health services, under programmatic conditions. This evidence and the insights gained will be crucial for the NMCP to decide on revisions to national malaria treatment policies in Brazil.

6 OBJECTIVES AND ENDPOINTS

6.1 Objectives

6.1.1 Primary Objective

The primary objective is to investigate whether *P. vivax* patients ≥16 years are treated with TQ in accordance with the appropriate level of G6PD enzyme activity.

6.1.2 Secondary Objectives

The secondary objectives are:

- To investigate whether *P. vivax* patients ≥6 months are treated with PQ in accordance with the appropriate level of G6PD enzyme activity.
- To describe the characteristics of patients treated with TQ or PQ.
- To measure frequency of drug-induced signs of AHA and hospitalization due to drug-induced AHA.

6.2 Endpoints

6.2.1 Primary endpoint

The primary endpoint will be the percentage of *P. vivax* patients aged ≥16 years treated or not with TQ in accordance with the appropriate level of G6PD enzyme activity. The primary endpoint will be calculated as follows:

- Among patients aged ≥16 years^:

$$\frac{\text{Appropriate use of TQ}^{*} + \text{Appropriate non-use of TQ}^{**}}{\text{Total use of TQ}^{***} + \text{Appropriate non-use of TQ}^{**}}$$

^ excluding those with who were treated for *P. vivax* malaria in the last 60 days****

* Number of patients with ≥70% G6PD activity treated with TQ
** Number of patients with <70% G6PD activity not treated with TQ
*** Number of patients treated with TQ

6.2.2 Secondary endpoints

Secondary endpoints will be the following:

- Percentage of *P. vivax* patients aged ≥6 months treated or not with daily PQ in accordance with the appropriate level of G6PD enzyme activity:

  Among patients aged ≥6 months:

$$\frac{\text{Appropriate use of daily PQ}^{*} + \text{Appropriate non-use of daily PQ}^{**}}{\text{Total use of PQ}^{***} + \text{Appropriate non-use of daily PQ}^{**}}$$

* Number of patients with ≥30% G6PD activity treated with daily PQ
** Number of patients with <30% G6PD activity not treated with daily PQ
*** Number of patients treated with daily PQ

- Description of the characteristics of patients treated with TQ or with PQ
- Frequency of confirmed drug-induced AHA and hospitalization due to drug-induced AHA

7 HYPOTHESIS

The systematic use of a quantitative G6PD test to inform treatment decision ensures that patients receive appropriate treatment based on municipalities' guidelines while also being able to reduce the risk of acute hemolytic anemia associated with the use radical cure drugs in patients with inadequate levels of G6PD enzymatic activity after *P.vivax* malaria diagnosis.

8 STUDY DESIGN

This study was designed as a study about an implementation. Studies about implementation requires the involvement of several stakeholders and disciplines to address the complex challenges faced when a tool is put into practice in the health sector. The promotion of collaborative ties between the main stakeholders involved in the generation of policies, program management and research is essential, which is why the present study has the participation of the Federal Government, represented here by the Ministry of Health, through the DEIDT.

Studies about implementation enable to assess contextual factors in the real world, usually not captured by other common research methods. In this sense, these methods provide more solid bases for contextual and evidence-based decision making, leveraging, and making the theoretical effects of the evaluated tools more efficient. In this study design, issues that can only be raised by professionals with a direct link to the target population and who experience the reality of the field, as well as populations' customs and behaviors, are considered in the implementation context. Such questions are included among the variables to be evaluated in this type of study.

Embedded in the real world, the research about implementation is also a powerful tool for capturing and analyzing information in real time, enabling performance assessment, and contributing to the strengthening of health systems, which is particularly important when new intervention tools are integrated in the health systems at the national level. These interventions often work in small-scale pilot studies, but do not live up to expectations when implemented in regional or national strategies or even fail to be transferred from one country to another because of contextual differences.

Studies like the one being suggested here are crucial to improve the understanding of the challenges to be faced when confronting the real world, expanding, and deepening the understanding of these factors and how they impact implementation.

This is a non-interventional, observational study conducted in Brazil on patients with *P. vivax* malaria. The study will be conducted in the municipalities of Manaus (Amazonas State) and Porto Velho (Rondônia State).

Both municipalities will officially define TQ to be the first choice for radical cure of *P. vivax* in patients aged ≥16 years with *P. vivax*-confirmed malaria, after G6PD testing. As part of treatment policy in the municipalities of Manaus and Porto Velho, patients will be asked to return for a follow-up visit at Day 5 to confirm blood-stage parasite clearance and to detect any signs of AHA.

G6PD tests and TQ will be supplied to health care facilities by the Municipal Health Authorities using the usual supply route for drugs and diagnostics. PQ and other anti-malarial drugs are already available in Brazil.

Designated staff at health care facilities will be trained on the quantitative G6PD test procedure and the radical cure treatment algorithm by the Principal Investigator (PI) staff and the municipality authorities using educational materials developed by the sponsor in collaboration with the Brazilian Ministry of Health (NMCP). These materials are intended for roll-out during scale-up of TQ introduction.

The design of the study is based on secondary use of data collected routinely for all patients with malaria in the SIVEP-Malaria by the NMCP. Information of patients with malaria is routinely collected in the SIVEP forms by HCPs and subsequently entered in the SIVEP database by the municipality staff. In accordance with the NMCP, the SIVEP form has been adapted to collect additional information about G6PD testing, TQ treatment and signs of hemolysis.

Within the study period, and on a regular basis, retrospective data from all patients will be entered into a new, study database by the municipality staff. Relevant data will be automatically exported, on a weekly basis, to the SIVEP database. The project staff will only have access to de-identified data in line with the access rights that will be attributed to them in the system. Only municipal staff will have access to identified patient data.

Although treatment policy will include a follow-up visit at Day 5, a proportion of patients may not return for this follow-up visit. In addition to the data collected from the SIVEP forms, PI staff will request the two referral hospitals likely to see all hospitalizations for AHA to regularly screen hospital electronic admission records for patients presenting signs of AHA (renal failure, jaundice, blood transfusion, malaria). All identified cases will be investigated using hospital records and SIVEP forms. Information about confirmed drug induced AHA will be linked with patient data recorded in the database by the municipality staff. The screening process for AHA cases will be fully described into an AHA screening plan. All AHA cases will be also reported to ANVISA in accordance with national procedures.

Finally, the additional costs of implementing G6PD testing and TQ will be collected alongside the study in the health care facilities (See Section 10.2).

Only de-identified data will be used for statistical analyses. Due to the retrospective and de-identified nature of data reviewed by the study team and the very low risk attributed to the study for patients, patient informed consent, for the secondary use of their data, will be recorded by the HCP in the SIVEP form (See Section 14.4).

As the study is based on retrospective data collection, G6PD testing and treatment of patients with TQ or PQ will be conducted in line with the treatment policy, i.e., independent from the study. Tests and treatments will be prescribed by physicians in accordance with their clinical judgment and the prescribing information for TQ and PQ.

The study will be conducted in a phased manner (Figure 1):

- 1st phase (approximately 3 months): Training and provision of G6PD tests and TQ will be initially limited to higher and medium-level health facilities (referral hospitals, hospitals, emergency care units,polyclinics). Data will be collected for *P. vivax* patients managed at these facilities.

  An interim analysis will be conducted after data from 600 *P. vivax* patients ≥16 years are collected in the study database, in order to decide whether the study could be extended to lower-level health facilities. The decision will be made by a Independent Study Oversight Committee (ISOC) based on the results of the interim analysis and any other factors as deemed appropriate (e.g., information on training, stocks and consumption of G6PD readers, test strips, TQ and PQ). The organization and decision criteria of the ISOC will be defined in a charter (See Section 14.3).

  If the interim results of the 1st phase are deemed unsatisfactory (e.g., difficulty of applying the treatment algorithm, incidence of drug-induced AHA), the ISOC may decide not to extend the study to lower-level health facilities until improvements are made in the education program and/or additional HCP support is implemented. Additional interim analyses may be conducted as appropriate.

- 2nd phase (approximately 9 months): If approved by the ISOC, the study will be extended to lower-level health facilities (basic health units, basic family health units and primary care) and to additional higher and medium-level health facilities. After training of the staff, G6PD tests and TQ will be supplied to these health care facilities.

During this 2nd phase, data will continue to be collected for *P. vivax* patients managed by the higher and medium-level health facilities of the 1st phase.

An additional interim analysis will be conducted after data from 600 *P. vivax* patients ≥16 years from lower-level facilities are collected in the study database (approximately 3 months after the beginning of the 2nd phase).

The study will continue while interim analyses are being conducted.

Final results will be reviewed and validated by the ISOC.

It is anticipated that it will take approximately 15-months to carry out the study.

Figure 1: TRuST Study Flow

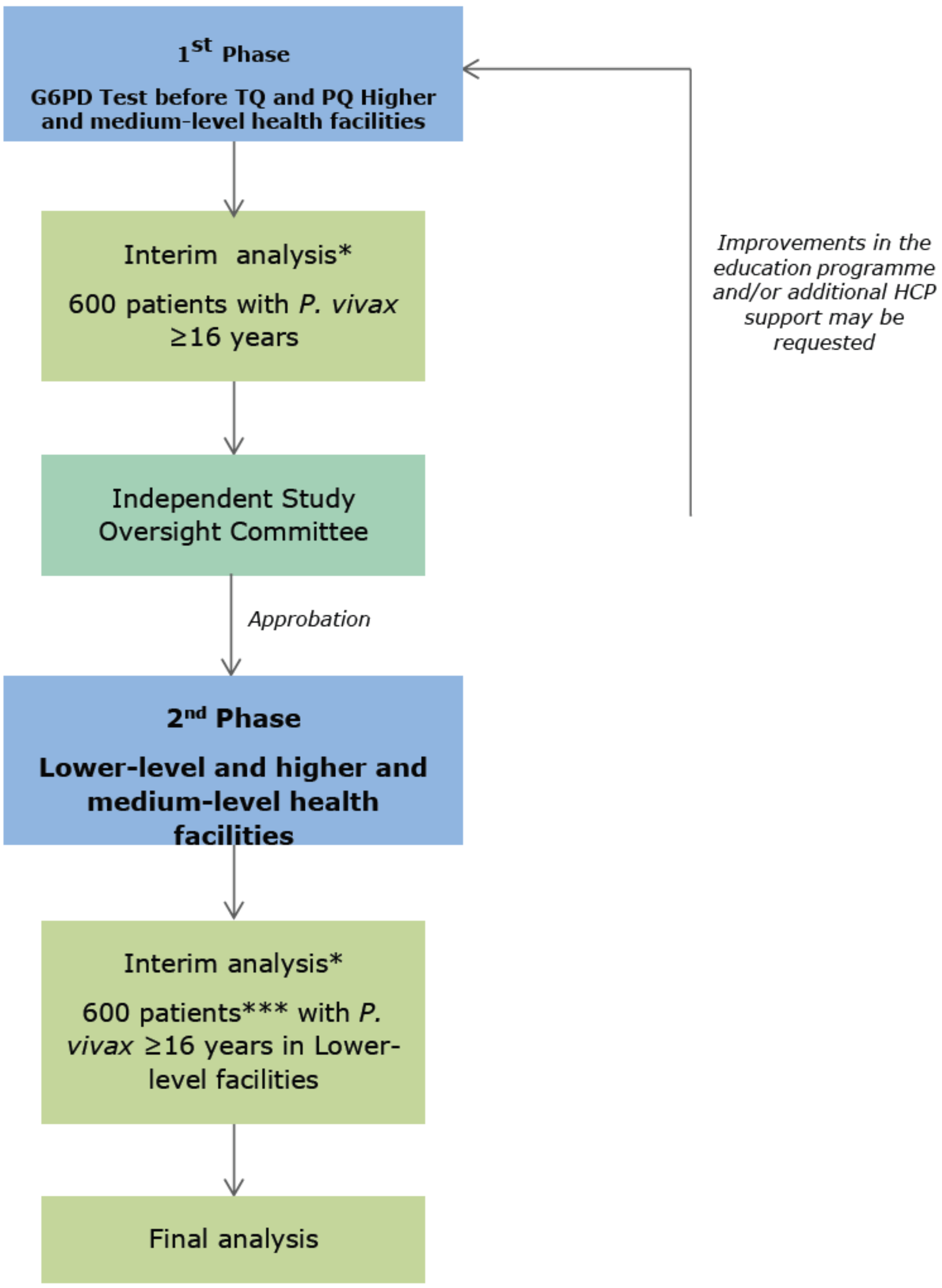

*While interim analyses are being conducted, the study will continue, and health care facilities will continue to access G6PD testing and TQ

** excluding those who were treated for *P. vivax* malaria in the last 60 day

9 STUDY POPULATION

9.1 Health Care Facilities

The study will be conducted in Brazil in the municipalities of Manaus (Amazonas State) and Porto Velho (Rondônia State).

The relevant staff at health care facilities will be trained by the PI staff and the municipality authorities using educational materials (intended for roll-out during scale-up of TQ introduction) on the need for and use of G6PD testing, the treatment algorithm based on G6PD activity levels, adverse events of TQ and PQ, identifying, reporting and managing adverse events to ANVISA and surveillance (completion of revised SIVEP form). Training logs will be maintained.

TQ, G6PD readers and test strips will be supplied to health care facilities by the Municipal Health Authorities after staff training is completed.

During the 1st phase, the training and provision of TQ and G6PD tests will be limited to the same 10 higher and medium-level health facilities (referral hospitals, hospitals, emergency care units, polyclinics). Duringthe 1st phase, data will be collected only for *P. vivax* patients managed at these higher-level health facilities.

If approved by the ISOC, the 2nd phase will be initiated in a phased manner and the training and provision of TQ and G6PD tests will be extended to approximately 100 lower-level health facilities (basic health units, basic family health units and primary care that are less than 24h away from emergency care) and the remaining higher and medium-level health facilities. During the 2nd phase, data will becollected for *P. vivax* patients managed at these health facilities and will continue to be collected for
P. vivax patients managed by the higher and medium-level health facilities of the 1st phase.

9.2 Patients

9.2.1 Inclusion Criteria

A patient who has consented/assented must meet the following criteria to be eligible for the study:

- Diagnosed with mono-species *P. vivax* malaria (parasitologically confirmed through microscopy or rapid diagnostic test) and older than 6 months of age.
- Diagnosed with *P. vivax / P. falciparum* malaria co-infection (parasitologically confirmed through microscopy or rapid diagnostic test) and older than 6 months of age.
- Diagnosed negative for *P. vivax* mono-species malaria or *P. vivax / P. falciparum* malaria co-infection but had a positive diagnosis in the past.
-

9.2.2 Exclusion Criteria

None.

10 MEDICINAL PRODUCTS AND G6PD DIAGNOSTIC TEST

Tafenoquine 300 mg single-dose, is an 8-aminoquinoline anti-malarial drug indicated for the radical cure (prevention of relapse) of *P. vivax* malaria. Tafenoquine is supplied as 150 mg tablets.

Primaquine is an 8-aminoquinoline anti-malarial drug indicated for the radical cure (prevention of relapse) of *P. vivax* malaria. Primaquine is supplied as 7.5mg and 15 mg tablets.

The SD Biosensor STANDARD G6PD test is a point-of-care, quantitative glucose-6-phosphate dehydrogenase (G6PD) enzyme activity analyzer. It is a colorimetric test and provides the G6PD activity as a ratio to hemoglobin (U/g Hb). It is a rapid and simple test procedure providing results in 5 minutes. The reader can be held with one hand and is battery-operated for on-site examination. The strips need to be stored at room temperature: 2-30 °C / 36-86℉

The TQ, the SD Biosensor Standard G6PD reader and the strips will be supplied by MMV. These will be distributed to health units through the usual system of supplying medicines and supplies in the municipalities. The distribution will only happen after the TQ is approved by ANVISA and after the teams of the health units are trained in the new treatment protocol. Primaquine and other malaria drugs are already available in Brazil.

Tafenoquine and SD Biosensor Standard G6PD test reader and strips will be supplied by the Tropical Medicine Foundation Doctor Heitor Vieira (FMT-HVD) and CEPEM – Centro de Pesquisa em Medicina Tropical to the Municipal Health Authorities for onward distribution to health care facilities. Primaquine and other anti-malarial drugs are already available in Brazil.

11 STUDY ASSESSMENTS

11.1 Patient Data

The following data will be retrospectively collected from the SIVEP Form

Day 1 (initial presentation)

- Patient characteristics
    - Socio-demographics: age and date of birth, gender, ethnicity, principal activity
    - **Weight**
    - Phone number
    - **Consent / assent**
    - Prior malaria infection: *P. vivax* in previous 60 days; *P. falciparum* malaria in previous 40 days
    - Malaria diagnosis: date of first symptoms, diagnostic test (date, type of test, test results), parasitemia
    - Pregnancy status
    - If the woman is breastfeeding for less than 1 month
    - If the patient received a blood transfusion less than 60 days ago
    - **Hemoglobin level**
- **G6PD enzyme activity**
    - **Quantitative G6PD test (test performed or not, date of the test, G6PD test result)**
- Malaria treatment
-
    - Treatment administered: anti-malarial drugs administered (*TQ*, PQ, CQ, other), start date, intended treatment duration
- Clinic characteristics
    - Municipality (name and code)
    - Health facility (name and code)
    - HCP (name and code)

Day 5 (follow-up visit)

- Safety Assessments
    - Signs of hemolysis (jaundice, dark urine, other)

In case of confirmed drug-induced AHAs detected at the 2 referral hospitals, the following additional data will be collected from patient medical records and HCP training logs, and linked with the data collected in the SIVEP form:

- Time to onset from anti-malarial treatment
- Hemoglobin level
- AHA signs and symptoms
- AHA Treatment
- Hospitalization (hospitalization date, discharge date)
- Outcome
- Training of HCP(s) who performed the G6PD test and provided the anti-malarial treatment (date of training, results of competency assessment)

11.2 Cost Data

The following data about health facilities will be collected:

- Salaries, tax, travel pay for staff members in contact with patient, including any administrative staff
- Additional time needed for G6PD testing and related training and quality assurance
- Consumables (i.e., G6PD test strips, drug costs)
- Equipment costs (i.e., biosensor, motorcycle)

12 SAFETY REPORTING

Confirmed drug-induced AHA and hospitalization due to drug-induced AHA will be captured in the study database.

Due to the retrospective design of the study, the reporting of safety events to the sponsor is not solicited for this study and the submission of suspected adverse drug reactions in the form of individual case safety reports is not required.

All AHA cases collected for the study will be recorded in the study database and summarized in the study report.

HCPs will be encouraged to report all adverse events, including all AHA cases, to ANVISA VigiMed system in accordance with national procedures.

13 STATISTICAL METHODS

13.1 Study Size

The study size calculation was based on the primary endpoint of the study (percentage of patients with *P. vivax* aged ≥16 years with ≥70% G6PD activity treated with TQ and with <70% G6PD activity not treated with TQ among all patients treated with TQ and patients with <70% G6PD activity not treated with TQ). The study size was determined to ensure a satisfactory precision of this percentage.

The precision (e) of the 95% CI of a percentage (i.e., half of the width of the CI) is determined using the following formula:

$$e \quad 1.96 \times *\overline{\frac{(1 \quad p)}{n}}$$

where p is the percentage and n the study size.

The following table presents the precision and the corresponding 2-sided 95% CI for a percentage of patients with *P. vivax* aged≥16 years with ≥70% G6PD activity treated with TQ or with <70% G6PD activity not treated with TQ varying from 80% to 99% and a study size of 400 to 5000 patients with *P. vivax* treated with TQ or with <70% G6PD activity not treated with TQ.

Table 2 Precision and 95%CI obtained for percentages from 80% to 99% with study sizes of 400, 500, 1000, 2000 and 5000

	**Percentage**									
	**80%**		**90%**		**95%**		**97.5%**		**99%**	
**N**	**e (%)**	**95% CI**	**e (%)**	**95% CI**	**e (%)**	**95% CI**	**e (%)**	**95% CI**	**e (%)**	**95% CI**
**400**	3.9	76.1;83.9	2.9	87.1;92.9	2.1	92.9;97.1	1.5	96.0;99.0	1.0	98.0;100.0
**500**	3.5	76.5;83.5	2.6	87.4;92.6	1.9	93.1;96.9	1.4	96.1;98.9	0.9	98.1;99.9
**1000**	2.5	77.5;82.5	1.9	88.1;91.9	1.4	93.6;96.4	1.0	96.5;98.5	0.6	98.4;99.6
**2000**	1.8	78.2;81.8	1.3	88.7;91.3	1.0	94.0;96.0	0.7	96.8;98.2	0.4	98.6;99.4
**5000**	1.1	78.9;81.1	0.8	89.2;90.8	0.6	94.4;95.6	0.4	97.1;97.9	0.3	98.7;99.3

e: precision of the 95% CI.

For example, with 500 patients, the 95% CI for a percentage of 97.5% is (96.1%, 98.9%), and the precision 1.4%. With 400 patients, the 95% CI for a percentage of 97.5% is (96.0%, 99.0%), and the precision 1.5%.

Interim analyses

For interim analyses, 500 patients were considered appropriate to estimate the primary endpoint with an acceptable precision. Assuming that *P. vivax* patients treated with TQ and patients with <70% G6PD activity not treated with TQ will represent approximately 85% of *P. vivax* patients ≥16 years, interim analyses will be conducted after data from 600 *P. vivax* patients ≥16 years who have not been treated for P. vivax malaria in the last 60 days are collected in the study database to enable an assessment of the primary endpoint based on 500 patients.

Overall study size

In 2017, 16,294 and 3,517 *P. vivax* malaria cases were reported in the municipalities of Manaus and Porto Velho, respectively. Overall, 16,000 *P. vivax* patients are expected to be included in the study (approximately 12,000 in higher and medium level health facilities and 6,000 in lower level health facilities). Assuming that *P. vivax* patients ≥16 years will represent approximately 60% of *P. vivax* patients and

that *P. vivax* patients treated with TQ and *P. vivax* patients with <70% G6PD activity not treated with TQ will represent approximately 85% of *P. vivax* patients ≥16 years who have not been treated for P. vivax malaria in the last 60 days, the primary endpoint will be assessed in approximately 8,000 patients. Only de-identified data will be used for statistical analyses.

13.2 Statistical Analyses

A formal statistical analysis plan (SAP) that will provide details of all analyses and presentation of study data will be approved prior to database lock. All analyses will be carried out using SAS® version 9.4 or higher (SAS® Institute, Cary, NC).

13.2.1 General Considerations

Continuous variables will be described by their mean, standard deviation, median, quartiles 1 and 3, extreme values (minimum and maximum) and the number of missing data. Categorical variables will be described by the total and percentage of each response and the number of missing data. Missing data will not be included in the calculation of percentages.

95% CIs will be provided when relevant. Figures will also be performed when appropriate.

Analysis will be purely descriptive (no tests performed).

13.2.2 Analyzed Populations

All patients who meet the selection criteria and consent to their de-identified data being used in the study will be considered in the Analysis population.

Analysis will be performed overall and by subgroups (patients treated with TQ / patients treated with PQ, patients from higher and medium-level facilities / patients from lower-level facilities, age classes, pregnant women). Additional subgroups of interest (to be defined prospectively) may also be examined, as deemed appropriate.

13.2.3 Data Analysis

Patient disposition

A summary of all patients included in the study database, as well as patients of the Analysis Population will be provided (including reasons for exclusion from the Analysis Population).

Patient characteristics

Patient characteristics (socio-demographics characteristics, malaria history, malaria diagnosis, pregnancy, hemoglobin levels) will be presented using descriptive summary statistics on the Analysis Population.

G6PD test

The number and percentage of *P. vivax* patients tested for G6PD deficiency will be summarized. The distribution of G6PD test results will be described. Among the *P. vivax* patients treated with TQ, the number and percentage of patients with ≥70% G6PD activity, with <70% G6PD activity and with no G6PD test will be described. Among the *P. vivax* patients treated with PQ, the number and percentage of patients with ≥30% G6PD activity, with <30% G6PD activity and with no G6PD test will be described. The corresponding value of the % cut-off for G6PD activity, in U g/Hb (Units of G6PD enzyme activity per gram of Hb) will be established based on the ongoing G6PD test clinical validation studies.

Treatments

Anti-malarial drug administered and intended treatment duration will be presented using descriptive summary statistics.

Costs

Additional costs of implementing G6PD testing and TQ / PQ per *P. vivax* malaria episode by health care provider will be calculated.

Primary analyses

The number and percentage of *P. vivax* patients aged ≥16 years treated or not with TQ in accordance with the appropriate level of G6PD enzyme activity will be summarized using descriptive statistics for the analysis population and in the subgroups of interest.

Secondary analyses

The number and percentage of *P. vivax* patients aged ≥6 months treated or not with daily PQ in accordance with the appropriate level of G6PD enzyme activity will be summarized using descriptive statistics for the analysis population and in the subgroups of interest.

Patient characteristics (age, gender, ethnicity, weight, malaria history, malaria diagnosis, hemoglobin, pregnancies, G6PD activity) of patients treated with TQ and of patients treated with PQ will be presented using descriptive statistics.

Confirmed drug-induced AHA will be presented overall, by anti-malarial drug received, and by G6PD test results, using descriptive summary statistics: number and percentage of patients who experienced at least one AHA, number of AHA in total and per patient, hemoglobin level, AHA symptoms, treatments, hospitalizations and outcomes, training of HCP(s) who performed the G6PD test and provided the anti-malarial treatment.

13.2.4 Interim Analyses

A first interim analysis will be conducted after data from 600 *P. vivax* patients ≥16 years who have not been treated for P. vivax malaria in the last 60 days are collected in higher and medium-level health facilities(all patient data collected from higher and medium-level health facilities at the data cut-off will be analyzed).

An additional interim analysis will be conducted after data from 600 *P. vivax* patients ≥16 years who have not been treated for P. vivax malaria in the last 60 days are collected in higher and medium - level facilities (all patient data collected from lower-level health facilities at the data cut-off will be analyzed).

13.2.5 Final Analyses

Based on epidemiological trends, about 16,000 *P. vivax* cases are likely to be diagnosed and treated within the study period and about 8,000 patients are likely to be treated with tafenoquine. A final analysis will be conducted at the end of the study.

13.3 Bias of the Study

Three types of bias are usually considered for this type of study: selection, information, and confounding bias.

13.3.1 Selection Bias

Selection bias is a distortion of evidence or data that arises from the way that the data are collected.

In order to limit bias in the selection of patients, data from all *P. vivax* patients, regardless of treatment, health status, or other considerations will be included in the analysis.

13.3.2 Information Bias

Information bias results from systematic differences in the way data on exposure or outcome are obtained from the various study groups.

This may mean that patients are assigned to the wrong treatment or outcome category, leading to an incorrect estimate of the association between exposure and outcome. This could occur in this study if the information recorded in the revised SIVEP forms is inaccurate and incomplete or if HCPs do not report certain types of events of potential interest because they feel they are not related to the safety of the medication.

Designated personnel will participate in a training program conducted by the PI staff and the municipality authorities that will encourage consistency of process and procedures at the health care facilities and ensure collection of high-quality data in the revised SIVEP form.

13.3.3 Confounding Bias

Confounding bias occurs when the effects of a treatment or the exposition effect of the disease vary by the presence/level of another factor (effect modifier).

Effect modifiers may be countered by using stratification in the statistical analysis: several subgroup analyses will therefore be conducted on the endpoints (See Section 12.2.2).

14 DATA HANDLING

All data collected in the context of this study will be stored and evaluated in accordance with regulatory requirements and applicable guidance for electronic records.

During the one-year duration of the study, all the data collected under the protocols established by the Brazilian Ministry of Health, will be entered by the municipal staff in to the updated SIVEP system. The routine data collection and entry process will be respected and identified patient data will only be seen by the municipal staff.

Data will be collected and subsequently entered by the municipal staff into the SIVEP database at three distinct time points:

- On collection of blood for examination (minimal essential information)
- When a positive malaria diagnosis has been made (more extensive information)
- On Day 5, when the patient returns for a follow-up blood examination (information about adverse events).

Once a week, relevant data of patients who consented to share their unidentified data, will be automatically exported from the SIVEP database to the study database. This approach will ensure that patient confidentiality is fully respected as the data is entered by the municipal staff. Moreover, the system settings and access rights, will ensure that identified patient data is only seen by municipal staff. Study staff will only have access to de-identified data. A unique study ID number will be attributed to each patient in the study database. This approach will avoid the need for duplicate entries and also help allow the NCMP to assess the ease of capturing additional information.

The study team will perform weekly quality control of the data in addition to automated checks to detect and flag missing or inconsistent data in the study database. In the event of missing or inconsistent data, queries will be issued and managed by the PI staff in collaboration with the municipality staff.

Only authorized personnel will have access to the study database. Information security is guaranteed by Transport Layer Security (TLS) protocols.

Hospital admissions due to hemolytic anemia will be linked with the patient's corresponding SIVEP forms using an algorithm based on probabilistic linkage with the support of the relevant municipal staff.

Specific processes for quality control and data management throughout the study will be documented in the Data Management Plan and relevant SOPs.

Data review meetings will be conducted before interim and final analyses in order to review the data collected in the study database. After the study database is declared clean and is released to the statistician, a final copy of the database will be stored by the sponsor.

15 ETHICAL CONDUCT OF THE STUDY

15.1 Ethical Considerations

The study will be conducted in compliance with International Society for Pharmacoepidemiology Good Pharmacoepidemiology Practice (ISPE GPP) guidelines[20], the ethical principles arising from the Declaration of Helsinki revised in 2013[21] and all current local regulations.

Study personnel involved in conducting this study will be qualified by education, training, and experience to perform their respective task(s).

This study will not use the services of study personnel where sanctions have been invoked or where there has been scientific misconduct or fraud (e.g., loss of medical licensure, debarment).

15.2 Institutional Review Board/Independent Ethics Committee

This study is non-interventional. The drug used to treat the patients is based on clinical judgment alone, and, as such, does not come under applicable laws and regulation on clinical trials. No specific examinations or laboratory tests are to be performed above and beyond those usually undertaken by the physician, and no additional visits are required for study purposes.

A written and dated approval/favorable opinion from the relevant IRB/IEC for the protocol and any relevant supporting information will be obtained before study initiation. The sponsor should provide the relevant IRB/IEC with reports, updates and other information (e.g., amendments and administrative letters) according to regulatory requirements or institution procedures.

15.3 Independent Study Oversight Committee

The role of the Independent Study Oversight Committee will be to provide guidance on study procedures, review and validate study results (interim and final) and recommend on or against an extension of the study to lower-level health facilities.

The Independent Study Oversight Committee will be constituted of the Ministry of Health, selected local country Key Opinion Leaders and experts. This committee will meet at regular, pre-defined time-points.

To ensure independence and transparency, committee members who have any commercial, financial or institutional interest in any outcome of the study may only participate in Committee meetings as invited specialists. Specialists may participate in discussions but cannot be involved in the decision-making process.

The roles, responsibilities, procedures and decision criteria of the Study Oversight Committee will be defined in a charter.

15.4 Benefits

From the patients' perspective, participation in the study is voluntary. Patients who consent to the inclusion of their data in the study will not benefit directly and will not receive any payment for participating. However, their anonymized data will contribute to the evidence base that will support the Ministry of Health to improve Brazilian treatment guidelines for vivax malaria. This will enable Brazilian patients to receive more effective and appropriate treatment.

From a public health perspective, the study should also result in a reduced risk of relapses and incidence of vivax. This leads to a reduction in transmission benefiting the general population in the

regions most affected by the disease. Also, the health professionals involved in the study will receive training by a specialized team including on how to perform a G6PD test, use the test results to select the most appropriate treatment, counsel patients and detect signs and symptoms of hemolysis. After the trainings, health professionals will be better able to provide services of higher quality. Moreover, patients with G6PD deficiency can be accurately diagnosed at any level of the health service, which may prevent health problems caused by the inadvertent prescription of primaquine and tafenoquine.

15.5 Risks

The main risk involved in TRuST is the unintentional disclosure of identifiable patient data. In order to minimize this risk, information that can identify the patient will be pseudonymized and researchers will be reminded to ensure non-violation, protection, integrity, confidentiality, and privacy of data. Patients will be guaranteed that their information will be not used in a moral, social, cultural, economic or financial way to jeopardize them. Professionals involved in entering the data in the database will be reminded of the importance to keep the confidentiality of the data and not to use the information for purposes other than TRuST. Moreover, only medical records from patients with diagnostic of malaria P.vivax that reported AHA symptoms will be consulted, exclusively during the time the study is in place, and just to collect information that is relevant for TRuST. Also, the integrity of all documents will be respected.

In addition, there may be interference in the routine of health professionals. To mitigate this, the study will carefully plan and conduct the training activities at different times and in smaller classes to minimize any disruption on the provision of health services.

Health professionals and researchers involved will be reminded to respect the cultural, social, moral, religious and ethical values, as well as the habits and customs of the participants, and will commit themselves to ensure that there is no conflict of interest between the researcher and the participants or sponsor of the project.

15.6 Informed Consent

Considering TRuST is aiming to evaluate, in real life conditions, the feasibility of adopting new tools to treat *P. vivax* malaria approved by ANVISA, the use of a traditional written informed consent/assent form (WICF) can introduce bias to the study results. Moreover, the risks associated with TRuST procedures are low and the potential for participants to be harmed is unlikely. As advised by CONEP, the National Ethics Committee in Brazil, patients will be informed about the study details, including its risks and benefits, and will be explicitly asked for permission to use their de-identified data for the analysis. Patient decision will be recorded in the SIVEP form.

HCP will explain the TRuST study to the patient (following confirmation of vivax malaria) using a leaflet and a comic book. The HCP will focus, in particular, on the reason for the G6PD test, the treatment algorithm, how the data will be used and the reason for the study.

↓

HCP to record the patient's decision (yes/no) in the SIVEP form to have their data analyzed.

↓

Patient will be given a comic book explaining the treatment tools, the study, and with contact details of study personnel in case of further questions (e.g. withdraw of consent).

15.7 Data Protection

Patients' personal data and investigator's personal data shall be treated in anonymity, complying with the Brazilian local applicable data protection laws and regulations.

Patient names or other information (i.e., date of birth) that identifies them will not be available for the study staff for analysis purposes. Patient data will be coded and rendered indirectly identifiable (de-identified) using unique study ID number in the study database.

15.8 Adherence to the Protocol

The study must be conducted as described in the approved protocol, except for an emergency situation in which proper care for the safety of the patient requires intervention. Any significant deviation from the protocol must be reported immediately to the sponsor and to the relevant IRB/IEC.

15.9 Protocol Amendment

Any change to this protocol will be documented in a protocol amendment, issued by the sponsor.

Amendments will be submitted for consideration to the approving IRB/IECs and/or Regulatory Authority, as applicable.

15.10 Retention of Patient Records

The Sponsor will maintain the data collected (study database) for at least 5 years.

15.11 Confidentiality

The information in this and related documents from the study sponsor includes trade secrets and commercial information that are confidential and may not be disclosed, unless such disclosure is required by national or other laws or regulations. In any event, persons to whom the information is disclosed must be informed that the information is confidential and may not be further disclosed by them.

Individual patient medical information obtained as a result of this study is considered confidential, and disclosure to third parties, other than those noted below, is prohibited. Such medical information may be given to the patient's personal physician or to other appropriate medical personnelresponsible for the patient's welfare.

Data generated as a result of this study are to be available for inspection on request of the sponsor, the sponsor's representatives, the relevant IRB/IEC, or regulatory agency.

16 PLANS FOR DISSEMINATING AND COMMUNICATING STUDY REPORT

This study is intended for publication, even if terminated prematurely. Publications may include any or all the following: posting of a synopsis online, abstracts and/or presentations at scientific conferences, or publications of full manuscripts.

Authorship credit will follow the guidelines established by the International Committee of Medical Journals Editors[22] and, as such, should be based on the following criteria 1) substantial contributions to the conception or design of the work; or the acquisition, analysis, or interpretation of data for the work; 2) drafting the work or revising it critically for important intellectual content; 3) final approval of the version to be published and 4) agreement to be accountable for all aspects of the work in ensuring that questions related to the accuracy or integrity of any part of the work are appropriately investigated and resolved. All authors must meet all above criteria. Although publication planning may begin before conducting the study, final decisions on authorship and the order of authors' names will be made based on participation and actual contributions to the study and writing, as discussed above.

17 REFERENCES

1. Gething PW, Elyazar IR, Moyes CL, Smith DL, Battle KE, Guerra CA, et al. A long neglected world malaria map: Plasmodium vivax endemicity in 2010. PLoS neglected tropical diseases. 2012;6(9):e1814.

2. WHO. World Malaria Report 2018. Geneva: World Health Organization, 2018.

3. Howes RE, Battle KE, Mendis KN, Smith DL, Cibulskis RE, Baird JK, et al. Global Epidemiology of Plasmodium vivax. The American journal of tropical medicine and hygiene. 2016;95(6_Suppl):15-34.

4. Siqueira AM, Mesones-Lapouble O, Marchesini P, Sampaio VS, Brasil P, Tauil PL, et al. Plasmodium vivax Landscape in Brazil: Scenario and Challenges. The American journal of tropical medicine and hygiene. 2016;95(6 Suppl):87-96.

5. Imwong M, Snounou G, Pukrittayakamee S, Tanomsing N, Kim JR, Nandy A, et al. Relapses of Plasmodium vivax infection usually result from activation of heterologous hypnozoites. The Journal of infectious diseases. 2007;195(7):927-33.

6. WHO. Control and elimination of plasmodium vivax malaria. A technical brief. World Health Organization, 2015.

7. John GK, Douglas NM, von Seidlein L, Nosten F, Baird JK, White NJ, et al. Primaquine radical cure of Plasmodium vivax: a critical review of the literature. Malaria journal. 2012;11(1):280.

8. Howes RE, Piel FB, Patil AP, Nyangiri OA, Gething PW, Dewi M, et al. G6PD deficiency prevalence and estimates of affected populations in malaria endemic countries: a geostatistical model-based map. PLoS medicine. 2012;9(11):e1001339.

9. Minucci A, Moradkhani K, Hwang MJ, Zuppi C, Giardina B, Capoluongo E. Glucose-6-phosphate dehydrogenase (G6PD) mutations database: review of the "old" and update of the new mutations. Blood Cells Mol Dis. 2012;48(3):154-65.

10. Cappellini MD, Fiorelli G. Glucose-6-phosphate dehydrogenase deficiency. Lancet (London, England). 2008;371(9606):64-74.

11. Ashley EA, Recht J, White NJ. Primaquine: the risks and the benefits. Malaria journal. 2014;13:418.

12. Galappaththy GNL, Tharyan P, Kirubakaran R. Primaquine for preventing relapse in people with Plasmodium vivax malaria treated with chloroquine. Cochrane Database of Systematic Reviews. 2013(10).

13. Douglas NM, Poespoprodjo JR, Patriani D, Malloy MJ, Kenangalem E, Sugiarto P, et al. Unsupervised primaquine for the treatment of Plasmodium vivax malaria relapses in southern Papua: A hospital-based cohort study. 2017;14(8):e1002379.

14. Rajapakse S, Rodrigo C, Fernando SD. Tafenoquine for preventing relapse in people with Plasmodium vivax malaria. The Cochrane database of systematic reviews. 2015(4):Cd010458.

15. Ebstie YA, Abay SM, Tadesse WT, Ejigu DA. Tafenoquine and its potential in the treatment and relapse prevention of Plasmodium vivax malaria: the evidence to date. Drug design, development and therapy. 2016;10:2387-99.

16. Llanos-Cuentas A, Lacerda MV, Rueangweerayut R, Krudsood S, Gupta SK, Kochar SK, et al. Tafenoquine plus chloroquine for the treatment and relapse prevention of Plasmodium vivax malaria (DETECTIVE): a multicentre, double-blind, randomised, phase 2b dose-selection study. Lancet (London, England). 2014;383(9922):1049-58.

17. Lacerda MVG, A. L-C, Krudsood S, Lon C, Saunders DL, Mohammed R, et al. Single-dose Tafenoquine for Relapse Prevention in Plasmodium vivax Malaria. NEJM, in press.

18. Llanos-Cuentas A, Lacerda MVG, Hien TT, Vélez ID, Namaik-larp C, Chu CS, et al. Tafenoquine vs. Primaquine to prevent relapse in Plasmodium vivax Malaria. NEJM, in press.

19. Rueangweerayut R, Bancone G, Harrell EJ, Beelen AP, Kongpatanakul S, Mohrle JJ, et al. Hemolytic Potential of Tafenoquine in Female Volunteers Heterozygous for Glucose-6-Phosphate Dehydrogenase (G6PD) Deficiency (G6PD Mahidol Variant) versus G6PD-Normal Volunteers. The American journal of tropical medicine and hygiene. 2017;97(3):702-11.

20. International Society for Pharmacoepidemiology (ISPE). Guidelines for Good Pharmacoepidemiology Practices (GPP). Revised June 2015 https://www.pharmacoepi.org/resources/guidelines_08027.cfm

21. Word Medical Association. Declaration of Helsinki. October 2013. http://www.wma.net/

22. ICMJE. Recommendations for the Conduct, Reporting, Editing, and Publication of Scholarly Work in Medical Journals. December 2016. http://www.icmje.org/icmje-recommendations.pdf

Statistical Analysis Plan

Protocol number: MMV_TQ_18_02

TRuST Study

Operational Feasibility of Appropriate Plasmodium Vivax Radical Cure with Tafenoquine or Primaquine after Quantitative G6PD Testing in Brazil

SAP for the final analysis

Prepared by:

ICON plc

[redacted]

SPONSOR:

Medicines for Malaria Venture

[redacted]

National Malaria Control Programme / Ministry of Health

[redacted]

VERSION: FINAL v3.0

DATE: 07 December 2022

This document is the property of Medicines for Malaria Venture and Health Surveillance Secretariat General of Brazil. Medicines for Malaria Venture and Health Surveillance Secretariat General of Brazil are, jointly, the holders of any and all related intellectual property rights, including, but not limited to, copyrights. This document is confidential. It is not to be copied or distributed to other parties without prior written authorisation from Medicines for Malaria Venture and Health Surveillance Secretariat General of Brazil.

[redacted]

Statistical Analysis Plan for the final analysis - Approval

TRuST Study

Operational Feasibility of Appropriate Plasmodium Vivax Radical Cure with Tafenoquine or Primaquine after Quantitative G6PD Testing in Brazil

Protocol:

Number: MMV_TQ_18_02

Version: 6 Amendment 4

Date: 23 September 2021 (including amendment)

Statistical Analysis Plan

Version: 3.0

Date: 07 December 2022

This Statistical Analysis Plan has been reviewed and approved by the individuals listed below.

Responsible parties	Signatures and sign dates (dd-mmm-yyyy)
Stephan Duparc, Chief Medical Office<br><br>Medicines for Malaria Venture	[redacted]
Sally Trufan, Statistical Scientist<br><br>ICON	[redacted]

Table of Contents

STATISTICAL ANALYSIS PLAN FOR THE FINAL ANALYSIS - APPROVAL **2**

TABLE OF CONTENTS **3**

ABBREVIATIONS **5**

1 INTRODUCTION **6**

2 STUDY OBJECTIVES **7**

2.1 Primary Objective **7**

2.2 Secondary Objectives **7**

3 STUDY DESIGN **8**

3.1 Description of the Study Design **8**

3.2 Medical Products **10**

3.3 G6PD Diagnostic Test **10**

3.4 Sample Size Rationale **10**

3.5 Study Population **11**

3.5.1 Health Care Facilities 11

3.5.2 Patients 12

3.6 Endpoints **12**

3.6.1 Primary Endpoints 12

3.6.2 Secondary Endpoints 12

4 STUDY VARIABLES **13**

4.1 Demographics and Baseline Characteristics **13**

4.2 G6PD Enzyme Activity **13**

4.3 Malaria Treatments **13**

4.4 Clinic Characteristics **14**

4.5 Safety Assessments **14**

5 STATISTICAL METHODOLOGY **16**

5.1 General Considerations **16**

5.1.1 Statistical Rules and Data Formats 16

5.1.2 Missing Data 16

5.1.3 Outliers 16

5.1.4 Analysis Time Points 16

5.1.5 Statistical Software 17

5.1.6 Statistical Output 17

5.2 Analysis Sets **17**

5.3 Subgroups Analyses **17**

DocUUID : cad3782b-e7e5-4e92-b6d8-aa95f83460f9

5.4 Interim Analyses 19

5.5 Multicenter Study 19

5.6 Analysis Plan 19

5.6.1 Patient Disposition 19

5.6.2 Demographics and Baseline Characteristics 20

5.6.3 G6PD Test and Hemoglobin Level 21

5.6.4 Multiple *P. vivax* Episodes 21

5.6.5 Malaria Treatments 21

5.6.6 Primary Analysis: Appropriate Use of TQ 22

5.6.7 Secondary Analyses 23

5.6.8 Other Safety Analyses 25

5.6.9 Malaria Follow-up at Day 5 26

6 QUALITY AND VERSION CONTROL 27

7 REFERENCES 27

APPENDIX 1: SCHEDULE OF ASSESSMENTS 28

APPENDIX 2: PLANNED OUTPUT 29

APPENDIX 3: REVISION HISTORY 33

DocUUID : cad3782b-e7e5-4e92-b6d8-aa95f83460f9

Abbreviations

Abbreviation	Definition
AHA	Acute Hemolytic Anemia
AL	Artemether-lumefrantrine
ANVISA	Brazilian health regulatory agency
AS	Artesunate
ASMQ	Artesunate-mefloquine
CI	Confidence Interval
CQ	Chloroquine
FMT	Tropical Medicine Foundation
G6PD	Glucose-6-Phosphate Dehydrogenase
HCF	Health Care Facilities
HCP	Health Care Provider
ICF	Informed Consent Form
Max	Maximum
Min	Minimum
NMCP	National Malaria Control Programme
P. vivax	*Plasmodium vivax*
PCR	Polymerase chain reaction
PDF	Portable Document Format
PQ	Primaquine
RDT	Rapid Diagnostic Test
SAP	Statistical Analysis Plan
SE	Standard Error
SD	Standard Deviation
SIVEP-Malaria	Epidemiological surveillance system for malaria
SOC	System Organ Class
TFL	Tables, Figures, and Listings
TQ	Tafenoquine

DocUUID : cad3782b-e7e5-4e92-b6d8-aa95f83460f9

1 INTRODUCTION

This statistical analysis plan (SAP) is intended to provide a detailed description of the planned analyses for the National Malaria Control Programme/Ministry of Health, Brazil, and Medicines for Malaria Venture (MMV), Protocol MMV_TQ_18_02, version 6, 23 September 2021 entitled "Operational Feasibility of Appropriate *Plasmodium Vivax* Radical Cure with Tafenoquine or Primaquine after Quantitative G6PD Testing in Brazil (TRuST Study)".

The first interim analysis for Phase 1 (described in Section 3.1 below) took place in November 2021, and was performed under SAP version 1.0. The second interim analysis for Phase 2 took place in September 2022 and was performed under SAP version 1.2. The final analysis for this study will be conducted using this SAP, version 3.0.

Currently, primaquine (PQ) is the only therapy widely available for treating hepatic stages of malaria with the high risk of acute hemolytic anemia (AHA) in individuals with glucose-6-phosphate dehydrogenase (G6PD) deficiency (G6PD enzyme activity <30% of normal).

Tafenoquine (TQ) is a slowly eliminated primaquine analogue with a longer half-life (~2 weeks), allowing for a single dose regimen to provide radical cure (in combination with 3-day chloroquine (CQ) treatment) [1, 2]. This simplified dosing regimen may be a significant advance in improving the adherence issues associated with PQ and thereby improving regimen effectiveness in real-life settings.

TQ has been approved for use in Brazil, by the Brazilian Health Regulatory Agency (ANVISA). The municipalities of Manaus (Amazonas State) and Porto Velho (Rondônia State) will officially recommend the use of quantitative G6PD testing before *P. vivax* radical cure (PQ or TQ) and TQ as first-choice treatment for *P. vivax* radical cure in patients with normal G6PD enzyme activity, aged ≥16, after G6PD testing.

The present study is designed to investigate the ability of health care facilities (HCFs) in these municipalities to systematically conduct G6PD testing and provide *P. vivax* patients with appropriate radical cure treatment. Following training on the municipality treatment guidelines, antimalarial treatments and G6PD tests should be prescribed by health care providers (HCPs) in accordance with their clinical judgment and usual standard of care.

This study will provide the National Malaria Control Programme (NMCP) with evidence on the operational feasibility of providing appropriate *P. vivax* radical cure following G6PD testing, at various levels of the health services, under programmatic conditions. This evidence and the insights gained will be crucial for the NMCP to decide on revisions to national malaria treatment policies in Brazil.

DocUUID : cad3782b-e7e5-4e92-b6d8-aa95f83460f9

2 STUDY OBJECTIVES

2.1 Primary Objective

The primary objective is to investigate whether *P. vivax* patients ≥16 years are treated with TQ in accordance with the appropriate level of G6PD enzyme activity.

2.2 Secondary Objectives

The secondary objectives are:

- To investigate whether *P. vivax* patients ≥6 months are treated with PQ in accordance with the appropriate level of G6PD enzyme activity.
- To describe the characteristics of patients treated with TQ or PQ.
- To measure frequency of drug-induced signs of AHA and hospitalization due to drug-induced AHA.

3 STUDY DESIGN

Key features of the study design are summarized in the sections below; a full description of the study design can be found in the study protocol.

3.1 Description of the Study Design

This is a retrospective, non-interventional, observational study conducted in Brazil in the municipalities of Manaus (Amazonas State) and Porto Velho (Rondônia State) on patients with *P. vivax* malaria.

Both municipalities will officially define TQ to be the first choice for radical cure of *P. vivax* in patients with normal G6PD enzyme activity, aged ≥16 years with *P. vivax*-confirmed malaria, after G6PD testing. As part of treatment policy in the municipalities of Manaus and Porto Velho, patients will be asked to return for a follow-up visit at Day 5 to confirm blood-stage parasite clearance and to detect any signs of AHA.

Designated staff at health care facilities will be trained on the quantitative G6PD test procedure and the radical cure treatment algorithm.

On a regular basis within the study period, routine retrospective data from the SIVEP form (including collection of G6PD testing, TQ treatment, and signs of hemolysis) will be entered into a new study database by the municipality staff for all patients.

As some patients may not return for the follow-up visit at Day 5, principal investigator staff will request the two referral hospitals likely to see all hospitalizations for AHA to regularly screen hospital electronic admission records for patients presenting signs of AHA (renal failure, jaundice, blood transfusion, malaria). Information about confirmed AHA will be linked with patient data recorded in the database by the Tropical Medicine Foundation (FMT) staff.

The study will be conducted in two phases as described in Figure 3-1:

- During the 1st phase, data will be collected for *P. vivax* patients managed in higher-level health facilities (see definition Section 3.5.1). Once data from 600 *P. vivax* patients ≥16 years who have not been treated for *P. vivax* malaria in the last 60 days are collected in the study, an interim analysis will be conducted and the results will be analyzed (as well as other factors) by an Independent Study Oversight Committee in order to recommend whether the study could be extended to lower-level health facilities.

  If the interim results of the 1st phase are deemed unsatisfactory, the Independent Study Oversight Committee (ISOC) may decide not to recommend an extension of the study to lower-level health facilities until improvements are made in the education program and/or additional HCP support is implemented. Additional interim analyses may be conducted as appropriate.

- During the 2nd phase after ISOC approval, and agreement by the sponsors, the study will be extended to lower-level health facilities (see definition Section 3.5.1) and to additional higher-level health facilities.

  During this 2nd phase, data will continue to be collected for *P. vivax* patients managed by the higher-level health facilities of the 1st phase.

  An additional interim analysis will be conducted after data from 600 *P. vivax* patients ≥16 years who have not been treated for *P. vivax* malaria in the last 60 days from lower-level facilities are collected in the study database (approximately 3 months after

DocUUID : cad3782b-e7e5-4e92-b6d8-aa95f83460f9

the beginning of the 2nd phase) to decide whether the study should continue providing services at such facilities or improvements for health service delivery are needed.

The study will continue while interim analyses are being conducted.

Only de-identified data will be used for statistical analyses. Final results will be reviewed by the ISOC.

It is anticipated that it will take approximately 12-months to carry out the study.

Figure 3-1: TRuST Study Flow

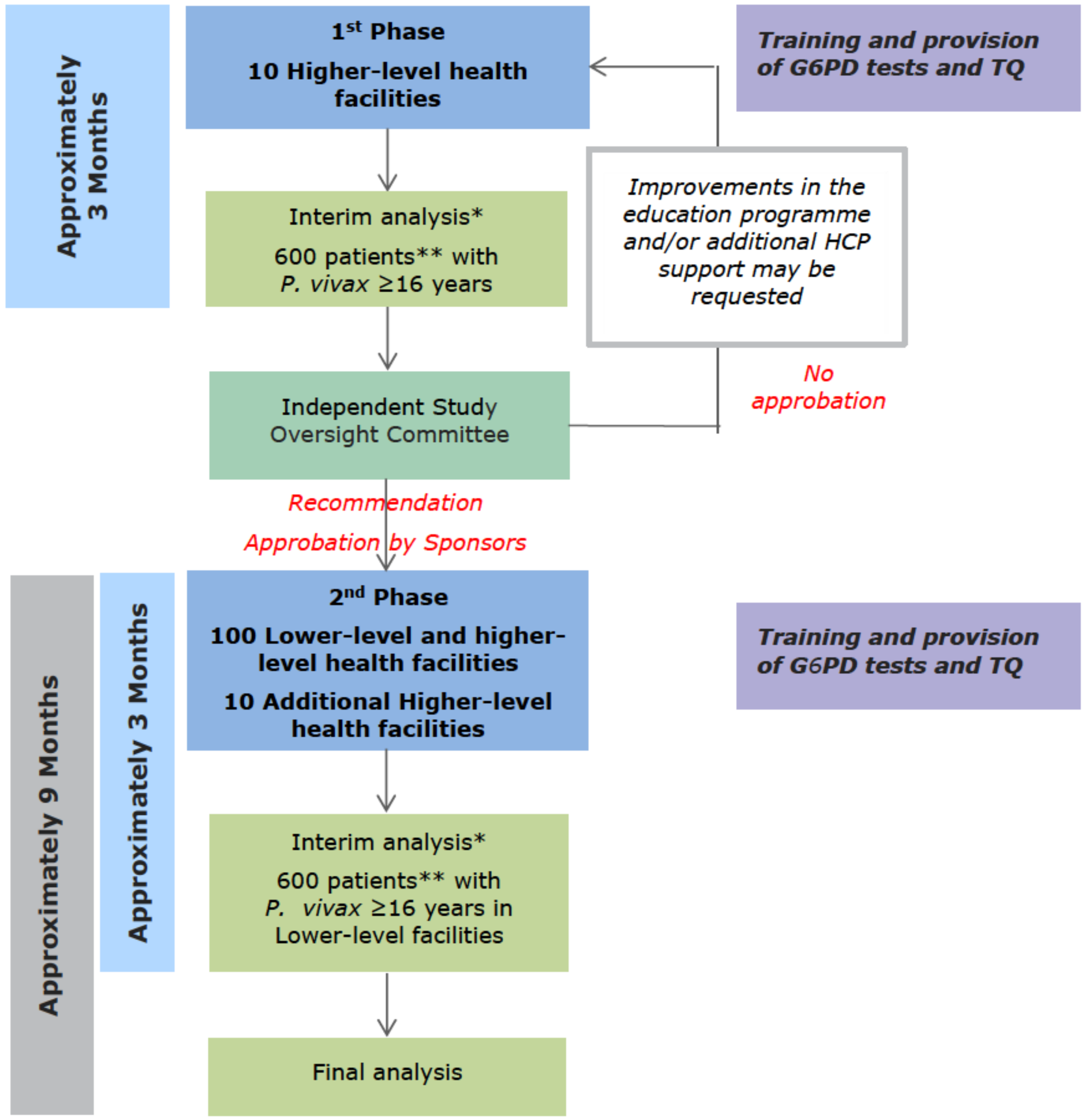

*While interim analyses are being conducted, the study will continue, and health care facilities will continue to access G6PD testing and TQ

**Excluding patients who were treated for *P. vivax* in the last 60 days

3.2 Medical Products

Tafenoquine 300 mg single-dose is an 8-aminoquinoline anti-malarial drug indicated for the radical cure (prevention of relapse) of *P. vivax* malaria. TQ is supplied as 150 mg tablets.

Primaquine is an 8-aminoquinoline anti-malarial drug indicated for the radical cure (prevention of relapse) of *P. vivax* malaria. PQ is supplied as 7.5 mg and 15 mg tablets.

3.3 G6PD Diagnostic Test

The SD Biosensor STANDARD™ G6PD test is a point-of-care, quantitative glucose-6-phosphate dehydrogenase (G6PD) enzyme activity analyzer. It is a colorimetric test and provides the G6PD activity as a ratio to hemoglobin (U/g Hb).

3.4 Sample Size Rationale

The study size calculation was based on the primary endpoint of the study (percentage of patients with *P. vivax* aged ≥16 years with ≥70% G6PD activity treated with TQ and with <70% G6PD activity not treated with TQ among all patients treated with TQ and patients with <70% G6PD activity not treated with TQ). The study size was determined to ensure a satisfactory precision of this percentage.

The precision (e) of the 95% CI of a percentage (i.e., half of the width of the CI) is determined using the following formula:

$$e = 1.96 \times \sqrt{\frac{p(1-p)}{n}}$$

where *p* is the percentage and *n* the study size.

The following table presents the precision and the corresponding 2-sided 95% CI for a percentage of patients with *P. vivax* aged ≥16 years with ≥70% G6PD activity treated with TQ or with <70% G6PD activity not treated with TQ varying from 80% to 99% and a study size of 400 to 5000 patients with *P. vivax* treated with TQ or with <70% G6PD activity not treated with TQ.

Table 3-1 Precision and 95% CI obtained for percentages from 80% to 99% with study sizes of 400, 500, 1000, 2000 and 5000

	Percentage									
	80%		90%		95%		97.5%		99%	
N	e (%)	95% CI	e (%)	95% CI	e (%)	95% CI	e (%)	95% CI	e (%)	95% CI
400	3.9	76.1;83.9	2.9	87.1;92.9	2.1	92.9;97.1	1.5	96.0;99.0	1.0	98.0;100.0
500	3.5	76.5;83.5	2.6	87.4;92.6	1.9	93.1;96.9	1.4	96.1;98.9	0.9	98.1;99.9
1000	2.5	77.5;82.5	1.9	88.1;91.9	1.4	93.6;96.4	1.0	96.5;98.5	0.6	98.4;99.6
2000	1.8	78.2;81.8	1.3	88.7;91.3	1.0	94.0;96.0	0.7	96.8;98.2	0.4	98.6;99.4
5000	1.1	78.9;81.1	0.8	89.2;90.8	0.6	94.4;95.6	0.4	97.1;97.9	0.3	98.7;99.3
8000	0.7	79.3,80.7	0.6	89.4,90.6	0.4	94.6,95.4	0.3	97.2,97.8	0.2	98.8, 99.2

e: precision of the 95% CI.

For example, with 500 patients, the 95% CI for a percentage of 97.5% is (96.1%, 98.9%), and the precision 1.4%. With 400 patients, the 95% CI for a percentage of 97.5% is (96.0%, 99.0%), and the precision 1.5%.

Interim analyses

For interim analyses, 500 patients were considered appropriate to estimate the primary endpoint with an acceptable precision. Assuming that *P. vivax* patients treated with TQ and patients with <70% G6PD activity not treated with TQ will represent approximately 85% of *P. vivax* patients ≥16 years, interim analyses will be conducted after data from 600 *P. vivax* patients ≥16 years who have not been treated for *P. vivax* malaria in the last 60 days are collected in the study database to enable an assessment of the primary endpoint based on 500 patients.

Overall study size

In 2017, 16,294 and 3,517 *P. vivax* malaria cases were reported in the municipalities of Manaus and Porto Velho, respectively. Overall, 16,000 *P. vivax* patients are expected to be included in the study (approximately 10,000 in higher-level health facilities and 6,000 in lower-level health facilities). Assuming that *P. vivax* patients ≥16 years will represent approximately 60% of *P. vivax* patients and that *P. vivax* patients treated with TQ and *P. vivax* patients with <70% G6PD activity not treated with TQ will represent approximately 85% of *P. vivax* patients ≥16 years who have not been treated for *P. vivax* malaria in the last 60 days, the primary endpoint was anticipated to be assessed in approximately 8,000 patients. However, due to external circumstances including the COVID-19 pandemic and climate changes that affected malaria transmission during the study time, approximately 6,000 patients will be assessed in the final analysis of the study and the primary endpoint will be assessed in at least 4,000 patients. This will still provide adequate precision for this analysis as shown in Table 3-2. Only de-identified data will be used for statistical analyses.

Table 3-2: Precision and 95%CI obtained for percentages from 80% to 99% with study size of 4000 patients

	**Percentage**							
	**80%**		**90%**		**95%**		**99%**	
**N**	**e (%)**	**95% CI**	**e (%)**	**95% CI**	**e (%)**	**95% CI**	**e (%)**	**95% CI**
**4000**	1.2	78.8;81.2	0.9	89.1;90.9	0.7	94.3;95.7	0.3	98.7;99.3

3.5 Study Population

3.5.1 Health Care Facilities

Health care facilities are divided in higher/ medium-level facilities (1-Hospitals, 2-Emergency care/Other) and lower-level facilities (3-Basic health units (UBS), 4-Operational bases 5-Laboratories). For reporting purposes, laboratories will be grouped as Operational bases.

During the 1st phase, only higher/medium-level health facilities will be trained and will collect information; if recommended by the ISOC and approved by the sponsors, the 2nd phase will be initiated including lower-level health facilities and additional higher-level health facilities.

DocUUID : cad3782b-e7e5-4e92-b6d8-aa95f83460f9

3.5.2 Patients

Patient selection is based on the inclusion and exclusion criteria listed below. Patients who meet each of the inclusion criteria and none of the exclusion criteria are eligible to participate in this study.

Inclusion criteria:

A patient must meet the following criterion to be eligible for the study:

- Diagnosed with mono-species *P. vivax* malaria (parasitologically confirmed through microscopy or rapid diagnostic test)
- Diagnosed with *P. vivax*/*P. falciparum* malaria mixed infection (parasitologically confirmed through microscopy or rapid diagnostic test) and older than 6 months of age
- Diagnosed negative for *P. vivax* mono-species malaria or *P. vivax*/ *P. falciparum* malaria mixed infection but had a positive diagnosis in the past

Exclusion criteria:

None.

3.6 Endpoints

3.6.1 Primary Endpoints

The primary endpoint will be the percentage of *P. vivax* patients aged ≥ 16 years, excluding those who were treated for *P. vivax* malaria in the last 60 days, treated or not with TQ in accordance with the appropriate level of G6PD enzyme activity.

3.6.2 Secondary Endpoints

Secondary endpoints will be the following:

1. Percentage of *P. vivax* patients aged ≥6 months treated or not with daily PQ in accordance with the appropriate level of G6PD enzyme activity.
2. Description of the characteristics of patients (age, gender, ethnicity, weight, malaria history, malaria diagnosis, hemoglobin, pregnancies, G6PD activity) treated with TQ or with PQ.
3. Frequency of confirmed drug-induced AHA and hospitalization due to drug-induced AHA.

DocUUID : cad3782b-e7e5-4e92-b6d8-aa95f83460f9

4 STUDY VARIABLES

The frequency and timing of the observations or assessment for this study can be found in the schedule of assessments in Appendix 1.

Information is collected from SIVEP form unless otherwise specified. The variables analyzed as part of the study are described in the following sections.

4.1 Demographics and Baseline Characteristics

Demographics collected from SIVEP form include age (years), sex (Female/Male), ethnicity (White, Black, Yellow, Mixed, Indigenous), education (illiterate, 1st to 5th year, 5th year, 6th to 9th year, completed basic education, incomplete high school, complete high school, incomplete college, complete college, not applicable) and occupation in last 15 days (agriculture, livestock, household, tourism, gold digging, farming, hunting/fishing, construction, mining, traveller, others).

Other following baseline characteristics collected from SIVEP form include:

- Patient consent for secondary use of their data (yes)
- Weight in kilograms (continuous)
- Pregnancy status (1st trimester, 2nd trimester, 3rd trimester, Unsure, No, Not applicable)
- Breastfeeding (yes/no) and breastfeeding duration (days)
- Malaria history: treated for *P. vivax* in the last 60 days (Yes/No), treated for *P. falciparum* in the last 40 days (Yes/No)
- Malaria diagnosis:
  - Date of first symptoms (dd/mm/yyyy)
  - Diagnostic test (date (dd/mm/yyyy); type of test: (blood slide, RDT, PCR)
  - Parasitemia: parasites per mm^3, parasitemia in crosses (<half cross, half cross, one cross, 2 crosses, 3 crosses, 4 crosses)

4.2 G6PD Enzyme Activity

- Hemoglobin level (g/dL)
- G6PD enzyme activity (U/g Hb)
  - Deficient (G6PD ≤4.0 U/g Hb)
  - Intermediate (4.1 to 6.0 U/g Hb)
  - Normal (≥6.1 U/g Hb)
- Source of information
  - Test
  - Patient card

4.3 Malaria Treatments

The malaria treatment information collected from SIVEP form includes:

DocUUID : cad3782b-e7e5-4e92-b6d8-aa95f83460f9

- Anti-malarial drugs administered and treatment duration
  - Blood stage treatment: CQ 3 days, AL, ASMQ, Injectable AS, Weekly CQ, CQ 3-day + weekly CQ, Other with specification, No treatment reported.
  - Liver stage /gametocyte: None, PQ 7-days, PQ 14-days, PQ single dose, PQ weekly, TQ, Other with specification (dd/mm/yyyy). For reporting purposes, PQ single dose is categorized as "none" as the dose is inappropriate for *P. vivax* radical cure.
- Start date

4.4 Clinic Characteristics

The clinic characteristics collected from SIVEP form include:

- Municipality (name and code)
- Health facility (name and code)
- HCP (code)

4.5 Safety Assessments

Safety assessments at Day 5 (follow-up visit) include signs of hemolysis following treatment initiation (jaundice, dark urine, other) collected from the SIVEP Form.

SIVEP records are linked with hospital records at the referral hospitals and with the state hospital database to identify hospitalization after malaria episodes as a starting point to identify potential cases of AHA. Each positive crosslink will be further investigated for signs of AHA. A report with true pairs generated from the probabilistic linkage will be issued to the head of the local malaria surveillance department for investigation.

In addition, SIVEP records are linked with the mortality information system to identify deaths among positive and negative notifications and understand causality.

NB: Only patients who have consented to data sharing are linked and investigated.

In case of confirmed AHAs (irrespective of the relationship with primaquine or tafenoquine) detected through this process at the two referral hospitals and in other hospitals in the municipalities, the following additional data will be collected from patient medical records from the hospitals, and will be linked with the data collected in the SIVEP form:

- Time to onset since start *P. vivax* treatment (TQ/PQ)
- Hemoglobin level
- Laboratory results
- AHA signs and symptoms
- AHA treatment (blood transfusion, dialysis, other)
- Hospitalization (hospitalization date, discharge date)
- Hospitalization outcomes (ongoing, complete recovery, improved with disability, death)
- Diagnosis (primary and secondary)

DocUUID : cad3782b-e7e5-4e92-b6d8-aa95f83460f9

In case of death;

- Treatment given (TQ/PQ)
- Time since start of *P. vivax* treatment (TQ/PQ)
- G6PD status.

Information about AHA cases and deaths are provided in detailed reports.

Although the protocol included the linkage of HCP training records with cases of AHA, there is no value in doing so as the SIVEP form is not necessarily filled out by the person who performs the G6PD test / selects the treatment.

DocUUID : cad3782b-e7e5-4e92-b6d8-aa95f83460f9

5 STATISTICAL METHODOLOGY

5.1 General Considerations

5.1.1 Statistical Rules and Data Formats

Quantitative (continuous) variables will be described by the number of observations, mean, standard deviation (SD), median, first and third quartiles (Q1-Q3), extreme values (minimum and maximum).

Qualitative (categorical) variables will be described by the absolute (*n*) and relative (%) frequency of each response category. For categorical variables with at least one missing observation, a category of 'missing' will be added to document the number of missing observations. The percentages of missing observations will be applicable for the categorical variables, summarized by relative (%) frequency, based on the total number of patients (i.e., assessed and missing). If there is no missing observation, a category of 'missing' may not be added. In tables describing G6PD activity distributions, the number missing will not be included in the denominator for the percentage calculation.

Means, medians, and quartiles will be rounded to one decimal more than that with which the original data are recorded. The standard deviation will be rounded to two decimals more than the original data. Minimum and maximum values will be reported with the same number of decimals with which the original data are recorded.

Percentages will be rounded to one decimal place.

Confidence intervals for means and percentages will be derived when appropriate using Clopper-Pearson method (for percentage) and will be two-sided at the 95% level unless otherwise specified.

Figures will also be provided when appropriate.

Analysis will be purely descriptive.

5.1.2 Missing Data

No imputation of missing data will be done.

5.1.3 Outliers

No adjustment for outliers will be done.

5.1.4 Analysis Time Points

For analysis, the baseline will be defined as the date of treatment initiation specified in the SIVEP form. The number of days from the baseline will be used to define other analysis time points.

A follow-up visit is planned at Day 5 starting from baseline.

Another follow-up can be completed from the referral hospital and the date of admission to the hospital will be used to compute the number of days from the baseline.

DocUUID : cad3782b-e7e5-4e92-b6d8-aa95f83460f9

5.1.5 Statistical Software

The analysis will be performed under SAS® software version 9.4 or later.

5.1.6 Statistical Output

An analysis dataset will be created from the raw electronic data capture (EDC) data set, delivered in CSV format, to facilitate programming of Tables, Figures, and Listings (TFL).

Proposed shells for TFL will be provided as listed in the planned output (See Appendix 2).

TFL output will be provided in PDF format.

5.2 Analysis Sets

Analysis population: Populations will be defined by malaria species: mono-vivax infections (new cases or new infection within 60 days) and mixed *P. falciparum / P. vivax* infections). All patients who have met the selection criteria will be considered in the Analysis Population. NB: On review of the data from the second interim analysis, and before the database lock for the final analysis, it was decided to run separate analysis on patients with new infections within 60 days as they are not included in the primary endpoint. Analysis of mixed infection cases was planned in SAP V1.2 for the analysis of the phase 2 of the study, however, was not done.

Safety population: All patients having received at least 1 dose of TQ or more than 1 dose of PQ will be considered in the Safety Population.

5.3 Subgroups Analyses

After finalization of the protocol a subgroup of type of infection was deemed to be of interest. This will investigate differences between vivax mono infection and mixed infection.

Vivax mono infection:

Primary population: New cases

Select analyses will be performed for all new vivax cases by anti-malarial treatment (Patients treated with TQ, Patients treated with PQ, Patients treated with 7-day PQ, Patients treated with 14-day PQ, Patients treated with weekly PQ, Patients not treated with TQ/PQ).

In addition, the following subgroups will be considered for analysis as applicable:

- Facility level and type: patients from higher/medium-level facilities (1-Hospitals, 2-Emergency care / Other) and patients from lower-level facilities (3-Basic health units (UBS), 4-Operational bases). For reporting purposes laboratories will be grouped as Operational bases
- Age classes (≥6 months to <5 years; ≥5 to <16 years; ≥16 to <30 years; ≥30 to <50 years; ≥50 to <70 years; ≥70 years)
- Sex (Male; Female)
- Ethnic groups (White; Black; Yellow; Mixed; Indigenous)
- G6PD activity (Deficient: ≤4.0 U/g Hb; Intermediate: 4.1-6.0 U/g Hb; Normal: ≥6.1 U/g Hb)

- Weight (kilograms) will be operationalized in two ways:
  - ≥5 to <10, ≥10 to<25, ≥25 to <35, ≥35 to <50, ≥50 to <60, ≥60 to <70, ≥70 to <80, ≥80 to <90, ≥90 to <120, and ≥120
  - ≥5 to <50, ≥50 to <70, ≥70 to <90, ≥90 to <120, and ≥120

Additional subgroups of interest (to be defined prospectively) may also be examined, as deemed appropriate.

In addition, some analyses performed by anti-malarial treatment and/or facility level/type, will also be performed in patients from the municipality of Manaus and in patients from Porto Velho.

Patients with positive mono vivax infection within 60 days

As mentioned under 5.2. this subgroup has been added for the final SAP / analysis.

Patients returning with a positive mono vivax infection within 60 days of the initial infection will be analysed separately by anti-malarial treatment (Patients treated with TQ, Patients treated with PQ, Patients treated with 7-day PQ, Patients treated with 14-day PQ, Patients treated with weekly PQ, Patients not treated with TQ/PQ).

In addition, the following subgroups will be considered for analysis as applicable:

- Anti-malarial treatment for first infection (TQ, 7-day PQ, 14-day PQ, weekly PQ, Not treated with TQ/PQ)
- Facility level and type: patients from higher/medium-level facilities (1-Hospitals, 2-Emergency care / other) and patients from lower-level facilities (3-Basic health units (UBS), 4-Operational base). For reporting purposes laboratories will be grouped as Operational bases.
- Age classes (≥6 months to ≤5 years; >5 to <16 years; ≥16 to ≤30 years; >30 to ≤50 years; >50 to ≤70 years; >70 years)
- Sex (Male; Female)
- Ethnic groups (White, Black, Yellow, Mixed, and Indigenous)
- G6PD activity (deficient: ≤4.0 U/g Hb, intermediate: 4.1-6.0 U/g Hb, normal: ≥6.1 U/g Hb)
- Weight (kilograms) ≥5 to <50, ≥50 to <70, ≥70 to <90, ≥90 to <120, and ≥120

Mixed infections

All analyses will be performed for all mixed *Pf/Pv* infection cases by anti-malarial treatment (Patients treated with TQ, Patients treated with PQ, Patients treated with 7-day PQ, Patients treated with 14-day PQ, Patients treated with weekly PQ, Patients not treated with TQ/PQ).

In addition, the following subgroups will be considered for analysis as applicable:

- Facility level and type: patients from higher/medium-level facilities (1-Hospitals, 2-Emergency care / Other) and patients from lower-level facilities (3-Basic health units (UBS), 4-Operational bases). For reporting purposes laboratories will be grouped as Operational bases.

DocUUID : cad3782b-e7e5-4e92-b6d8-aa95f83460f9

- Age classes (≥6 months to <5 years; ≥5 to <16 years; ≥16 to <30 years; ≥30 to <50 years; ≥50 to <70 years; ≥70 years)
- Sex (Male; Female)
- Ethnic groups (White, Black, Yellow, Mixed, and Indigenous)
- G6PD activity (deficient: ≤4.0 U/g Hb, intermediate: 4.1-6.0 U/g Hb, normal: ≥6.1 U/g Hb)
- Weight (kilograms) ≥5 to <50, ≥50 to <70, ≥70 to <90, ≥90 to <120, and ≥120

5.4 Interim Analyses

A first interim analysis will be conducted after data from 600 *P. vivax* patients ≥16 years who have not been treated for *P. vivax* malaria in the last 60 days are collected in higher-level health facilities (all patient data collected from higher-level health facilities at the data cut-off will be analyzed).

An additional interim analysis will be conducted after data from 600 *P. vivax* patients ≥16 years who have not been treated for *P. vivax* malaria in the last 60 days are collected in lower-level facilities (all patient data collected from higher- and lower-level health facilities at the data cut-off will be analyzed).

5.5 Multicenter Study

During the 1st phase, the study was finally conducted in 9 higher-level health facilities and not 10 as initially planned. During the 2nd phase, the study was conducted in approximately 40 lower-level health facilities and not 100 as initially planned. No new higher-level health facility were involved while it was planned to conduct the study in 10 additional sites. The number of health facilities was lower than originally envisaged, due to the impact of Covid-19 on the organization of health services.

Taking into account the two phases, the study has been conducted in approximately 50 sites in Brazil instead of 120 as initially planned.

5.6 Analysis Plan

All analyses will be performed overall and by anti-malarial treatment. Other subgroups analysis as defined in Section 5.3 will be performed for primary and secondary endpoints.

The percentages will be calculated based on the respective analysis set or the number of non-missing observations (i.e., the number of patients assessed) in the respective analysis unless otherwise specified in the sections below.

Descriptive continuous and categorical variables will be summarized as defined in Section 5.1.1.

5.6.1 Patient Disposition

Patient disposition will be summarized overall for each subgroup and by anti-malarial treatment for each municipality using descriptive statistics (see Section 5.1.1) for the following parameters: number of patients included in the study database, number and percentage of patients included in the Analysis Population and reason for exclusion and

DocUUID : cad3782b-e7e5-4e92-b6d8-aa95f83460f9

number and percentage of patients included in the Safety Population and reason of exclusion, number and percentage of patients included from higher/medium- and lower-level of facilities and number and percentage of patients returning for Day 5 follow-up.

5.6.2 Demographics and Baseline Characteristics

Socio-demographics and baseline characteristics will be summarized using descriptive statistics (see Section 5.1.1) for the overall analysis population of each sub-group and by anti-malarial treatment, by anti-malarial treatment for each municipality, by facility level and type, and by sex (definition of subgroups in Section 5.3).

- Socio-demographic characteristics, in line with the SIVEP form, will be summarized for the following parameters: age (years), age in class (6 months to ≤5 years; >5 to <16 years; ≥16 to <30 years; ≥30 to <50 years; ≥50 to <70 years; ≥70 years), sex (Male, Female), ethnic group (White, Black, Yellow, Mixed, Indigenous), education ((illiterate, 1st to 5th year, 5th year, 6th to 9th year, Complete basic education, Incomplete high school, Complete high school, Incomplete college, Complete college, Not applicable), weight (kilograms) as a continuous variable and in class (≥5 to <10, ≥10 to<25, ≥25 to <35, ≥35 to <50, ≥50 to <60, ≥60 to <70, ≥70 to <80, ≥80 to <90, ≥90 to <120, and ≥120; ≥5 to <50, ≥50 to <70, ≥70 to <90, ≥90 to <120, and ≥120) and for female patients of childbearing potential (16 to <50 years), pregnancy (Yes/No/missing), pregnancy status (1st trimester, 2nd trimester, 3rd trimester, Unsure, No, Not applicable), breastfeeding (Yes/No/missing) and breastfeeding duration.

The following baseline characteristics related to the disease will also be summarized:

- Malaria history: treated for *P. vivax* in the last 60 days (Yes/No); treated for *P. falciparum* in the last 40 days (Yes/No); Blood transfusion in the last 60 days (Yes/No)
- Malaria diagnosis: time since first symptoms (days), time since diagnostic test (days), type of test (blood slide, RDT, PCR), parasites per mm^3, parasitemia in class (<half cross, half cross, one cross, 2 crosses, 3 crosses, 4 crosses)

DocUUID : cad3782b-e7e5-4e92-b6d8-aa95f83460f9

5.6.3 G6PD Test and Hemoglobin Level

The following analyses will be produced using descriptive statistics (see Section 5.1.1) for the overall analysis population and by the subgroups defined in Section 5.3: anti-malarial treatment for each municipality, facility level and type for each municipality, sex. and ethnic groups.

The number and percentage of *P. vivax* patients tested for G6PD deficiency will be summarized as well as the distribution of G6PD test result, source of information (G6PD test; G6PD card), G6PD activity in class (≤4.0 U/g Hb; 4.1-6.0 U/g Hb; ≥6.1 U/g Hb) and in percentage in overall patients (excluding those with missing G6PD activity), patients <16 years and patients ≥16 years, pregnant women and hemoglobin levels (g/dL) (mean, median, Q1:Q3; min; max; <7 g/dL (n and %).

Among the *P. vivax* patients treated with TQ, the number and percentage of patients with ≥70% G6PD activity, with <70% G6PD activity and with no G6PD test will be described in overall patients, patients <16 years and patients ≥16 years.

Among the *P. vivax* patients treated with PQ, the number and percentage of patients with ≥30% G6PD activity, with <30% G6PD activity and with no G6PD test will be described.

G6PD activity by sex (male/female) and by G6PD category (deficient, intermediate, normal) will be summarized graphically (vertical bar charts and box charts; excluding those with missing G6PD activity and including those females who are pregnant). Mean hemoglobin levels by sex (male/female) will be summarized graphically (box charts, excluding those with missing Hb).

Addition to the final SAP / TFLs: Listing of G6PD activity in patients with Hb <7 g/dL and antimalarial treatment will be produced.

The source of information for G6PD activity will also be analyzed.

5.6.4 Multiple *P. vivax* Episodes

The number and percentage of patients returning with new episode of *P. vivax* before or at Day 29 (recrudescence), and after Day 29 (recurrence/relapse), the number and the distribution of recurrences will be summarized using descriptive statistics as described Section 5.1.1 for the overall analysis population and in the subgroups of interest: anti-malaria treatment (and for each municipality), sex, and weight categories (defined in Section 5.3).

The number of symptomatic *P. vivax* recurrences will also be presented graphically (histogram) by anti-malarial treatment.

The variability in G6PD status and hemoglobin level in same patient over multiple *P. vivax* episodes (assumes same unique patient identifier in database) will be summarized:

- % change for each G6PD category status (Normal, Intermediate, Deficient) in same female patients and in male patients who were retested
- % change will also be summarized as a shift table between categories at baseline and categories at the time of recurrence

5.6.5 Malaria Treatments

Anti-malarial drugs administered for blood stage and liver stage as listed in Section 4.3 will be presented using descriptive summary statistics for the overall analysis population

and by anti-malarial treatment for each municipality, and by facility level and type for each municipality (see definition in Section 5.3): the number and percentage of patients with each treatment administered and intended treatment will be also summarized in patients from Manaus and Porto Velho.

5.6.6 Primary Analysis: Appropriate Use of TQ

The following analyses will be performed for the analysis population and in the subgroups of interest defined in Section 5.3: facility level and type for each municipality and overall, and sex (except for analysis with facility as denominator).

The number and percentage of patients aged ≥16 years who were not treated for *P. vivax* in the last 60 days will be summarized using descriptive statistics by treated or not with TQ in accordance with the appropriate level of G6PD enzyme activity.

The primary endpoint will be calculated as follows:

Among patients aged ≥16 years excluding patients who were treated for *P. vivax* malaria in the last 60 days:

$$\frac{\text{Appropriate use of TQ* + Appropriate non-use of TQ**}}{\text{Total use of TQ*** + Appropriate non-use of TQ**}}$$

* Number of patients with ≥70% G6PD activity treated with TQ
** Number of patients with <70% G6PD activity + patients with missing G6PD activity not treated with TQ
*** Number of patients treated with TQ

In addition, the percentage of *P. vivax* patients receiving TQ based on correct application of treatment algorithm (G6PD activity, age, non-breastfeeding, and non-pregnant) will be presented using descriptive summary statistics.

Definitions based on the treatment algorithm:

Appropriate use of TQ is defined as G6PD activity ≥70% (≥6.1 U/g Hb), age ≥16 years, non-breastfeeding (or missing breastfeeding status female <16 years old or ≥50 years old), and non-pregnant (or not applicable) treated with TQ.

Inappropriate use of TQ is defined as G6PD activity <70% (≤6.0 U/g Hb) or missing G6PD activity, or age <16 years, breastfeeding (or missing breastfeeding status female ≥16 years - <50 years old), or pregnant (or missing pregnant status in females ≥16 years - <50 years old) treated with TQ.

Inappropriate non-use of TQ is defined as G6PD activity ≥70% (≥6.1 U/g Hb), age ≥16 years, non-breastfeeding (or missing breastfeeding status female ≥50 years old), and non-pregnant (or not applicable) and NOT treated with TQ.

Appropriate non-use of TQ is defined as G6PD activity <70% (≤6.0 U/g Hb) or missing G6PD activity or age <16 years, or breastfeeding (or missing breastfeeding status female ≥16 years - <50 years old), or pregnant (or missing pregnant status in females ≥16 years - <50 years old) and NOT treated with TQ.

A listing of patients receiving inappropriate TQ treatment, grouped by category of inappropriate use, will be produced.

The percentage of *P. vivax* patients receiving TQ based on correct G6PD activity, age, non-breastfeeding, non-pregnant, and receiving CQ blood stage treatment will be calculated separately for the patients of each facility level and type.

NB: Denominators for calculating percentages will vary as follows:

DocUUID : cad3782b-e7e5-4e92-b6d8-aa95f83460f9

- G6PD activity: Will include missing G6PD activity
- Women of childbearing age defined in this study as ≥16 years - <50 years old will serve as the denominator for breastfeeding and pregnant women

The percentage of facilities providing TQ based on correct G6PD activity, age, non-breastfeeding, non-pregnant, and CQ blood stage treatment will be calculated for each facility level and type using the following categories: <50%, 50% to <75%, 75% to <90%, 90% - <100% and 100% of patients.

5.6.7 Secondary Analyses

5.6.7.1 Secondary Endpoint 1: Appropriate Use of PQ

The following analyses will be performed for the analysis population and in the subgroups of interest defined in Section 5.3: facility level and type for each municipality and overall, and sex (except for analysis at facility level).

- The number and percentage of *P. vivax* patients aged ≥6 months treated or not with daily PQ in accordance with the appropriate level of G6PD enzyme activity (≥30%) will be summarized using descriptive statistics for the analysis population and by facility level and type, and by sex (subgroups defined in Section 5.3). Daily PQ is defined as receiving either the 7-day or 14-day treatment regimen of PQ.
-

Among patients aged ≥6 months:

$$\frac{\text{Appropriate use of daily PQ* + Appropriate non-use of daily PQ**}}{\text{Total use of PQ*** + Appropriate non-use of daily PQ**}}$$

* Number of patients with ≥30% G6PD activity treated with daily PQ
** Number of patients with <30% G6PD activity + with missing G6PD activity not treated with daily PQ
*** Number of patients treated with daily PQ

In addition, the percentage of *P. vivax* patients receiving daily PQ based on correct application of treatment algorithm (G6PD activity, age, breastfeeding from the second month of lactation, non-pregnant).

The correct application of treatment algorithm is defined as:

1. Correct application of daily PQ: Defined by G6PD activity ≥30% (≥4.1 U/g Hb), age ≥6 months, non-breastfeeding/breastfeeding from ≥30 days of lactation (or missing breastfeeding status female <16 years old or ≥50 years old), and non-pregnant (or not applicable).
2. Patients receiving 7-day PQ: Defined by G6PD activity ≥30% (≥4.1 U/g Hb), age ≥6 months, non-breastfeeding/breastfeeding from ≥30 days of lactation (or missing breastfeeding status female <16 years old or ≥50 years old), and non-pregnant (or not applicable) **and not treated for *P. vivax* malaria in the last 60 days.**
3. Patients receiving 14-day PQ: Defined by G6PD activity ≥30% (≥4.1 U/g Hb), age ≥6 months, non-breastfeeding/breastfeeding from ≥30 days of lactation (or missing breastfeeding status female <16 years old or ≥50 years old), and non-pregnant (or not applicable) **and treated for *P. vivax* malaria in the last 60 days.**

4. Patients receiving weekly PQ: Defined by **G6PD activity <30% (**≤4.0 U/g Hb) or missing G6PD activity, age ≥6 months, non-breastfeeding/breastfeeding from ≥30 days of lactation (or missing breastfeeding status female <16 years old or ≥50 years old), and non-pregnant (or not applicable).
5. Correct application of PQ (daily and weekly): Defined by 1 and 4 above.

A listing of patients receiving inappropriate PQ treatment will be produced grouped by category of inappropriate use.

The percentage of *P. vivax* patients receiving daily PQ based on correct application of treatment algorithm (G6PD activity, age, breastfeeding from ≥30 days of lactation, non-pregnant) will be calculated for each facility level and type.

The percentage of facilities providing daily PQ based on correct G6PD activity, age, non-breastfeeding for <30 days, non-pregnant will be calculated for each facility level and type using the following categories: <50%, 50% to <75%, 75% to <90%, 90% - <100% and 100% of patients.

5.6.7.2 Secondary Endpoint 2: Patient Characteristics

Patient baseline characteristics and G6PD activity of patients treated with TQ and of patients treated with PQ will be presented using descriptive statistics for the analysis population and in the subgroups defined in Section 5.3.

Refer to analysis description provided in the Sections 5.6.2 Demographics and Baseline Characteristics and 5.6.3 G6PD Test.

5.6.7.3 Secondary Endpoint 3: Confirmed AHA (whatever is the relationship with the investigational drugs)

Suspected AHA will be presented using descriptive summary statistics as described in Section 5.1.1 for the overall safety population and in the subgroups: anti-malarial treatment (for each municipality and overall), facility level and type (for each municipality and overall), age classes, sex, and G6PD activity (defined in Section 5.3).

The following parameters will be summarized based information collected on the Day 5 visit:

- Number and percentage of patients who had one symptom of AHA (dark urine / yellow eyes),
- Time since start of *P. vivax* treatment (PQ/TQ)
- Number of AHA in total and per patient,
- Hemoglobin level, summary of other laboratory results, and change from baseline as available,
- AHA signs and symptoms,
- AHA treatments,
- Hospitalizations related to episode of AHA, duration of hospitalizations, and outcomes

In addition, the percentage of patients followed-up at Day 5 will be provided as well as the percentage of patients with AHA signs and details of signs summarized in the safety population and by anti-malarial treatment.

A listing of reported/confirmed cases of AHA will be produced.

DocUUID : cad3782b-e7e5-4e92-b6d8-aa95f83460f9

Case narratives for cases of confirmed AHA will be included in the study report.

5.6.8 Other Safety Analyses

A listing of pregnant female patients will be produced.

5.6.9 Malaria Follow-up at Day 5

Malaria follow-up at Day 5 will be presented using descriptive summary statistics as described in Section 5.1.1 for the overall analysis population and by anti-malarial treatment subgroups (defined in Section 5.3).

The percentage of patients with a follow-up visit on Day 5 will be summarized.

6 QUALITY AND VERSION CONTROL

All output will be validated and quality-controlled according to ICON standard operating procedures.

The initial approved version of the SAP will be noted as Version 1.0. Any changes to the SAP will be documented in a new version and the version number updated.

7 REFERENCES

1. Rajapakse S, Rodrigo C, Fernando SD. Tafenoquine for preventing relapse in people with Plasmodium vivax malaria. The Cochrane database of systematic reviews. 2015(4):Cd010458.
2. Ebstie YA, Abay SM, Tadesse WT, Ejigu DA. Tafenoquine and its potential in the treatment and relapse prevention of Plasmodium vivax malaria: the evidence to date. Drug design, development and therapy. 2016;10:2387-99.

Appendix 1: Schedule of Assessments

Assessments	Enrolment Visit	Follow-up Day 5[a] / any time after enrollment visit[b]
Informed consent for data use[b]	X[a]	
Inclusion/exclusion criteria	X[a]	
Clinic characteristics	X[a]	
Socio-demographics	X[a]	
Pregnancy status	X[a]	
Malaria history	X[a]	
Malaria diagnosis	X[a]	
Quantitative G6PD test	X[a]	
Hemoglobin level	X[a]	X[b]
Malaria treatments	X[a]	
Safety information: Signs of hemolysis (jaundice, dark urine, other)		X[ab]
Safety information: Acute hemolytic anemia[c]		X[b]

[a]Data collected from SIVEP form,
[b]Data collected from referral hospital,
[ab]Data collected from SIVEP form and/or from referral hospital

DocUUID : cad3782b-e7e5-4e92-b6d8-aa95f83460f9

Appendix 2: Planned Output

Table Number	Title
Table 1.1.0	Facility and patient characteristics by municipality
Table 1.1.1	Patient disposition by anti-malarial drug administered and overall (Summary)
Table 1.1.1.1	Patient disposition by anti-malarial drug administered and overall – Patients from Manaus
Table 1.1.1.2	Patient disposition by anti-malarial drug administered and overall – Patients from Porto Velho
Table 1.1.1.3	Patient disposition by anti-malarial drug administered and overall - New Pv infection within 60 days
Table 1.1.1.4	Patient disposition by anti-malarial drug administered and overall - Mixed Pf / Pv infections
Table 1.1.2	Patient disposition by anti-malarial drug administered and overall (primary population)
Table 1.2.1	Socio-demographic and baseline characteristics by anti-malarial drug administered and overall - Analysis population
Table 1.2.2	Socio-demographic and baseline characteristics by facility level and type and overall - Analysis population
Table 1.2.3	Socio-demographic and baseline characteristics by sex and overall - Analysis population
Table 1.2.4	Socio-demographic and baseline characteristics by new Pv infections within 60 days - Analysis population
Table 1.2.5	Socio-demographic and baseline characteristics by mixed Pf /Pv infections - Analysis population
Table 1.3.1	Malaria history and diagnosis by anti-malarial drug administered and overall - Analysis population
Table 1.3.1.1	Malaria history and diagnosis by anti-malarial drug administered and overall - Analysis population, Patients from Manaus
Table 1.3.1.2	Malaria history and diagnosis by anti-malarial drug administered and overall - Analysis population, Patients from Porto Velho
Table 1.3.2	Malaria history and diagnosis by facility level and type and overall - Analysis population
Table 1.3.3	Malaria history and diagnosis by facility level and type and overall – New Pv infection within 60 days - Analysis population
Table 1.3.4	Malaria history and diagnosis by facility level and type and overall – Mixed Pf / Pv infection - Analysis population
Table 1.4.1	G6PD tests and hemoglobin level by anti-malarial drug administered and overall - Analysis population
Table 1.4.1.1	G6PD tests and hemoglobin level by anti-malarial drug administered and overall - Analysis population, Patients from Manaus
Table 1.4.1.2	G6PD tests and hemoglobin level by anti-malarial drug administered and overall - Analysis population, Patients from Porto Velho
Table 1.4.2	G6PD tests and hemoglobin level by facility level and type and overall - Analysis population
Table 1.4.2.1	G6PD tests and hemoglobin level by facility level and type and overall - Analysis population, Patients from Manaus
Table 1.4.2.2	G6PD tests and hemoglobin level by facility level and type and overall - Analysis population, Patients from Porto Velho

DocUUID : cad3782b-e7e5-4e92-b6d8-aa95f83460f9

Table 1.4.3	G6PD tests and hemoglobin level by sex and overall - Analysis population
Table 1.4.4	G6PD tests and hemoglobin level by ethnic group and overall - Analysis population
Table 1.4.5	G6PD tests and hemoglobin level in pregnant women - Analysis population
Table 1.4.6	G6PD tests and hemoglobin level by anti-malarial drug administered and overall – New Pv infection within 60 days - Analysis population
Table 1.4.7	G6PD tests and hemoglobin level by anti-malarial drug administered and overall – Mixed Pf / Pv infection -Analysis population
Table 1.5.1	Multiple P. vivax episodes by anti-malarial drug administered and overall - Analysis population
Table 1.5.2	Multiple P. vivax episodes by sex and overall - Analysis population
Table 1.5.3	Multiple P. vivax episodes by weight category and overall - Analysis population
Table 1.5.4.1	Change in G6PD category status in same patients – Recurring population
Table 1.6.1	Malaria treatments by anti-malarial drug administered and overall - Analysis population
Table 1.6.1.1	Malaria treatments by anti-malarial drug administered and overall - Analysis population, Patients from Manaus
Table 1.6.1.2	Malaria treatments by anti-malarial drug administered and overall - Analysis population, Patients from Porto Velho
Table 1.6.2	Malaria treatments by facility level and type and overall - Analysis population
Table 1.6.2.1	Malaria treatments by facility level and type and overall - Analysis population, Patients from Manaus
Table 1.6.2.2	Malaria treatments by facility level and type and overall - Analysis population, Patients from Porto Velho
Table 1.6.3	Malaria treatments by facility level and overall – New vivax infection within 60 days
Table 1.6.4	Malaria treatments by facility level and overall – Mixed Pf / Pv infections
Table 2.1.1	Appropriate use and non-use of TQ by facility level and type and overall - Analysis population
Table 2.1.1.1	Appropriate use and non-use of TQ by facility level and type and overall - Analysis population, Patients from Manaus
Table 2.1.1.2	Appropriate use and non-use of TQ by facility level and type and overall - Analysis population, Patients from Porto Velho
Table 2.1.1.a	Characteristics of patients treated with TQ, by facility level and type and overall – Analysis population
Table 2.1.3	Appropriate use of TQ by facility level and type and overall (% facility level)- Analysis population
Table 2.1.3.1	Appropriate use of TQ by facility level and type and overall (% facility level) - Analysis population, Patients from Manaus
Table 2.1.3.2	Appropriate use of TQ by facility level and type and overall (% facility level) - Analysis population, Patients from Porto Velho
Table 2.2.1	Appropriate use and non-use of daily PQ by facility level and type and overall - Analysis population
Table 2.2.1.1	Characteristics of patients treated with PQ by facility level and type and overall - Analysis population, Patients from Manaus

DocUUID : cad3782b-e7e5-4e92-b6d8-aa95f83460f9

Table 2.2.1.2	Characteristics of patients treated with PQ by facility level and type and overall - Analysis population, Patients from Porto Velho
Table 2.2.1.a	Characteristics of patients treated with TQ by facility level and type and overall – New vivax infection in 60 days – Analysis population
Table 2.2.1.a	Characteristics of patients treated with PQ (daily, weekly) by facility level and type and overall - Analysis population
Table 2.2.2	Characteristics of patients treated with PQ by facility level and type and overall – New vivax infections in 60 days
Table 2.2.3	Appropriate use of PQ by facility level and type and overall (% facility level) - Analysis population
Table 2.2.3.1	Appropriate use of PQ by facility level and overall (% facility level) - Analysis population, Patients from Manaus
Table 2.2.3.2	Appropriate use of PQ by facility level and overall (% facility level) - Analysis population, Patients from Porto Velho
Table 2.2.3.3	Appropriate use of PQ in new vivax infections by facility level and overall (% facility level) - Analysis population
Table 2.3.1	Safety follow-up and signs of hemolysis by anti-malarial drug administered and overall – Safety population
Table 2.3.1.1	Safety follow-up and signs of hemolysis by anti-malarial drug administered and overall – Safety population, patients from Manaus
Table 2.3.1.2	Safety follow-up and signs of hemolysis by anti-malarial drug administered and overall – Safety population, patients from Porto Velho
Table 2.3.2	Safety follow-up and signs of hemolysis by facility level and type and overall – Safety population
**Figure Number**	**Title**
Figure 1.4.3.1	G6PD activity by category and sex - Analysis population (bar chart and box chart)
Figure 1.4.3.2	Hemoglobin level by sex – Analysis population (box chart)
Figure 1.5.1	Number of P. vivax recurrences by anti-malarial drug administered - Analysis population
**Listing Number**	**Title**
Listing 1.2.1	Demographic characteristics by municipality, treatment, and site / ID
Listing 1.2.2	G6PD activity in patients with low Hb (<7g /dL) and antimalarial treatment administered
Listing 1.5.1	Patients with multiple recurrences
Listing 2.1.1	Patients receiving inappropriate TQ treatment by municipality / location
Listing 2.1.2	Patients receiving inappropriate TQ treatment – new vivax infection in 60 days - by municipality / location
Listing 2.1.3	Patients receiving inappropriate TQ treatment – mixed Pf/Pv infection - by municipality / location
Listing 2.2.1	Patients receiving inappropriate PQ treatment by municipality / location (sorted by Municipality, grouped by category of inappropriate treatment)

Listing 2.3.1	Patients with signs of hemolysis at Day 5 by locality code and G6PD activity
Listing 2.3.2	Pregnant female patients

Tables provided for Interim Analysis

Appendix 3: Revision History

Version	Date	Author	Section	Main changes
V0.1	0AUG2020	Julie Duncan	N/A	N/A
V0.2	02OCT2020	Julie Duncan	N/A	Update according to sponsor comments (see MMV_TQ_SAP Brazil_v0.1 DRAFT - 28AUG2020 pg_kr_sd_JP_LT_AL pg_evc_cal pg)
V0.3	20JAN2021	Julie Duncan	N/A	Update according to sponsor meeting held on 15DEC2020
V0.4	25MAI2021	Julie Duncan	N/A	Update according to sponsor comments (see MMV_TQ_SAP Brazil_v0.3 DRAFT - 20JAN2021 26Feb2021 and MMV_TQ_TFL Shells Brazil_v0.3 DRAFT - 20JAN2021 26Feb2021)<br>Update of the section "Changes from Planned Analyses "<br>Several analyses performed in patients from Manaus and in patients from Porto Velho.
V0.5	27JULY2021	Julie Duncan	N/A	Update according to sponsor comments (see MMV_TQ_SAP Brazil_v0.4 DRAFT - 31MAY2021_mmv 2.docx and MMV_TQ_TFL Shells Brazil_v0.3 DRAFT 31MAY2021 mmv 1.docx)
V1.0	23AUGUST	Julie Duncan	N/A	Finalisation of document

DocUUID : cad3782b-e7e5-4e92-b6d8-aa95f83460f9

V1.1	13 May 2022	Julie Duncan	Various	Update to match protocol amendment, clarify appropriate use definitions and add subgroup for infection type
V1.2	27 June 2022	Julie Duncan	Various	Update subgroups, clarify definitions of correct application of treatment algorithm (TQ and PQ), remove description of HCPs who performed the G6PD test
V3.0	07 December 2022	Sally Trufan	Various	Update for Final Clinical Study Report analyses. Please see detailed list below:

Changes to SAP V1.2 to V3.0

P14: 4.3 Malaria treatments: Clarified that single dose PQ is categorized as "none" as the dose is inappropriate for Pv radical cure.

P14. 4.5. Safety assessments:

- Added information about the probabilistic linkage between SIVEP records and hospital records from the referral hospital and databases from other hospitals in the municipality.
- Added information about the probabilistic linkage between the SIVEP records and Mortality Information System.
- Clarified that all confirmed AHA are investigated, irrespective of the relationship with primaquine or tafenoquine.
- Added laboratory results and diagnosis (primary and secondary) to the information derived from the hospital investigation process.
- Added information reported in the case of death (treatment, time since treatment, G6PD status).
- Clarified that information about confirmed AHA cases and deaths are provided in detailed reports.
- Deleted the collection of HCP training records: Although the protocol included the linkage of HCP training records with cases of AHA, there is no value in doing so as the SIVEP form is not necessarily filled out by the person who performs the G6PD test / selects the treatment.

P16: 5.1.1.: Statistical rules and data formats

- Deleted the statement that percentages will be calculated based on the non-missing observations. This has been updated to include missing in the percentage calculation.

P17: 5.2 Analysis Sets

- Addition of a new sub-group for analysis: New infections within 60 days as they are not included in the primary endpoint.
- Clarification of safety population: All patients having received at least 1 dose of TQ or PQ will be considered in the safety population (original statement: All patients treated with TQ or PQ).

P.17 5.3 Subgroup analyses

- Clarification of Primary population (new cases)
- Revision of age classes:

DocUUID : cad3782b-e7e5-4e92-b6d8-aa95f83460f9

- Addition of sub-group of patients with positive mono vivax infection within 60 days and details of analysis.

DocUUID : cad3782b-e7e5-4e92-b6d8-aa95f83460f9
